# Enhanced magnetic moment discrimination for multiplex nanoparticle quantification via dual-frequency nonlinearity probing

**DOI:** 10.1038/s44172-026-00713-5

**Published:** 2026-06-30

**Authors:** Timur I. Bikulov, Ulrich M. Engelmann, Andreas Offenhäusser, Hans-Joachim Krause

**Affiliations:** 1 Institute of Biological Information Processing, Forschungszentrum Jülich, Jülich, Germany; 2 Faculty of Mathematics, Computer Sci. and Natural Sci., RWTH Aachen University, Aachen, Germany; 3 Department of Medical Engineering and Applied Mathematics, FH Aachen University of Applied Sciences, Jülich, Germany

**Keywords:** Biomedical engineering, Nanoscale biophysics, Nanoscale materials

## Abstract

Advanced applications of Magnetic nanoparticles (MNPs) in biomedicine based on multiplex MNP distinction require accurate, model-agnostic characterization of their magnetic moment distributions (MMDs). However, the resolving power of conventional MMD reconstruction from a static magnetization curve remains literarily underexplored. Moreover, due to particle-particle interactions, the response of the particle mixture might differ from the linear combination of the original constituents. We explore resolution enhancement in magnetic-moment space by directly probing higher-order magnetization derivatives, benefiting from their ever-increasing field-domain localization. Nonetheless, the direct derivative probing, as it is inevitably conducted dynamically, poses an interpretive problem for the origin of the nonlinearities. Spectral symmetries arising solely under dual-frequency excitation reflect the corresponding origins of amplitude- and rate-related nonlinearities. Using a dedicated experimental setup capable of synchronous demodulation of intermodulation terms, the method is tested on commercial MNP samples and benchmarked with conventional AC-susceptometry and static magnetization data. The binary mixture ratio was quantified with 8.9% deviation, without any prior information about the initial constituents differing by a factor of 3 in their average magnetic moments, potentially allowing the accommodation of three independent contrast channels for multiplex MNP applications as well as qualitatively probing magnetic interaction effects.

**Figure:**
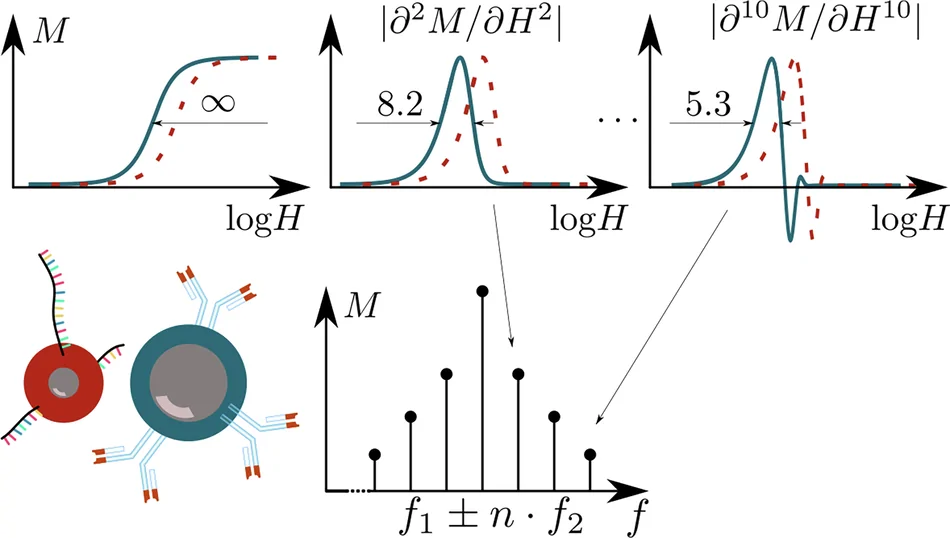

## Abstract (web summary)

Often overlooked, magnetic moment distribution-related batch variations in particle colloids result in experimental irreproducibility. Timur I. Bikulov and colleagues demonstrate model-agnostic resolution increase in magnetic moment domain using dual-frequency nonlinearity probing.

## Introduction

Single-domain magnetic nanoparticles (MNP) have found broad applications across diverse fields, including chemical processing^1^ and the oil industry^2,3^, as well as medicine^4,5^. Some noteworthy examples are: using static magnetic fields, MNPs are effectively employed for precision separation on the microscale, either to remove ionic contamination at parts-per-billion levels or to isolate cells directly from the bloodstream with efficiencies exceeding 70%^6–10^. Moreover, in response to periodically alternating magnetic fields (AMF), MNP can be remotely heated^2^ with an alternating magnetic field while providing immediate temperature feedback. This technology appears particularly promising for magnetic fluid hyperthermia (MFH)-based cancer therapy^4,11^, where cytostatic drugs can be selectively activated through local heating, while not exceeding 42 °C to maintain physiological functions. Finally, using the AMF-driven magnetic particle spectroscopy (MPS) read-out principle, Magnetic Particle Imaging (MPI) allows localization of as little as ~100 pg of MNP with a spatial resolution of $0.7\,{\rm{mm}}$^12,13^.

An auspicious approach is MPS’s unique multi-contrast capability, which exploits differences in MNP magnetic moments in response to an AMF to extract rich, contrast-enhanced information from heterogeneous particle ensembles. In the future, such a multicontrasting capability would, for instance, enable simultaneous remote viscosity/temperature measurement, as it provides two or more fully separated telemetry channels and magnetic moment (i.e., MNP core size) analysis based on MNP signal-generating capabilities in different contexts^14^. Foundational works by Shasha et al.^15^, Sun et al.^16^, and others^17–19^ imply the feasibility of this principle. However, this problem requires a method that yields the so-called core size distribution, which translates in magnetic measurements into a magnetic moment distribution (MMD) for the entire sample particle population. Reconstruction of MMD can be challenging due to a minor contamination of the sample with particles of larger core sizes that can significantly alter the nonlinear response, which is dominated by large particles^20^. At the same time, the methods of direct core size evaluation (such as TEM) do not allow non-destructive sampling of the whole population. In contrast, indirect methods (such as static magnetization measurement) lack sufficient resolution to precisely distinguish sizes, as explained below.

A common analysis of MNP magnetic cores employs transmission electron microscopy (TEM), particularly 3D TEM tomography. It offers^21^ sub-angstrom resolution ($< 0.5\,{{{{\AA}} }}$), which, when expressed as the magnetic moment of spherical magnetite, approaches the scale of a single Bohr magneton ($9.2\cdot {10}^{-24}\,{\rm{A}}{{\rm{m}}}^{2}$). TEM-image-based core size analysis commonly exhibits remarkably narrower size distributions (i.e., lower variance) than their magnetic moment counterparts derived from magnetic techniques using a static field^21^. This discrepancy is usually attributed to TEM’s inability to access MNP’s total magnetic moment, anisotropies, and inter-particle interactions. The latter two factors also shape the magnetization curve from which the magnetic moment distributions are indirectly obtained through a regularized inverse problem solution, unavoidably introducing a priori information by employing a static magnetization model, i.e., the Langevin curve^21^. Sometimes, to reduce the problem to an analytical solution, the distribution is inferred from statistical assumptions across the entire MNP ensemble^22^.

Berkov^23^ noted that the inverse problem for determining the magnetic moment distribution is a Fredholm equation of the first kind, which can be reformulated as a deconvolution problem. In this special case, applying the Fourier transform to the inputs simplifies the solution, as deconvolution reduces to an ordinary division. The convolutional form of an equation can be achieved by assuming odd symmetry in the magnetization curve and by switching to logarithmic variables for the fields and magnetic moments, since the magnetic moment and external field appear as a product in the equations. In other words, the information about magnetic moments is distributed not linearly, as in size measurements with TEM, but logarithmically, requiring logarithmic field sampling.

Moreover, Berkov draws attention to the biasing effects of regularization and advocates the use of low-level selection criteria, such as the uncorrelatedness of the fit error. Bender^24^ employs a similar approach for continuous Tikhonov regularization, aiming to reduce the influence of subjective factors. Nevertheless, the use of regularization is unavoidable due to the presence of noise in measured data. Noise causes deviations from an ideal monodisperse response, leading to peak broadening in the distribution. The degree of regularization sets the interpretation threshold for the noise level and, hence, determines the effective widths of the peaks and resolution in the magnetic moment space. The resolution limit, as analysis of the inverse problem in the Fourier space shows, arises from a limited spectral width of the static magnetization curve. Basically, the wider and smoother the shape of the static magnetization curve (deconvolution kernel), the more sensitive the solution becomes to random additive noise.

In this work, we propose that resolution improvement in magnetic-moment space can be achieved by directly probing the higher-order derivatives of the magnetization curve. Due to the sigmoidal topology of a magnetization curve, its derivatives get increasingly field-localized with increasing order. The direct assessment of a higher-order derivative is only accessible from a dynamic excitation of MNP by an alternating magnetic field, which, however, poses an additional problem of how to interpret the measured magnetic response. To address this challenge, this work provides an interpretational framework founded on modulation theory using basic geometrical assumptions about the dynamic magnetization trajectory of an MNP ensemble excited by an improved MPS technique using synchronized dual-frequency field, called frequency mixing magnetic detection (FMMD)^14^. This framework is assessed experimentally (using a setup described in “Setup of intermodulation spectral term measurement”) by measuring samples in liquid colloid state, as in this state, the inter-particle interactions can be controlled. By employing the dual-frequency measurement scheme, the colloid remains in a magnetization state that is asymptotically close to that of an ac-susceptometry measurement with a dc offset. That is why we verify the response of our proposed technique with static magnetization and ac-susceptometry as described in the Methods section. Two physical models of variable magnetic moment and variable media viscosity are employed to demonstrate the feasibility of directly interpreting their effects from the raw FMMD data. Finally, we experimentally demonstrate the resolution improvement in magnetic moment space by measuring the mixtures of different MNP types using the proposed technique. From this, we conclude that FMMD enables non-destructive, direct quantification of the magnetic moment distribution in unknown MNP samples without requiring prior assumptions on the sample properties. For a well-characterized MNP system, the framework could potentially provide a systematic approach to qualitatively detect the onset of interparticle magnetic interactions. Collectively, these capabilities would offer a foundation for enhancing quality control and assurance in MNP synthesis, characterization, and reproducibility assessment.

## Methods

### Theory of dual-frequency particle spectroscopy

This section provides the fundamental background necessary for interpreting dual-frequency MNP responses used in this work. Magnetic particle spectroscopy (MPS) commonly employs single-frequency alternating magnetic fields (AMF) with frequencies in the range of *f*_L _= 10–50,000 Hz and amplitudes up to ${h}_{{\rm{L}}}\le 50\,{\rm{mT}}/{\mu }_{0}$ to excite dynamic magnetic responses from MNP^25^. MPS can thereby directly detect the presence of superparamagnetic MNP, evident from their distinct magnetic moment exhibiting higher order harmonics $3{f}_{{\rm{L}}},5{f}_{{\rm{L}}},\ldots$, which are absent otherwise in biological tissues without MNP^26^. This pecularity allows MPS to selectively localize MNP by contrast-positive detection. These harmonics are typically extracted by synchronous demodulation with respect to the excitation field (EF). Interpretation commonly assumes a linear approximation of the MPS response^27^, where the signal amplitude of the fundamental harmonic ${f}_{{\rm{L}}}$ is proportional to the MNP concentration ${c}_{s}$, and energy dissipation is extracted from its phase^28^. However, as this work will demonstrate, such an interpretation is valid only under special conditions and does not readily generalize for higher-order harmonics with $n\cdot {f}_{{\rm{L}}},\,n\,{\mathbb{\in }}\,{\mathbb{N}}{\mathbb{ > }}1$. Instead, a deeper understanding of the physical origin shall be developed and applied here, as demonstrated in this section.

In a diluted MNP colloid, where particle-particle interaction is negligible, dissipative forces dominate over inertia, and restoring forces are not present. This prevalence ensures a non-resonant response from higher-order harmonic terms. Within this approximation, the emergence of the higher harmonics at the integer multiples of the fundamental harmonic can be solely attributed to the periodicity of the excitation field ${h}_{{\rm{e}}}\left(t\right)={h}_{{\rm{e}}}\left(t+n/{f}_{{\rm{L}}}\right),\,n\,{\mathbb{\in }}\,{\mathbb{Z}}$, which induces a similarly periodic response from the MNPs with ${m}_{{\rm{d}}}\left(t\right)={m}_{{\rm{d}}}\left(t+n/{f}_{{\rm{L}}}\right)$. The uniqueness of the period defines a unique phase within each excitation, from which the phases of the higher-order harmonics are coherently derived.

The emergence of higher harmonics is caused not only by *finite magnetizability*, i.e., the saturation magnetization of the MNP (i.e., magnetization is bounded $|{m}_{{\rm{d}}}\left(t\right)| < {\rm{const}}$ at large external field ${h}_{{\rm{e}}}\left(t\right)\to \infty$), but also *relaxation processes*. The latter can be described as a finite rate of change in magnetization $|\dot{{m}_{{\rm{d}}}}\left(t\right)| < {\rm{const}}$ in response to the external AMF of infinite rate $\dot{{h}_{{\rm{e}}}}\left(t\right)\to \infty$ (where dot-accent denotes the time-derivative, i.e., ${\dot{x}}={dx}{\scriptstyle{/}}{dt}$), meaning that at very high excitation frequency, the magnetization cannot follow the field anymore and gets distorted, too.

Two relaxation mechanisms are commonly known.

- The Néel relaxation (characterized by a time constant ${\tau }_{{\rm{N}}}$), which describes the internal reorientation of the particle’s magnetic moment inside the crystalline particle core.
- The Brownian relaxation (characterized by a time constant ${\tau }_{{\rm{B}}}$), which describes the external mechanical rotation of the entire particle relative to the surrounding medium.

Both mechanisms have been extensively studied, showing their simultaneous contribution to MNP’s nonlinear response. Relaxometry experiments conducted by Eberbeck^29^, El-Hilo^30^, and Porto^31^ show that MNP colloids can exhibit step responses of negative convexity on short time scales, which cannot be decomposed into a set of first-order model responses as mentioned in the comment of Maddox^32^ and reviewed in detail by Dormann^33^. Small-amplitude ac susceptometry measurements conducted by Petracic et al.^34^ demonstrate a broad distribution of relaxation times, resembling the behavior of a fractional order system. Theoretical analysis of the Langevin equation by Coffey et al.^35^ using the Fokker-Planck equation shows that the characteristic relaxation time of an MNP system depends on both the initial and final values of the applied field.

Therefore, we can conclude that both finite magnetizability and relaxation effects contribute to each higher harmonic simultaneously. This superposition is overlooked in a simplified linear interpretation of the MPS response. However, these effects can be distinguished by exploiting their field-related symmetry properties: finite magnetizability is an odd-symmetric process with respect to the field ${h}_{{\rm{e}}}\left(t\right)$, whereas the relaxation is even-symmetric and, due to causality, sign-constant.

These symmetries manifest as corresponding spectral symmetries of higher-order terms if AMF includes a second harmonic excitation component. In a single-frequency excitation, the field amplitude, ${h}_{{\rm{e}}}\left(t\right)$, and its rate, $\dot{{h}_{{\rm{e}}}}\left(t\right)$, are tightly coupled: $\dot{{h}_{{\rm{e}}}}\left(t\right)$ is minimal when ${h}_{{\rm{e}}}\left(t\right)$ is at its extrema and vice versa. The instantaneous amplitude of a harmonic signal follows an arcsine distribution, where the ${h}_{{\rm{e}}}\left(t\right)$ remains within 76% to 100% of its peak value for 90% of the time. As a result, the nonlinear responses of MNP due to field amplitude and its rate become entangled under single-frequency excitation, and the information derived in MPS from the MNP response via the harmonic terms is diminished. To enhance the physical interpretability of the MNP response, it is thus essential to disentangle the effects of ${h}_{{\rm{e}}}\left(t\right)$ and $\dot{{h}_{{\rm{e}}}}\left(t\right)$. The distinction can be achieved by adding a second harmonic component to the excitation signal. This technique, known as frequency mixing magnetic detection (FMMD), was proposed by Krause et al.^36^ and Nikitin^37^ and has since been further developed by many other research groups^38–42^.

In FMMD, the excitation signal ${h}_{{\rm{e}}}\left(t\right)$ consists of two harmonic components of high- (H) and low frequency (L). Additionally, allowing a DC offset field, ${h}_{\rm{DC}}$, for generality, the excitation AMF reads:

1 $${h}_{{\rm{e}}}\left(t\right)={h}_{{\rm{DC}}}+{h}_{{\rm{H}}}\cos \left(2\pi {f}_{{\rm{H}}}t+{\varphi }_{{\rm{H}}}\right)+{h}_{{\rm{L}}}\cos \left(2\pi {f}_{{\rm{L}}}t+{\varphi }_{{\rm{L}}}\right)$$

Assuming no DC offset field (${h}_{{\rm{DC}}}=0$), the combination of amplitudes ${h}_{{\rm{H}}}$, ${h}_{{\rm{L}}}$, and frequencies ${f}_{{\rm{H}}}$, ${f}_{{\rm{L}}}$, can be chosen to fulfill:

2 $$\left\{\begin{array}{c}{h}_{{\rm{L}}} > {h}_{{\rm{H}}}\\ {h}_{{\rm{H}}}{f}_{{\rm{H}}}\,{\gg \,h}_{{\rm{L}}}{f}_{{\rm{L}}}\end{array}\right.$$

Under these conditions, the amplitude of ${h}_{{\rm{e}}}\left(t\right)$ is dominated by the low-frequency component, ${h}_{{\rm{e}}}\left(t\right)\approx {h}_{{\rm{L}}}\cos (2\pi {f}_{{\rm{L}}}t)$, and its rate is dominated by the high-frequency component, $\dot{{h}_{{\rm{e}}}\left(t\right)}\approx 2\pi {f}_{{\rm{H}}}\cdot {h}_{{\rm{H}}}\sin (2\pi {f}_{{\rm{H}}}t)$.

When exciting an MNP colloid with such a dual-frequency field ${h}_{{\rm{e}}}\left(t\right)$ the resulting spectral response contains all combinations of integer harmonic terms of the form $n{f}_{{\rm{H}}}+m{f}_{{\rm{L}}}$ with $\left(n,m\right)\,{\mathbb{\in }}\,{\mathbb{Z}}$. A Fourier component of ${m}_{{\rm{d}}}\left(t\right)$ corresponding to frequency $n{f}_{{\rm{H}}}+m{f}_{{\rm{L}}}$ will be denoted further as ${A}_{n,m}\,{\mathbb{\in }}\,{\mathbb{C}}$.

These intermodulation terms (IMT) with $(n,m)\,\ne \,0$ are directly accessible via hardware described in “Setup of intermodulation spectral term measurement” or via standard MPS techniques. If ${f}_{{\rm{H}}}$ is derived from ${f}_{{\rm{L}}}$, i.e., using phase-locked loop, so ${f}_{{\rm{H}}}\,={n}_{{\rm{f}}}{f}_{{\rm{L}}},$ with ${n}_{{\rm{f}}}\,{\mathbb{\in }}\,{\mathbb{N}}$ then the measurement of the intermodulation term (IMT) reduces to the measurement of the higher-order term ${f}_{{\rm{H}}}+m{f}_{{\rm{L}}}=({n}_{{\rm{f}}}+m){f}_{{\rm{L}}}$. A detailed review of demodulation schemes for IMT measurement was conducted by Ruppert^43^ in the context of multifrequency atomic force microscopy. The frequency band ${f}_{{\rm{H}}}\pm m{f}_{{\rm{L}}}$ around the high-frequency fundamental harmonic ${f}_{{\rm{H}}}$ is particularly useful for characterizing MNP properties due to three reasons:

- Dense harmonic packing: applying a low frequency (e.g., ${f}_{{\rm{L}}} \sim 10\,{\rm{Hz}}$), densely packs higher harmonics within a narrow frequency band, simplifying hardware filtering (e.g., requiring just a single analog filter).
- Nonlinearity specificity: the terms ${A}_{1,2m}$ (with $m\,\ne \, 0$) maximize when the signal’s amplitude remains in the region of high nonlinearity of the magnetic moment, i.e., ${d}^{2}{m}_{{\rm{d}}}\left(t\right)/d{h}_{{\rm{e}}}^{2}\left(t\right)\, \ne \,0$.
- Full-cycle reconstruction: the band contains harmonics of all orders, including the first one, enabling the reconstruction of the absolute values of all derivatives associated with the magnetization cycle *M*(*H*), as shown later in this section.

The signal in ${f}_{{\rm{H}}}\pm m{f}_{{\rm{L}}}$ band has the form of an amplitude- and phase-modulated (AM/PM) signal with carrier ${f}_{{\rm{H}}}$ and a complex envelope with a period of $1/{f}_{{\rm{L}}}$. Any pair of independent harmonics can be expressed in this form, as proposed by Kalb and Bennet in their analysis on nonlinear distortions in telegraph lines^44^,

3 $$P\cos \left({pt}\right)	+Q\cos \left({qt}\right)\\ 	 =\sqrt{{\left(P+Q\right)}^{2}-4{PQ}{\sin }^{2}\left(\frac{p-q}{2}t\right)}\\ 	 \times \cos \left(\frac{p+q}{2}t+{\rm{atan}}\left(\frac{P-Q}{P+Q}\tan \left(\frac{p-q}{2}t\right)\right)\right)$$

with the carrier frequency ${f}_{{\rm{c}}}=\left(p+q\right)/2$. Pairwise application of this operation to all symmetrical harmonics around ${f}_{{\rm{H}}}$ leads to the instantaneous representation ${\hat{m}}_{{\rm{d}}}\left(t\right)$ of the signal in the ${f}_{{\rm{H}}}+m{f}_{{\rm{L}}}$ band, in the AM/PM form,

4 $${\hat{m}}_{{\rm{d}}}\left(t\right)={\hat{a}}_{{\rm{d}}}\left(t\right)\cos \left(2\pi {f}_{{\rm{H}}}t+{\hat{\varphi }}_{{\rm{d}}}\left(t\right)\right)$$

**Fig. 1:**
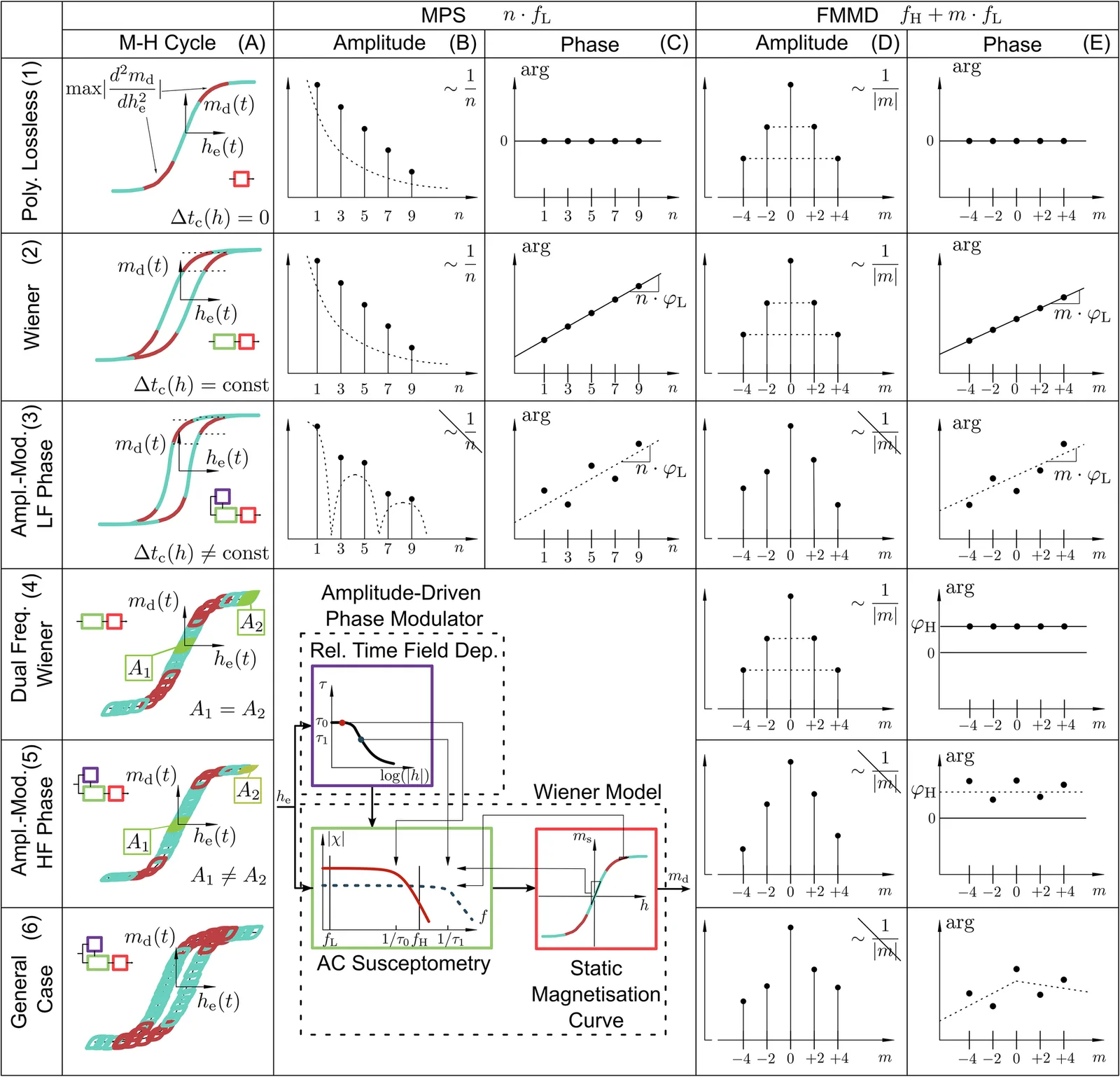
Symmetries in MPS/FMMD M(H)-cycles and their corresponding spectra across different models. Column (**A**)—M(H)-cycle on MH plane. Red regions show regions with maximum curvature. Columns (**B**) and (**C**)—the amplitude and phase of the Magnetic Particle Spectroscopy (MPS) signal. Columns (**D**) and (**E**)—the amplitude and phase of the frequency mixing magnetic detection (FMMD) signal in the frequency range around ${f}_{{\rm{H}}}$. Solid lines on columns (**C**) and (**E**) denote the linear dependence of the phase of the higher-order spectral term as a function of its order: ${\mbox{arg}}({A}_{\cdot ,n})\propto n$. The schematic inset at the bottom center depicts the signal model for FMMD interconnecting the static magnetization curve (red), the AC susceptibility (green), and the relaxation time field dependence (violet).

Being inspired by the work of Platz et al.^45^ where a physical interpretation of complex envelope is given for multi-frequency atomic force microscopy, we propose that the complex envelope ${\hat{a}}_{{\rm{d}}}\left(t\right){e}^{j{\hat{\varphi }}_{{\rm{d}}}\left(t\right)}$ offers an intuitive physical interpretation of the MPS signal by separating the finite magnetization from the relaxation process: The signal’s instantaneous amplitude ${\hat{a}}_{{\rm{d}}}\left(t\right)$ reflects the static magnetization behavior, while the instantaneous phase ${\hat{\varphi }}_{{\rm{d}}}\left(t\right)$ encodes the relaxation process. The spectral symmetries associated with this decomposition are summarized in Fig. 1. Column (A) displays the system’s magnetization cycle in the M-H plane, where the enclosed hysteresis area corresponds to the energy dissipated per EF cycle. The red regions highlight the points of maximum curvature. Columns (B) and (C) depict the corresponding single-frequency MPS response, while columns (D) and (E) show the FMMD responses in the working band ${f}_{{\rm{H}}}\pm m{f}_{{\rm{L}}}$.

The first row (1) in Fig. 1 corresponds to a case of the equilibrium model, where both excitation frequencies ${f}_{{\rm{H}}}$ and ${f}_{{\rm{L}}}$ are sufficiently low such that the magnetization process is adiabatic, i.e., ${\tau }_{{\rm{N}}},{\tau }_{{\rm{B}}}\,\ll \,1/{f}_{{\rm{H}}}$ is fulfilled. Here, the complex envelope reduces to a solely real amplitude component ${\hat{a}}_{{\rm{d}}}\left(t\right)$ with zero instantaneous phase $({\hat{\varphi }}_{{\rm{d}}}\left(t\right)=0)$. Hysteresis is absent on the M-H plane, and nonlinear distortions are determined solely by finite magnetizability effects. The equilibrium model serves as a starting point for the analysis of more complex dynamic cases later, which can be interpreted as deviations from this idealized case. The model provides a framework for understanding how information from the M-H curve is distributed across spectral terms, whose number is ultimately limited by a system’s noise floor. A polynomial model is one possible representation illustrating that the number of derivatives of the M-H curve determines the number of resolvable spectral terms. According to Weierstraß theorem, any single-valued M-H curve can be approximated over a finite magnetic field range $\left[-{h}_{\max },{h}_{\max }\right],\,{h}_{\max }={h}_{{\rm{DC}}}+{h}_{{\rm{H}}}+{h}_{{\rm{L}}}$ by a polynomial of ${L}_{{\rm{poly}}}$ terms^46^

5 $${m}_{{\rm{d}}}\left(t\right)=\,	{m}_{{\rm{s}}}\left({h}_{{\rm{e}}}\left(t\right)\right)\,\\ {m}_{{\rm{s}}}\left(h\right)=	\mathop{\sum }\limits_{i=0}^{{L}_{{\rm{poly}}}-1}{a}_{2i+1}^{{\rm{ms}}}{h}^{2i+1}$$

Only odd terms contribute to the polynomial since the M-H curve is odd-symmetric. Using multinomial expansion, a Fourier series can be obtained for dynamic magnetization ${m}_{{\rm{d}}}\left(t\right)$ as follows,

6 $${m}_{{\rm{d}}}\left(t\right)=	\mathop{\sum }\limits_{n=0}^{{L}_{{\rm{poly}}}-1}\frac{{a}_{2n+1}^{{\rm{ms}}}}{{2}^{\left(2n+1\right)}}{{\rm{Re}}}\left[\mathop{\sum }\limits_{{k}_{1}+{k}_{2}+{k}_{3}+{k}_{4}+{k}_{5}=2n+1}{A}^{{\rm{poly}}}\cdot {P}^{{\rm{poly}}}\cdot {F}^{{\rm{poly}}}\right]\\ {A}^{{\rm{poly}}}:=	\frac{\left(2n+1\right)!}{{k}_{1}!{k}_{2}!{k}_{3}!{k}_{4}!{k}_{5}!}{\left(2{h}_{{\rm{DC}}}\right)}^{{k}_{5}}{{h}_{{\rm{H}}}}^{{k}_{1}+{k}_{2}}{{h}_{{\rm{L}}}}^{{k}_{3}+{k}_{4}}\,\\ {P}^{{\rm{poly}}}:=	\exp \left({\rm{i}}\left({\varphi }_{{\rm{H}}}\left({k}_{1}-{k}_{2}\right)+{\varphi }_{{\rm{L}}}\left({k}_{3}-{k}_{4}\right)\right)\right)\\ {F}^{{\rm{poly}}}:=	\exp \left({\rm{i}}2\pi t\left({f}_{{\rm{H}}}\left({k}_{1}-{k}_{2}\right)+{f}_{{\rm{L}}}\left({k}_{3}-{k}_{4}\right)\right)\right)$$

where “:=” denotes substitution. The nested summation is conducted over all terms ${k}_{i}\,{\mathbb{\in }}\,{\mathbb{Z}}{\mathbb{\ge }}0$, that satisfy equality ${k}_{1}+{k}_{2}+{k}_{3}+{k}_{4}+{k}_{5}=2n+1$. By applying additional constraints on the summation, one obtains the amplitude and the phase of the intermodulation term (IMT) ${A}_{m,p}$ of interest, reading

7 $${A}_{m,p}=\mathop{\sum }\limits_{n=0}^{{L}_{{\rm{poly}}}-1}\frac{{a}_{2n+1}^{{\rm{ms}}}}{{2}^{\left(2n+1\right)}}\mathop{\sum }\limits_{\left\{\begin{array}{c}{k}_{1}+{k}_{2}+{k}_{3}+{k}_{4}+{k}_{5}=2n+1\\ {k}_{2}-{k}_{1}=m\\ {k}_{4}-{k}_{3}=p\end{array}\right.}{A}^{{\rm{poly}}}$$

Once expressed in matrix form $\{{g}_{i,j}\}$, sparsity analysis of Eq. (7) explicitly demonstrates how information about the derivatives of the M-H curve is distributed across higher-order harmonics.

8 $$\left(\begin{array}{ccc}\begin{array}{ccc}\begin{array}{c}{g}_{1,1}\\ 0\\ 0\\ 0\\ 0\\ \vdots \\ 0\\ 0\\ {g}_{{n}_{{\rm{f}}},1}\\ 0\\ 0\\ 0\end{array} & \begin{array}{c}{g}_{1,2}\\ {g}_{2,2}\\ {g}_{3,2}\\ 0\\ 0\\ \vdots \\ {g}_{\left({n}_{{\rm{f}}}-2\right),2}\\ {g}_{\left({n}_{{\rm{f}}}-1\right),2}\\ {g}_{{n}_{{\rm{f}}},2}\\ {g}_{\left({n}_{{\rm{f}}}+1\right),2}\\ {g}_{\left({n}_{{\rm{f}}}+2\right),2}\\ 0\end{array} & \begin{array}{c}{g}_{1,3}\\ {g}_{2,3}\\ {g}_{3,3}\\ {g}_{4,3}\\ {g}_{5,3}\\ \vdots \\ {g}_{\left({n}_{{\rm{f}}}-2\right),3}\\ {g}_{\left({n}_{{\rm{f}}}-1\right),3}\\ {g}_{{n}_{{\rm{f}}},3}\\ {g}_{\left({n}_{{\rm{f}}}+1\right),3}\\ {g}_{\left({n}_{{\rm{f}}}+2\right),3}\\ {g}_{\left({n}_{{\rm{f}}}+3\right),3}\end{array}\end{array} & \begin{array}{c}\cdots \\ \cdots \\ \cdots \\ \cdots \\ \cdots \\ \ddots \\ \cdots \\ \cdots \\ \cdots \\ \cdots \\ \cdots \\ \cdots \end{array} & \begin{array}{c}{g}_{1,L}\\ {g}_{2,L}\\ {g}_{3,L}\\ {g}_{4,L}\\ {g}_{5,L}\\ \vdots \\ {g}_{\left({n}_{{\rm{f}}}-2\right),L}\\ {g}_{\left({n}_{{\rm{f}}}-1\right),L}\\ {g}_{{n}_{{\rm{f}}},L}\\ {g}_{\left({n}_{{\rm{f}}}+1\right),L}\\ {g}_{\left({n}_{{\rm{f}}}+2\right),L}\\ {g}_{\left({n}_{{\rm{f}}}+3\right),L}\end{array}\end{array}\right)\left(\begin{array}{c}{a}_{1}^{{\rm{ms}}}\\ {a}_{3}^{{\rm{ms}}}\\ {a}_{5}^{{\rm{ms}}}\\ {a}_{7}^{{\rm{ms}}}\\ \vdots \\ {a}_{L}^{{\rm{ms}}}\end{array}\right)=\left(\begin{array}{c}{A}_{0,1}\\ {A}_{0,2}\\ {A}_{0,3}\\ {A}_{0,4}\\ {A}_{0,5}\\ \vdots \\ {A}_{1,-2}\\ {A}_{1,-1}\\ {A}_{1,0}\\ {A}_{1,1}\\ {A}_{1,2}\\ {A}_{1,3}\end{array}\right)$$

Several practically useful conclusions can be drawn from the equilibrium model about phases and amplitudes of IMTs:

- Amplitude is independent of phase: The IMT’s amplitude $|{A}_{m,p}|$ is independent of the excitation field phases ${\varphi }_{{\rm{H}}}$ and ${\varphi }_{{\rm{L}}}$.
- Phase linearity: The phase of IMT, ${\rm{arg}}({A}_{m,p})$, is independent of the excitation field amplitudes, ${h}_{{\rm{DC}}},{h}_{{\rm{H}}}$ and$\,{h}_{{\rm{L}}}$, is given by a linear combination of original excitation field phases: ${\rm{arg}}({A}_{m,p})=m{\varphi }_{{\rm{H}}}+p{\varphi }_{{\rm{L}}}$.
- Spectral symmetry: IMTs exhibit symmetry around the fundamental harmonics, $|{A}_{m,p}|\,=|{A}_{m,-p}|$, as illustrated by the dashed line in Fig. 1 D1.
- Information content per harmonic order: The higher the order $\left|m\right|+\left|p\right|$ of an IMT, ${A}_{m,p}$, the fewer polynomial coefficients ${a}_{2n+1}^{{\rm{ms}}}$ contribute to its formation, and consequently, less information about the M-H trajectory is contained.
- Fundamental frequency responses are necessary: At least the response at one fundamental frequency (${A}_{1,0}$ or ${A}_{0,1}$) is required to reconstruct the M-H trajectory uniquely and to determine the hysteresis area.
- Matrix structure reveals cross-correlation: The triangular shape of the IMT matrix suggests the existence of M-H-curve-independent cross-correlations between spectral lines, which can potentially be exploited for noise suppression by merging this cross-correlated data.
- Effect of DC-offset field: The application of an offset field, ${h}_{{\rm{DC}}}$, leads to the emergence of even-order harmonics and a redistribution of spectral energy between neighboring harmonics ${A}_{1,p}$ and ${A}_{1,p-1}$ ($p\,\ne \,0$) without affecting the underlying matrix sparsity.

The use of the Bessel function apparatus and the Fourier transform avoids the need to solve the subset-sum problem in further analysis of Eq. (6), enabling a direct connection between the particle’s magnetic moments and the complex envelope. One can approximate the M-H curve using a truncated sine series up to the ${L}_{{\rm{sine}}}^{\rm{th}}$ term on the same limited interval as the polynomial model,

9 $${m}_{{\rm{s}}}\left(h\right)=\mathop{\sum }\limits_{n=1}^{{L}_{{\rm{sine}}}}{c}_{n}^{{\rm{ms}}}\cdot \sin \left(n\pi \frac{h}{{h}_{\max }}\right)$$

where ${c}_{n}^{{\rm{ms}}}\,{\mathbb{\in }}\,{\mathbb{R}}$ are real-valued coefficients of the sine transform of the M-H curve. The expression for the magnetic moment from Eq. (6) can now be rewritten using the complex Jacobi-Anger expansion of Bessel functions,

10 $${m}_{{\rm{d}}}\left(t\right)=	\mathop{\sum }\limits_{l=1}^{{L}_{{\rm{sine}}}}{c}_{l}^{{\rm{ms}}}\cdot {\rm{Im}}\left({e}^{{\rm{i}}l\pi \frac{{h}_{{\rm{DC}}}}{{h}_{\max }}}\right.\mathop{\sum }\limits_{n=-\infty }^{\infty }\mathop{\sum }\limits_{m=-\infty }^{\infty }\,\\ 	 \cdot {{\rm{i}}}^{n+m}{J}_{n}\left(l\pi \frac{{h}_{{\rm{H}}}}{{h}_{\max }}\right){J}_{m}\left(l\pi \frac{{h}_{{\rm{L}}}}{{h}_{\max }}\right)\left.{e}^{{\rm{i}}\left(n\left(2\pi {f}_{{\rm{H}}}t+{\varphi }_{{\rm{H}}}\right)+m\left(2\pi {f}_{{\rm{L}}}t+{\varphi }_{{\rm{L}}}\right)\right)}\right)$$

where ${J}_{n}\left(x\right)$ denotes the Bessel functions of the first kind. From this, the odd-order IMT ${A}_{1,\pm {2k}_{2}}$ can be expressed directly as,

11 $${A}_{1,\pm {2k}_{2}}=	\mathop{\sum }\limits_{n=1}^{{L}_{{\rm{sine}}}}{c}_{n}^{{\rm{ms}}}\cos \left(n\pi \frac{{h}_{{\rm{DC}}}}{{h}_{\max }}\right)\cdot 2\\ 	 \cdot {J}_{2{k}_{2}}\left(n\pi \frac{{h}_{{\rm{L}}}}{{h}_{\max }}\right)\cdot {J}_{1}\left(n\pi \frac{{h}_{{\rm{H}}}}{{h}_{\max }}\right)\cdot {e}^{j\left({\varphi }_{{\rm{H}}}\pm {2k}_{2}{\varphi }_{{\rm{L}}}\right)}$$

A sparsity analysis of Eq. (11) reveals structural parallels between ${c}_{n}^{{\rm{ms}}}$ and ${a}_{2n+1}^{{\rm{ms}}}$, especially in correlation between the order of the coefficients and the number of the intermodulation terms (IMTs) which they determine. An analytical expression that explicitly links the magnetic moment of an MNP to the IMT’s amplitude and provides terms for the series Eq. (11) can be obtained from the fundamental work of Rice^47^, which discusses the propagation of harmonic signals through a nonlinear lumped-parameter system, and from the Fourier transform of the Langevin curve provided by Maass and Martens^48^.

As this relationship is expressed as a function of the low-frequency excitation field amplitude (${h}_{L}$), it provides a quantitative framework for interpreting MNP dynamics in response to a dual-frequency excitation field:

12 $$\left|{\hat{A}}_{{k}_{1},{k}_{2}}({h}_{{\rm{L}}},{\mu }_{p})\right| = 	 \left|\frac{2}{\pi }{\int }_{0}^{\infty }{\hat{F}}_{L}({f}_{h},{\mu }_{p})\cdot {J}_{{k}_{1}}\left({h}_{{\rm{H}}}{f}_{h}\right){\cdot J}_{{k}_{2}}\left({h}_{{\rm{L}}}{f}_{h}\right){\rm{d}}{f}_{h}\right| \\ \hat{{{\mathcal{L}}}}\left({f}_{h}\right) = 	 -i\frac{{f}_{h}}{\left|{f}_{h}\right|}\frac{2\pi {e}^{-\pi \left|{f}_{h}\right|}}{1-{e}^{-\pi \left|{f}_{h}\right|}},\left|{f}_{h}\right|\,\ne \,0 \\ {\hat{F}}_{L}({f}_{h},{\mu }_{p}) = 	 \frac{1}{\left|{\alpha }_{p}\right|}\hat{{{\mathcal{L}}}}\left(\frac{{f}_{h}}{{\alpha }_{p}}\right),{\alpha }_{p}=\frac{{\mu }_{p}{\mu }_{0}}{{k}_{B}T}$$

Here, ${f}_{h}$ is the frequency in the field domain, and ${\hat{F}}_{L}({f}_{h},{\mu }_{p})$ denotes the Fourier transform of the Langevin curve of the monodisperse sample with magnetic moment of ${\mu }_{p}$. The Fourier coefficients for Eq. (11) can be derived from the continuous transform ${c}_{n}^{{\rm{ms}}}=\hat{{{\mathcal{L}}}}(n\pi {\scriptstyle{/}}{h}_{\max }{\alpha }_{p}){\scriptstyle{/}}|{\alpha }_{p}{h}_{\max }|$.

**Fig. 2:**
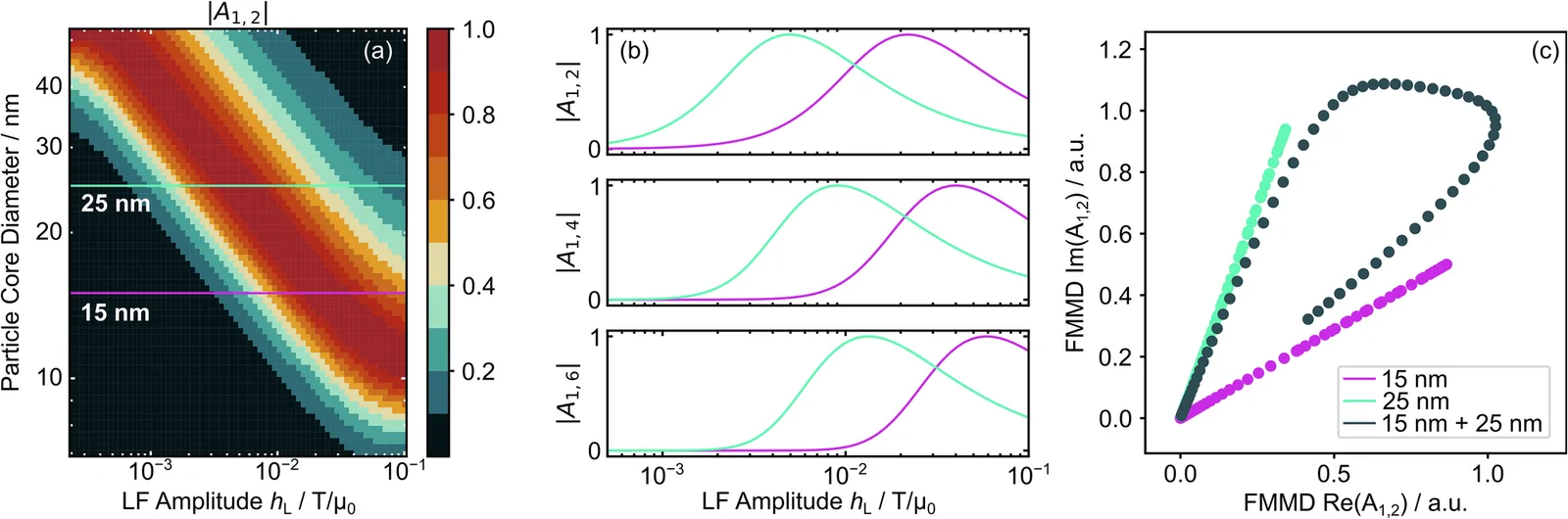
Direct mapping of the MNP’s magnetic moment in dependence on low-frequency amplitude (LF), *h*_*L*_. **a** Component $|{A}_{1,2}|$ was calculated for different magnetic moments with varying ${h}_{L}$. Solid lines show monodisperse sample with a magnetic moment equivalent to 15 nm and 25 nm, assuming spherical MNP of magnetite (${M}_{s}=476\,{\mbox{kA}}/{\mbox{m}}$). **b** LF scans for the higher-order components $|{A}_{1,2}|,|{A}_{1,4}|$, and $|{A}_{1,6}|$. **c** Example of the dependence ${\rm{arg}}({A}_{1,2})=f({h}_{L})$ arising from mixing two samples (15 nm and 25 nm) for which the Wiener model $(\triangle {t}_{{\mbox{c}}}={\mbox{const}})$ is valid (cf. Fig. 1 A4).

Numerical analysis of Eq. (12) shows that by varying ${h}_{{\rm{L}}}$, a direct mapping between the amplitude peak in IMT measurement and the magnetic moment ${\mu }_{p}$ can be established. Figure 2a illustrates this mapping by plotting the normalized IMT amplitude $|{A}_{1,2}|$ as a function of ${h}_{{\rm{L}}}$ (with ${h}_{{\rm{H}}}=1\,{\rm{mT}}$). Here, $|{A}_{1,2}|$ is a function of the magnetic moment (assuming MNP of spherical magnetite core with saturation magnetization of ${M}_{s}=476\,{\rm{kA}}{{\rm{m}}}^{-1}$). The resulting mapping matrix exhibits a Toeplitz-like structure that remains invariant with respect to ${h}_{{\rm{H}}}$ as long as the low-frequency component is dominant, cf. Eq. (2). Outside of Eq. (2) requirements, deviations from the diagonal structure are observed at the matrix boundaries for high fields & small MNP or low fields and large MNP. In Fig. 2a, two horizontal lines indicate magnetic moments corresponding to MNPs with core diameters of 15 nm (red) and 25 nm (green). As Fig. 2b illustrates, their corresponding higher-order IMTs ($|{A}_{1,2}|,|{A}_{1,4}|$, and $|{A}_{1,6}|$) are mutually cross-correlated, enabling the simultaneous probing of multiple magnetic moments when several IMTs are measured concurrently.

The selection of a magnetization model at this stage inherently introduces a priori assumptions about the sample’s magnetic behavior. Despite its widespread use, the Langevin curve remains inadequate for particle systems with finite magnetic anisotropy energy. Such particle systems can exhibit additional nonlinear magnetization features that, if interpreted using the Langevin curve, may be erroneously attributed to non-physical components in the magnetic moment distribution and corrupt correct moment distribution reconstruction. For such cases, an equilibrium magnetization model with uniaxial anisotropy, as proposed by Madsen^49^, provides a more suitable description that incorporates anisotropy effects in their simplest form. Note that magnetic interparticle interactions can similarly introduce energy barriers that mimic the effects of enhanced anisotropy, i.e., as discussed by Yanes et al.^50^. The problem of interpretation of static magnetization curve shape goes beyond the scope of this paper, as at this stage, we aim to demonstrate the correspondence between FMMD measurement and other standard techniques.

To extend the equilibrium model for the dynamic magnetic response of MNP, the most straightforward approach is to augment the static nonlinear response element with a linear dynamic element, as shown in the schematic inset in Fig. 1 (red box: nonlinear static element; green box: linear dynamic element). This configuration introduces a constant steady-state delay $\triangle {t}_{{\rm{c}}}$ relative to the excitation field ${h}_{e}(t)$, resulting in a constant dynamic phase ${\hat{\varphi }}_{{\rm{d}}}\left(t\right)$ with preserved spectral symmetry of the magnetic response. Such a configuration, called the Wiener model^51^, is suggested as the MNP colloid responds to frequencies below its cut-off frequency but can generate spectral components beyond this frequency. In this model, the EF harmonics passing through the linear element acquire phases ${\varphi }_{{\rm{H}}}$ and ${\varphi }_{{\rm{L}}}$, whose corresponding spectral effects—assuming that one of the phases equals zero—are illustrated in rows 2 and 4 of Fig. 1, respectively. In the complex plane, the system’s response under variation of ${h}_{{\rm{L}}}$ follows a constant linear trajectory (${\rm{arg}}({A}_{1,2})={\rm{const}}$), as observed for the magenta and green exemplary samples in Fig. 2c. If within this model ${\rm{arg}}({A}_{\cdot ,n})\propto n$ holds, it becomes feasible to extrapolate the phase of the fundamental harmonic (${A}_{1,0}$ or ${A}_{0,1}$) from measuring the higher-order IMTs. Direct measurement of fundamental harmonics is usually obstructed by background interference.

An even more sophisticated dynamic extension of the equilibrium model accounts for the field dependence of the Néel and Brownian relaxation times, ${\tau }_{{\rm{N}}}$ and ${\tau }_{{\rm{B}}}$, when the instantaneous phase is no longer constant (${\hat{\varphi }}_{{\rm{d}}}\left(t\right)\,\ne \,{\rm{const}}$). The necessity of this extension becomes relevant when ${\tau }_{{\rm{N}}}$ or ${\tau }_{{\rm{B}}}$ approaches the characteristic time of the high-frequency excitation signal, i.e., ${\tau }_{{\rm{N}}}$ or $\,{\tau }_{{\rm{B}}}\approx 1/{f}_{{\rm{H}}}$. In the model shown in Fig. 1, this effect is represented by a violet element, indicating the dependence of the small-signal relaxation time on the offset field. These amplitude-driven variations of relaxation time lead to three decisive consequences for the dynamic magnetic response of the MNP:

- redistribution of the nonlinearity regions in (cf. Fig. 1 A3, A5),
- breaking of the amplitude symmetries (cf. Fig. 1 D3, D5)
- breaking of linear dependencies of the phases (cf. Fig. 1 C3, E3, E5).

**Fig. 3:**
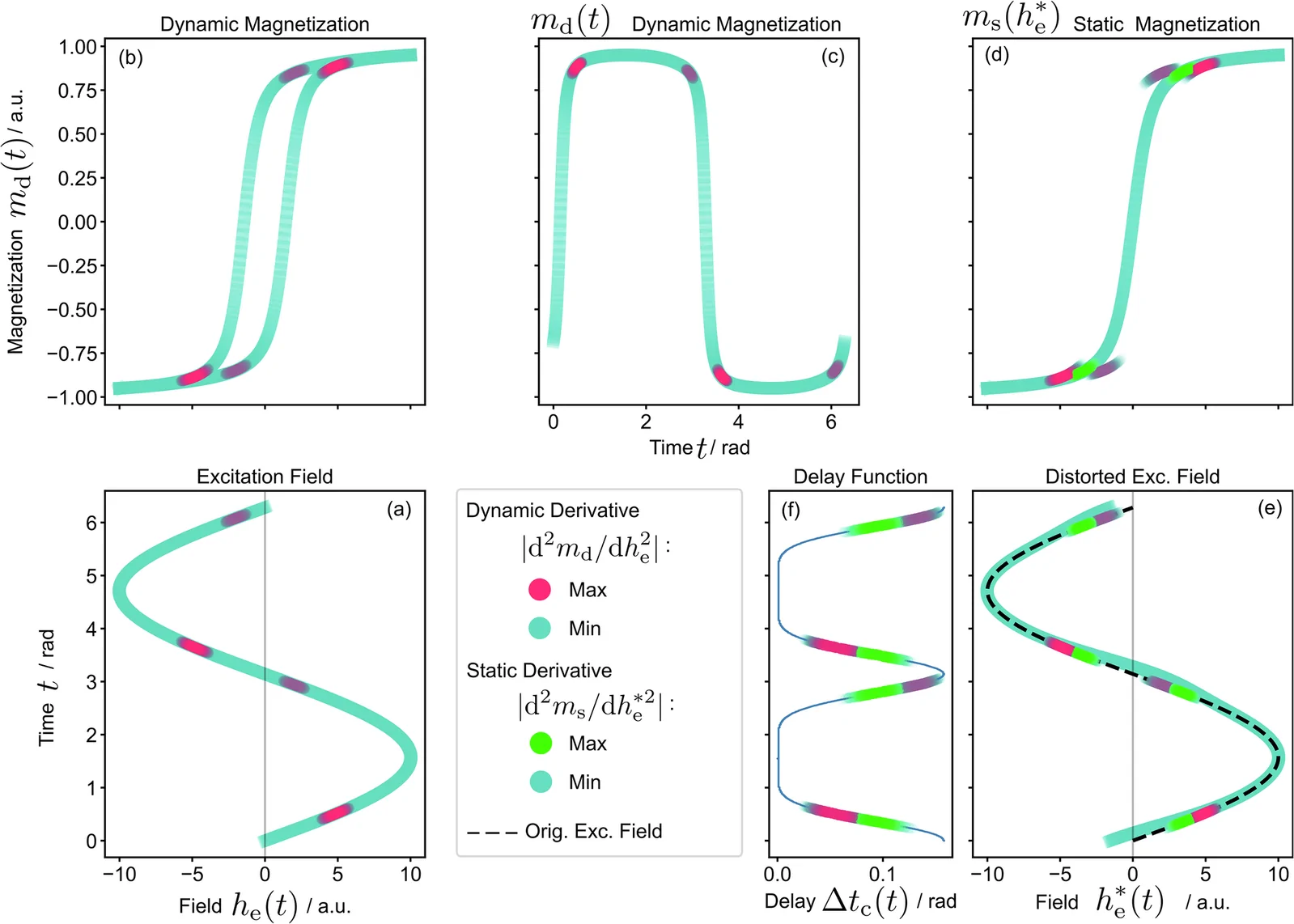
MNP magnetization trajectory evolving in time. **a** Actual single-frequency excitation field ${h}_{{\rm{e}}}\left(t\right),$ **b** Actual dynamic magnetization response ${m}_{{\rm{d}}}(t)$. The red gradient points show the curvature of the dynamic M-H trajectory, **c** Magnetization response ${m}_{{\rm{d}}}(t)$ in time domain, **d** Static magnetization curve ${m}_{{\rm{s}}}\left(h\right)$. The green gradient shows the curvature of static magnetization response. **e** Predistorted version of excitation field ${h}_{{\rm{e}}}^{* }\left(t\right)={h}_{{\rm{e}}}\left(t-\triangle {t}_{{\rm{c}}}\left(t\right)\right)$ that passing through the static magnetization curve ${m}_{{\rm{s}}}\left(h\right)$ gives dynamic magnetization signal ${m}_{{\rm{d}}}(t)$. **f** The delay function $\triangle {t}_{{\rm{c}}}\left(t\right)$ that implements the predistortion of the excitation field.

Figure 3 assists in understanding these effects of amplitude-driven phase modulation on the spectral symmetries of ${m}_{d}(t)$. Consider a single-frequency excitation field ${h}_{{\rm{e}}}\left(t\right)$ as shown in Fig. 3a. This excitation field causes a dynamic magnetization response ${m}_{{\rm{d}}}\left(t\right)$ shown in time domain in Fig. 3c. On the M-H plane, the hysteresis loop is formed due to dynamic losses (Fig. 3b). The red color gradient denotes where the absolute value of the second derivative ${\partial }^{2}{m}_{{\rm{d}}}\left(t\right)/{\partial {h}_{{\rm{e}}}\left(t\right)}^{2}$ is maximal, i.e., curvature is strong. Note that the rate-dependent origin of the nonlinearity can cause asymmetry in the curvature on the ascending and descending paths, which is highlighted by the color difference. The adiabatic model can be extended to account for the effects of the dynamic nonlinearity.

Let us assume that the dynamic magnetization on the M-H plane is replaced with its static counterpart, a static magnetization curve ${m}_{{\rm{s}}}\left({h}_{e}^{* }\right)$ (Fig. 3d), while keeping the dynamic response ${m}_{d}(t)$ unaltered. A similar didactive aid was used by Tumanski^52^, but with a replacement of the output magnetization with a pure harmonic signal. The green gradient shows the peaks of the static magnetization curvature ${\partial }^{2}{m}_{{\rm{s}}}\left({h}_{e}^{* }\right)/{\partial {h}_{e}^{* }}^{2}$. Then, the original excitation field ${h}_{{\rm{e}}}\left(t\right)$ should be replaced with its distorted version ${h}_{e}^{* }\left(t\right)$ such that,

13 $${m}_{{\rm{d}}}\left(t\right)={m}_{{\rm{s}}}\left({h}_{e}^{* }\left(t\right)\right)={m}_{{\rm{s}}}\left({h}_{{\rm{e}}}\left(t-\triangle {t}_{{\rm{c}}}\left(t\right)\right)\right)$$

where $\triangle {t}_{{\rm{c}}}(t)$ is the function that denotes time delay.

We assume that the function $\triangle {t}_{{\rm{c}}}\left(t\right)$ is positive-definite based on the causality principle. Due to the periodicity of the excitation field, $\triangle {t}_{{\rm{c}}}\left(t\right)$ can, in turn, be decomposed into a Fourier series as follows,

14 $$\triangle {t}_{{\rm{c}}}\left(t\right)=\mathop{\sum }\limits_{n=1}^{{L}_{{\rm{td}}}}{a}_{n}^{{\rm{td}}}\cdot \sin \left(2\pi nt{f}_{{\rm{L}}}+{\phi }_{n}^{{\rm{td}}}\right)$$

In a first-order approximation, the phase terms ${\phi }_{n}^{{\rm{td}}}$ are dominated by the low-frequency excitation component, such that ${\phi }_{n}^{{\rm{td}}}\approx n{\varphi }_{{\rm{L}}}$ This approximation holds as the low-frequency component dominates the amplitude of EF (cf. Eq. (2)). The zeroth term from series (14) is excluded as it is already captured in the phase terms ${\varphi }_{{\rm{H}}}$ and ${\varphi }_{{\rm{L}}}$. The dynamic magnetic response of an MNP ensemble, incorporating excitation field phase acquisition and field-dependent relaxation dynamics in the most general case (cf. Fig. 1, A6), can then be written as:

15 $${m}_{{\rm{d}}}\left(t\right)=	\mathop{\sum }\limits_{l=1}^{{L}_{{\rm{sine}}}}{c}_{l}^{{\rm{ms}}}\cdot {\rm{Im}}\left({e}^{{\rm{i}}l\pi \frac{{h}_{{\rm{DC}}}}{{h}_{\max }}}\right.\mathop{\sum }\limits_{n=-\infty }^{\infty }\mathop{\sum }\limits_{m=-\infty }^{\infty }\,\\ 	 \cdot {{\rm{i}}}^{n+m}{J}_{n}\left(l\pi \frac{{h}_{{\rm{H}}}}{{h}_{\max }}\right){J}_{m}\left(l\pi \frac{{h}_{{\rm{L}}}}{{h}_{\max }}\right){e}^{{\rm{i}}\left(n\left(2\pi {f}_{{\rm{H}}}t+{\varphi }_{{\rm{H}}}\right)+m\left(2\pi {f}_{{\rm{L}}}t+{\varphi }_{{\rm{L}}}\right)\right)}\\ 	 \times \left.{\prod }_{p=1}^{{L}_{{\rm{td}}}}\mathop{\sum }\limits_{q=-\infty }^{\infty }{J}_{q}\left(\left(n{f}_{{\rm{H}}}+m{f}_{{\rm{L}}}\right)2\pi {a}_{p}^{{\rm{td}}}\right){e}^{{\rm{i}}q\left(p{f}_{{\rm{L}}}2\pi t+{\phi }_{p}^{{\rm{td}}}+\pi /2\right)}\right)$$

The consequences of amplitude-driven phase modulation on the spectral symmetries become evident: The Bessel function ${J}_{q}\left(\ldots \right)$ acts like a modulation kernel by (a) reducing the amplitude of the spectral term $n{f}_{{\rm{H}}}+m{f}_{{\rm{L}}}$ at q=0 and (b) asymmetrically distorting neighboring harmonic terms ($q\,\ne \,0$), due to its odd symmetry relative to its order q. Conceptually, if the spectrum is imagined as a discrete signal, the modulation acts like a ${L}_{{\rm{td}}}$-fold application of high-pass filters, each with ever-increasing tap spacing: For instance, $2{f}_{{\rm{L}}}$ component in $\triangle {t}_{{\rm{c}}}\left({h}_{{\rm{e}}}\left(t\right)\right)$ affects each neighboring harmonic, $4{f}_{{\rm{L}}}$ component affects each second one and so on. Applying this filter disrupts the spectral symmetries predicted by the equilibrium model: ${\rm{arg}}({A}_{m,p})\,\ne \,m{\varphi }_{{\rm{H}}}+p{\varphi }_{{\rm{L}}}$. These symmetry violations are localized around the carrier frequency, making them experimentally distinguishable and diagnostically valuable for distinguishing MNP systems by their individual non-equilibrium dynamics.

### Samples of magnetic nanoparticles

Eight samples of commercial MNPs were used in experiments: seven iron oxide MNPs, two of which were from Micromod Partikeltechnologie GmbH (Rostock, Germany), (1: “Syn-D 50 nm”) 104-00-501 Synomag-D 50 nm 25 mg/ml Lot.: 06119104-05; (2: “Syn-D 70 nm”) 104-00-701 Synomag-D 70 nm 20 mg/ml Lot.: 37022104-01. Five other types are provided by Ocean Nanotech (San Diego, CA, USA) are supplied with the same concentration of 5 mg/ml: (1: “ON 30 nm”) SPA-30-02 30 nm Lot.: A0223SPA; (2: “ON 25 nm”) SPA-25-02 25 nm Lot.: J0420SPA; (3: “ON 20 nm”) SPA-20-02 20 nm Lot.: F1521ASPA; (4: “ON 15 nm”) SPA-15-02 15 nm Lot.: 111515SPA; (5: “ON 10 nm”) SPA-10-02 10 nm Lot.: 20266SPA. One sample of cobalt-ferrite core particles was also from Micromod: “Co-Fe” 124-00-501 CoFe2O4-Dextran 25 mg/ml Lot.: 18424124-02. As a magnetically linear reference substance, Dysprosium Oxide (III) Lot.: 289264 (Sigma-Aldrich) was employed.

To prevent any interaction between MNPs and the sample containers, all glassware was coated with the siliconizing agent SL2-25ML Sigmacote® (Merck KGaA, Darmstadt, Germany). For static magnetization curve measurements, each MNP type was placed into 10 cm-long borosilicate glass (Duran®) capillaries with an outer diameter of 4 mm and a wall thickness of 0.8 mm, containing 1−2 µL of volume. The droplet was delivered directly to the middle of the capillary using a quartz capillary with a syringe nozzle (Lot.: 4018136, Hilgenberg GmbH, Malsfeld, Germany) measuring 70 mm in length, 1 mm in outer diameter, and 0.1 mm in wall thickness. The resulting sample volume was controlled optically post factum. The capillary was vacuum-sealed at both ends, preventing evaporation and the movement of the sample droplet.

For ac-susceptometry measurements, each MNP type was placed into a glass container made from a 5-cm-long borosilicate (Duran®) glass capillary with an outer diameter of 4 mm, a wall thickness of 0.8 mm, and an inflated vesicle chamber at the end. The chamber volume is approximately 20 µL. MNP colloid was placed using the same kind of injector capillary as in the previous case. In the end, the capillary was vacuum-sealed.

For Frequency Mixing Magnetic Detection measurements, MNP colloids were placed into 2 ml beaded-rim test tubes (FIOLAX® 8 × 70 mm, Duran Wheaton Kimble). For each type of MNP sample, a corresponding sample with blocked Brownian relaxation was prepared by adding 100 µL of gypsum (Lot: 237132, Sigma-Aldrich, Germany) and homogenizing the sample.

As a physical model of a medium with varying viscosity, a glucose syrup was prepared using D-(+)-Glucose (CAS: 50-99-7) Lot.: BCBL6930V (Honeywell, Fluka Analytical). A saturated syrup was prepared by mixing glucose with water in a mass proportion of 1:2. An eight-point series of MNP samples with varying viscosity was prepared, where the stock particle colloid was taken in a volume of 20 µL. The remaining 80 µL were filled with diluted syrup in proportions ranging from 80:0 to 0:80. Samples were then prepared for FMMD measurements. The following MNP types were used for varying viscosity experiments: “ON 30 nm”, “ON 25 nm”, “ON 20 nm”, and “Co-Fe”. “Co-Fe” was additionally prepared for AC susceptometry measurements as described above.

For empirical estimation of the magnetic moment resolution, a mixture series of samples, “Syn-D 50 nm” and “Syn-D 70 nm,” was prepared. The total sample volume was kept constant at 50 µL, and the proportions were varied in a series: 80:20, 50:50, 20:80.

### Setup of intermodulation spectral term measurement

**Fig. 4:**
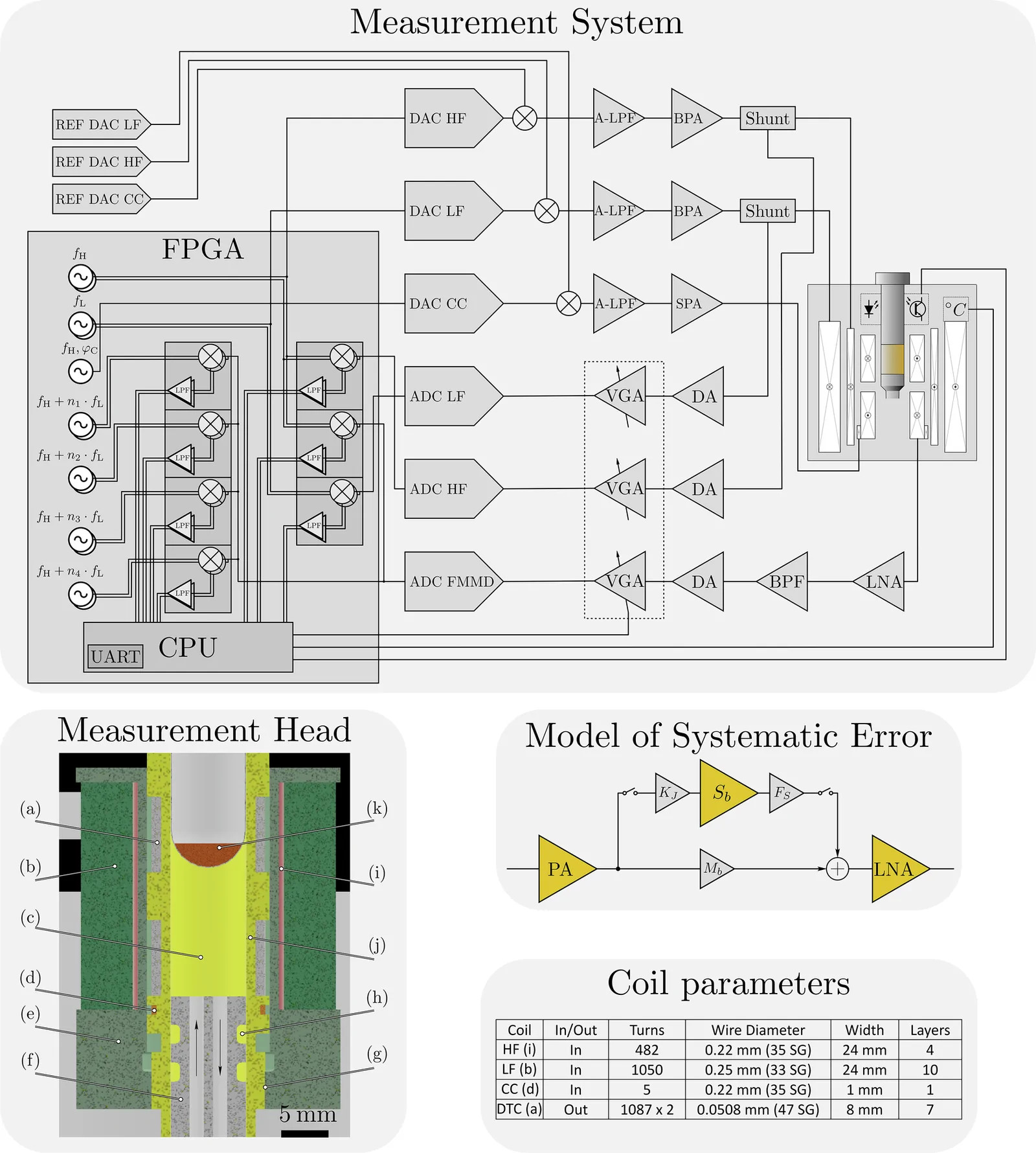
Measurement system specifications. Top: Device internal architecture. FPGA carries three synthesis and seven demodulation blocks. The synthesis circuit uses three DACs that scale the voltage from the reference REF DACs. The excitation output is fed through antialiasing filters to bridged power amplifiers (BPAs). The output currents are monitored using current shunt circuits. The correction channel (CC) is used to actively balance the system. Its output is power-amplified with a Single-sided Power Amplifier (SPA) and applied to the reference part of the DTC. The compensation coil is placed on the distal part of the reference part of the DTC to reduce the influence on the sample. Bottom left: Measurement head cross-section view. (a) Detection Coil (DTC); (b) Low-Frequency Excitation Coil; (c) Space for heat-exchanging liquid; (d) Compensation Coil; (e) Excitation Coil former; (f) Heat exchanger plug. Arrows show the direction of the fluid. (g) Thread for coarse adjustment; (h) Sealing rings (not shown); (i) High-Frequency Excitation coil; (j) DTC former; (k) Sample. Table: Parameters of the coils in the measurement head. Bottom right: Static error propagation in the system caused by self-nonlinearities of the setup. yellow: Nonlinear blocks, gray: Linear blocks, PA: power amplifier, LNA: Low-noise amplifier, ${S}_{b}$: Sample, ${M}_{b}$: Imbalance of measurement head, ${K}_{j}$: Inhomogeneity of the EF, ${F}_{S}$: inhomogeneous coupling of sample’s magnetization to DTC.

Measurement of intermodulation terms (IMTs) was conducted using a custom-built integrated Field-Programmable Gate Array (FPGA)-based device, whose architecture is shown in Fig. 4. The FPGA firmware is similar to that of a system designed for IMT measurement in multifrequency atomic force microscopy, described by Tholén^53^. The excitation frequencies of 10 Hz for low frequency (LF, ${f}_{{\rm{L}}}$) and 40.5 kHz for high frequency (HF, ${f}_{{\rm{H}}}$) are synthesized inside an FPGA running with system clock of 48 MHz using numerically controlled oscillators (NCOs) and converted into analog signals by 10-bit Multiplying Digital-to-Analog Converters (DACs) (“DAC HF”, “DAC LF”, “DAC CC”) at a 1 MHz sampling rate. Amplitude of the excitation signals can be set with 16-bit precision using 3-channel reference DAC (“REF DAC HF”, “REF DAC LF”, and “REF DAC CC” in Fig. 4). Each NCO provides in-phase and quadrature signals simultaneously with a rate of 48 MHz, with 10-bit amplitude precision and 48-bit phase register length, truncated to 12-bit internally. One of the NCOs allows phase adjustment (${\varphi }_{{\rm{C}}}$) and is employed for active interference cancellation through the correction channel (CC). All excitation signals are fed through antialiasing second-order low-pass filters (“A-LPF”) with a cut-off frequency of 80 kHz.

The end of the excitation chain connects excitation signals to bridged power amplifiers (“BPA”) via differential lines to reduce even-order distortions. Two-fold stacked integrated power amplifiers (BUF643, Burr-Brown) are used to achieve currents up to 0.5 A for the generation of the excitation field components reaching amplitudes up to ${h}_{{\rm{L}}}\le 30\,{\rm{mT}}$ and ${h}_{{\rm{H}}}\le 1.6\,{\rm{mT}}$ for LF and HF fields, respectively. A single-sided power amplifier (“SPA”) serves the CC channel, developing currents up to $250\,{\rm{mA @}}\,{f}_{{\rm{H}}}$.

The current measurement circuit, “Shunt,” allows active tracking of the HF and LF fields applied to the sample via synchronous demodulation. The voltage proportional to the current is amplified by the differential amplifier “DA” and amplitude-adjusted to meet the requirements of the dynamic range ($\pm 2.048\,{\rm{V}}$) of the corresponding ADC using the variable gain amplifier “VGA”.

The voltage induced in the detection coil (DTC) is amplified with a low-noise differential amplifier (LNA) with a fixed differential gain of 72, followed by a second-order band-pass filter “BPF” tuned to HF with a bandwidth of 1 kHz. Current signals (“ADC HF” and “ADC LF” in Fig. 4) are digitized synchronously with the voltage induced (“ADC FMMD”) in the DTC with 24-bit Sigma-Delta ADCs at a 24 MHz sampling rate and at 187.5 kHz output data rate. Inside the FPGA, the IMTs are demodulated in zero-intermediate-frequency mode and dispatched via a serial interface.

### Measurement head

The measurement head (MH), a passive air-core transformer with three inputs and one output coil, is the central element of the FMMD measurement system whose geometrical parameters and cross-section are given in Fig. 4. The input (excitation) coils include an LF coil (b), an HF coil (i), and a CC coil (d). The output coil, detection coil (DTC), is wound in the form of a first-order gradiometer (a). The MH is a modification of earlier systems^54^. Coil formers in MH are made from PEEK, a plastic with moderate thermal expansion^55^. For the mechanical balancing of the DTC gradiometer, the excitation coil former (e) can be rotated relative to the DTC former (j). The sample (k) is placed in a glass test tube in the center of one part of the DTC gradiometer (a). The CC coil (d) is wound near the DTC’s reference part to achieve active balancing without affecting the field in the sample area. An insert (f) is connected to the sample below, allowing (k) to be washed with water, which fills the space under the test tube (c). The space under the test tube is sealed with a rubber ring (h, not shown). From above, the sample temperature is monitored by an infrared sensor (MLX90615, Melexis, NV) (not shown), which is calibrated to the water surface. Together with the water-cooling system, it forms a feedback loop and can regulate the sample temperature with 0.5 °C precision.

### Determining the systematic error of FMMD measurement

Self-imposed nonlinearities of the measurement setup fundamentally limit the measurement of IMTs produced by MNP colloids. Unlike the case of an RF receiver, where self-nonlinearities are dominated by the last cascade in the chain, in FMMD measurement systems, the level of self-distortions before the sample is also essential. The scheme at the bottom of Fig. 4 illustrates the origin of the systematic error in FMMD measurement. The gray triangles denote linear effects not contributing to IMTs, such as EF exposure at the sample space $({K}_{j})$ and sample coupling to DTC $\left({F}_{S}\right)$. All active components (yellow triangles) of the setup contribute to IMTs to some extent. The switches symbolize the presence or absence of the sample (${S}_{b}$). It is inevitable that the power amplifier (PA) distorts EF before it is applied to the sample. A pure linear sample can lead to changes in IMTs and loss of specificity by bypassing the IMTs of PA to the Low-noise amplifier (LNA). The LNA, in turn, causes further distortion at the final measurement stage. Self-nonlinearities of the measurement system appear as permanently present nonlinear MNPs with non-physical properties, e.g., negative concentrations, that can be assessed through a background measurement and whose contribution scales with the unbalancing capability of the actual MNP sample. Therefore, we determine the limit of detection (LoD) not by the intersection of the noise floor with the calibration curve (signal amplitude vs. concentration), which would be an overestimation of LoD, but rather by the point at which the calibration curve deviates from linearity ($|{A}_{1,p}|\propto {c}_{s}$ is not fulfilled anymore). These limitations are estimated from (a) dilution series experiments conducted for all types of MNPs and (b) by measurement of a linear sample of Dysprosium Oxide (III). The latter allows an estimate of self-nonlinearities in terms of a non-physical magnetic moment distribution, which can be extrapolated from the fundamental harmonic contribution. Linear terms ${K}_{j}$ and ${F}_{S}$ denote the spatial inhomogeneities of the EF and the non-uniform magnetic coupling between the sample and the DTC. Owing to the sample’s finite dimensions, different regions experience deviating EF amplitudes and contribute unequally to the detected signal. Inhomogeneity of EF ${K}_{j}$ was analyzed numerically using FEMM package. The coupling coefficient ${F}_{S}$ was obtained experimentally by a calibration procedure relative to the static magnetization curve measurement described later.

### Active cancellation of interference

Induced HF-term interference in DTC reduces the accuracy of IMT digitization by occupying the ADC’s dynamic range. The use of a first-order gradiometer in DTC only partially compensates for this interference. To alleviate this problem, passive notch filters^56^ and actively coupled filter systems^57^ are employed in MPI. Still, they are not applicable for FMMD due to the dense localization of IMTs in the frequency domain. The interference might originate not only from inductive coupling between HF and DTC, but also (a) from capacitive coupling between coils in the pF range^58^, and (b) via a shared power supply.

Feeding a signal directly into the LNA input via CC can reduce interference regardless of its origin. The active canceling out of the interference can be achieved in three steps: (a) estimation of the MH imbalance by background measurement at $|{A}_{1,0}|$, (b) evaluation of active compensability by applying a field with CC while the HF coil is switched off. If necessary, HF is reduced in amplitude to allow active balancing. Finally, HF is switched on, and (c) iterative optimization takes place. Tiny offsets of the correction field along in-phase and quadrature directions of CC are added, and the optimal set of compensation parameters can be estimated using the least-squares algorithm to minimize $|{A}_{1,0}|$. Once the next compensation parameters are set, the gain is adjusted to increase the system’s sensitivity. Iterations continue until the maximum gain is reached.

### Measurement of intermodulation terms

The measurement of intermodulation terms (IMTs) ${A}_{1,m}\left({h}_{i}^{{{\rm{L}}}}\right)$ is conducted under a logarithmically varying LF field amplitude ${h}_{i}^{{{\rm{L}}}}$, which we will refer to as LF-scan. With the measurement system described above, a complex signal was measured in eight parallel channels. Five channels are used for IMTs ${A}_{1,m}\left({h}_{i}^{{{\rm{L}}}}\right),\,m\in \left\{0,2,4,6,8\right\}\,{S}_{m}({h}_{i}^{{{\rm{L}}}},{t}_{j})$. Two channels ${C}_{l}({h}_{i}^{{{\rm{L}}}},{t}_{j})$ are used to synchronously track the currents of the HF and LF coils at their respective frequencies. The last channel contains the timestamps of data acquisition with 1 µs precision. 40 LF-amplitude points ${h}_{i}^{{{\rm{L}}}}$ are distributed logarithmically in the range between ${h}_{\min }^{{\rm{FMMD}}}=0.5\,{\rm{mT}}/{\mu }_{0}$ and ${h}_{\max }^{{\rm{FMMD}}}=26\,{\rm{mT}}/{\mu }_{0}$. At each LF-field step ${h}_{i}^{{{\rm{L}}}}$, ${T}_{m}=100$ time points are recorded. Measurement of a single point of an LF-scan lasts 4 s for filter saturation and around 0.6 s for data acquisition of 100 points. To control the long-term drift of the sample, first, odd-indexed points were recorded ${h}_{1}^{{{\rm{L}}}},{h}_{3}^{{{\rm{L}}}},{h}_{5}^{{{\rm{L}}}}$,…, and then even-indexed points in descending order, $\ldots {h}_{6}^{{{\rm{L}}}},{h}_{4}^{{{\rm{L}}}},{h}_{2}^{{{\rm{L}}}}$.

### Evaluation of measurement stability

Synchronous measurement is conducted reliably if (a) the power of the spectral line exceeds the noise floor and (b) the width of the spectral line determined by phase noise does not exceed the width of the output filter. The reliability of the measurement vector ${\vec{S}}_{m}\left({h}_{i}^{{{\rm{L}}}}\right)$ can be qualified using two metrics: a) short-term drift of the FMMD amplitude in the time domain and b) stability of the phase during the measurement. The drift was evaluated by conducting a linear regression between $|{S}_{m}({h}_{i}^{{{\rm{L}}}},{t}_{j})|$, and corresponding timestamps $\tau ({h}_{i}^{{{\rm{L}}}},{t}_{j})$. The regression slope $\left[{V}_{{\rm{FMMD}}}/{\rm{s}}\right]$ demonstrates how fast the amplitude changes over time during the measurement. The phase stability was evaluated by calculating the average phase during the measurement ${\bar{\theta }}_{m,i}={\sum}_{j}{\rm{arg}}({S}_{m}({h}_{i}^{{{\rm{L}}}},{t}_{j}))/{T}_{m}$, and identifying the SNR parameter s from the Rice-Nakagami distribution ${\rho }_{\theta }\left(\theta {|s}\right)$ using the maximum likelihood estimator for each element of the measurement vector^59^,

16 $${{\rm{SNR}}}_{m}\left({h}_{i}^{{{\rm{L}}}}\right)=	\,{{\rm{arg}}\max }_{s}\mathop{\sum }\limits_{j=1}^{{T}_{m}}\log \left({\rho }_{\theta }\left({\rm{arg}}\left({S}_{m}\left({h}_{i}^{{{\rm{L}}}},{t}_{j}\right)\right)-{{\theta }}_{m,i}|s\right)\right)\\ {\rho }_{\theta }\left(\theta |s\right)=	\frac{1}{2\pi }{e}^{-s}\left(1+\sqrt{\pi s}{e}^{s{\cos }^{2}\theta }\cos \theta \left(1+{\rm{erf}}\left(\sqrt{s}\cdot \cos \theta \right)\right)\right)$$

This distribution describes the centered phase θ($\langle \bar{\theta }\rangle =0$) of a synchronously demodulated signal with amplitude A under the presence of narrow-band white noise with power N, where $s={A}^{2}/2N$. This strategy allows identifying cases where reliable synchronous measurement is infeasible, for instance, when a sample has a high but unstable amplitude due to sedimentation.

### Background cancellation

At the next stage, each measurement vector is averaged over measurement time T,

17 $$\begin{array}{c}{\bar{S}}_{m}\left({h}_{i}^{{{\rm{L}}}}\right)=\frac{1}{{T}_{m}}\mathop{\sum }\limits_{j=1}^{{T}_{m}}{S}_{m}\left({h}_{i}^{{{\rm{L}}}},{t}_{j}\right)\\ {\bar{C}}_{l}\left({h}_{i}^{{{\rm{L}}}}\right)=\frac{1}{{T}_{m}}\mathop{\sum }\limits_{j=1}^{{T}_{m}}{C}_{l}\left({h}_{i}^{{{\rm{L}}}},{t}_{j}\right)\end{array}$$

Measurement vectors recorded without a sample are denoted by a “bg” superscript: ${\bar{S}}_{m}^{{\rm{bg}}}\left({h}_{i}^{{{\rm{L}}}}\right)$ and ${\bar{C}}_{l}^{{\rm{bg}}}\left({h}_{i}^{{{\rm{L}}}}\right)$. Using the background signal, cubic interpolators ${{{\mathscr{I}}}}_{m}$ are constructed for each FMMD channel as a function of LF-amplitudes measured in the current channel,

18 $${\bar{S}}_{m}^{{\rm{bg}}}\left({h}_{i}^{{{\rm{L}}}}\right)={{{\mathscr{I}}}}_{m}\left(\left|{\bar{C}}_{2}^{{\rm{bg}}}\left({h}_{i}^{{{\rm{L}}}}\right)\right|\right)$$

These interpolators are used for obtaining the background-subtracted ${\bar{S}}_{m}^{{\rm{corr}}}\left({h}_{i}^{{{\rm{L}}}}\right)$ version, accounting for the actual LF field applied during the measurement.

19 $${\bar{S}}_{m}^{{\rm{corr}}}\left({h}_{i}^{{{\rm{L}}}}\right)={\bar{S}}_{m}\left({h}_{i}^{{{\rm{L}}}}\right)-{{{\mathscr{I}}}}_{m}\left(\left|{\bar{C}}_{2}\left({h}_{i}^{{{\rm{L}}}}\right)\right|\right)$$

According to the properties of the polynomial model, the deviations recorded in ${\bar{C}}_{1}\left({h}_{i}^{{{\rm{L}}}}\right)$ proportionally affect all intermodulation terms in the working band around HF.

### Evaluation of long-term drift

Long-term drift is evaluated by comparison of the linear deviation between even and odd points using,

20 $${\delta }_{m}\left({h}_{2i}^{{{\rm{L}}}}\right)={\bar{S}}_{m}^{{\rm{corr}}}\left({h}_{2i}^{{{\rm{L}}}}\right)-\frac{{\bar{S}}_{m}^{{\rm{corr}}}\left({h}_{2i-1}^{{{\rm{L}}}}\right)+{\bar{S}}_{m}^{{\rm{corr}}}\left({h}_{2i+1}^{{{\rm{L}}}}\right)}{2}$$

When long-term drift is absent, the interpolated difference tends to zero ${\delta }_{m}\left({h}_{2i}^{{{\rm{L}}}}\right)\to 0$. The worst-case deviation characterizes the whole measurement, ${D}_{m}={\max }_{i}\left|{\delta }_{m}\left({h}_{i}^{{{\rm{L}}}}\right)\right|/{\max }_{j}|{\bar{S}}_{m}^{{\rm{corr}}}({h}_{j}^{{{\rm{L}}}})|$.

### Obtaining physically accurate phase

For a physically accurate interpretation of FMMD response and comparison to ac-susceptometry, its phase should be obtained relative to the phase of EF. The raw measurement has its phase relative to the demodulator’s phase, which is coherent but may not equal the actual EF’s phase. The latter obtains delays through the synthesizers, DACs, the power amplifier, and, finally, the coils in the measurement head. Synchronous monitoring of currents in EF coils enables the on-the-fly measurement of the amplitudes and phases of EF field components, right before the sample. The magnetization signal, in turn, acquires phase delays as it passes through the LNA and demodulators (instrumental phase delay).

Subtracting a background from a measurement signal is necessary but not sufficient for obtaining a physically accurate phase delay. Instead, it can be estimated by exploiting the properties of the Wiener model and the fact that MNP colloid is a phase-insensitive system: the work of the system throughout the thermodynamic cycle remains invariant to the changes of the EF component’s phases (${\varphi }_{{\rm{L}}}$ and ${\varphi }_{{\rm{H}}}$). In adiabatic mode, the phase delay is absent, hence ${\rm{arg}}({A}_{1,m})=0$ holds. In general, a dependency ${\rm{arg}}({A}_{1,m})=f({\varphi }_{{\rm{H}}},{\varphi }_{{\rm{L}}})$ is assumed and is considered as a deviation from a case of a simpler nonlinear model, a Wiener model, when ${\rm{arg}}({A}_{1,m})={\varphi }_{{\rm{H}}}+m{\varphi }_{{\rm{L}}}$. In other words, Wiener model provides physically meaningful directions for real and imaginary projections for each IMT. Thus, the algorithm for finding the physically accurate phase of the FMMD response reduces to identifying such a pair $({\varphi }_{{\rm{H}}},{\varphi }_{{\rm{L}}})$ that for N samples for which LF-scans ${A}_{1,m}({h}_{i}^{{\rm{L}}})$ consisting of M field points for five IMTs ${f}_{1}+m{f}_{2},m\in S=\left\{-4,-2,0,2,4\right\}$, the minimization of the mutual phase differences between all corresponding measured points is achieved,

21 $${\min }_{{\theta }_{1}{\theta }_{2}}\mathop{\prod }\limits_{i=1}^{N}\mathop{\sum }\limits_{{j}_{1}\in S}{\sum}_{{j}_{2}\in S/\left\{{j}_{1}\right\}}\mathop{\sum }\limits_{k=1}^{M}\left|{\rm{arg}}\left({A}_{1,{j}_{1}}\left({h}_{i}^{{{\rm{L}}}}\right)\,{e}^{{\rm{j}}\left({\theta }_{1}+{j}_{1}{\theta }_{2}\right)}\right)-{\rm{arg}}\left({A}_{1,{j}_{2}}\left({h}_{i}^{{{\rm{L}}}}\right){e}^{{\rm{j}}\left({\theta }_{1}+{j}_{2}{\theta }_{2}\right)}\right)\right|$$

For obtaining the pair $\left({\varphi }_{{\rm{H}}},{\varphi }_{{\rm{L}}}\right)$ the four largest sizes of Ocean Nanotech MNP samples from 30 nm to 15 nm in liquid and gypsum were considered simultaneously.

### Evaluation of linearity of higher order terms

Phase property ${\rm{arg}}({A}_{\cdot ,n})\propto n$ is quantitatively analyzed using linear regression at each point of the LF-scan. Only points exceeding the background by one order of magnitude are considered $|{A}_{1,8}| > 10|{A}_{1,8}^{{\rm{floor}}}|$. For each LF amplitude value ${h}_{i}^{{{\rm{L}}}}$, regression parameters were calculated: (a) Pearson’s correlation, (b) *p*-value, and (c) slope of the phase “rad/order”.

### Regularized reconstruction

To reconstruct the distributions of relaxation times (from single-frequency dynamic magnetization using ACS) and magnetic moments (from static magnetization using MPMS and dual-frequency dynamic magnetization using FMMD), we apply a unified approach to solve the inverse problem with regularization, without any a priori assumptions on the shape of the distribution (e.g., log-normal). Instead, the histogram is directly reconstructed. In this way, assumptions such as mono- or polydispersity are ultimately avoided. The range of parameters this approach can reconstruct with trustworthiness is determined in advance based on the signal’s geometrical features. Consider a bell-shaped curve displaying a global maximum (e.g., the peak in the imaginary part of ac-susceptometry). One can only precisely determine the peak position if it is observed within the method’s operating range (e.g., 10 to 10,000 Hz for ac-susceptometry in this work). Thus, the reconstruction range covers the operating range, with a slight overlap on both sides, and is extended by a factor of 10. The trustworthy region equals the operating range, and the residual regions at the sides are treated as background that cannot be precisely reconstructed.

A zero-order Tikhonov regularization is applied to stabilize the solution. Regularization of a higher order (e.g., constraining the higher order derivatives) is not used to avoid imposing physically unreasonable constraints on the reconstructed distributions’ smoothness and to exclude the appearance of artifactual peaks. Reconstruction reduces to solving the following matrix equation using the bounded least-squares algorithm,

22 $$\left[\begin{array}{c}{M}^{{\rm{rec}}}\\ \lambda E\end{array}\right]\vec{x}=\left[\begin{array}{c}{\vec{b}}^{{\rm{meas}}}\\ \vec{0}\end{array}\right]$$

where ${\vec{b}}^{{\rm{meas}}}$ is the measured data, $\vec{0}$ is the zero vector, ${M}^{{\rm{rec}}}$ is the reconstruction matrix, E is the unit matrix, and λ is the regularization parameter. The exact form of the matrix and the input vectors vary across measurement methods (ACS, MPMS, FMMD). A standard non-negative least-squares algorithm implemented in BLAS/LAPACK with Python bindings was used to find the solution vector $\vec{x}$.

### Reconstruction of magnetic moment distribution from FMMD

Distribution of magnetic moments, ${\rho }_{m}^{{\rm{FMMD}}}\left({m}_{i}^{{{\rm{FMMD}}}}\right)$ was reconstructed from FMMD measurements in the range $\left[{m}_{\min }^{{\rm{FMMD}}},{m}_{\max }^{{\rm{FMMD}}}\right]$ with logarithmic steps ${m}_{i}^{{{\rm{FMMD}}}}\,={m}_{\min }^{{\rm{FMMD}}}{\left({m}_{\max }^{{\rm{FMMD}}}/{m}_{\min }^{{\rm{FMMD}}}\right)}^{\frac{i-1}{M-1}}$, M=300. The range was selected based on the field range of LF-fields, with the range extended ten times on both sides: ${m}_{\min }^{{\rm{FMMD}}}=0.1\cdot {\alpha }_{{\rm{FM}}}/{h}_{\max }^{{\rm{FMMD}}}=\,3.11\cdot {10}^{-20}\,{\rm{A}}{{\rm{m}}}^{2}$ and ${m}_{\max }^{{\rm{FMMD}}}=10\cdot {\alpha }_{{\rm{FM}}}/{h}_{\min }^{{\rm{FMMD}}}=\,8.09\cdot {10}^{-17}\,{\rm{A}}{{\rm{m}}}^{2}$. Here ${\alpha }_{{\rm{FM}}}=4.04\cdot {10}^{-21}{\rm{A}}{{\rm{m}}}^{2}{{\rm{T}}}^{-1}$ is a proportionality coefficient connecting the curvature peak of the Langevin curve and the magnetic field^19^.

The reconstruction follows formulation (22), defining the reconstruction matrix ${M}^{{rec}}\in {{\mathbb{R}}}^{N\times M}$ filled with corresponding values of LF-field and magnetic moment: ${\left[{M}^{{\rm{rec}}}\right]}_{i,j}=|{\hat{A}}_{1,2}({h}_{i}^{{{\rm{L}}}},{m}_{j}^{{{\rm{FMMD}}}})|/{\max }_{x}|{\hat{A}}_{1,2}(x,{m}_{j}^{{{\rm{FMMD}}}})|$. Measurement vector ${\vec{b}}^{{\rm{meas}}}\in {{\mathbb{C}}}^{N}$ was filled with processed measurements ${b}_{i}^{{meas}}={A}_{1,2}\left({h}_{i}^{{{\rm{L}}}}\right)$. The resulting distribution is obtained by taking the absolute value of the solution $\vec{x}$ ${\rho }_{m}\left({m}_{i}^{{{\rm{FMMD}}}}\right)=\left|{\left[\vec{x}\right]}_{i}\right|$. The regularization parameter ${\lambda }^{{\rm{FMMD}}}$ was selected using the L-curve method^60^, optimized in the range ${\lambda }^{{\rm{FMMD}}}\in \left[{10}^{-3},10\right].$

### Reconstruction of magnetic moment distribution from MPMS

Distribution of magnetic moments ${\rho }_{m}^{{{\rm{MPMS}}}}\left({m}_{i}^{{{\rm{MPMS}}}}\right)$ was reconstructed from MPMS measurements in the range ${m}_{j}^{{{\rm{MPMS}}}}\in \left[{m}_{\min }^{{\rm{MPMS}}},{m}_{\max }^{{\rm{MPMS}}}\right]$ with M=300 logarithmic steps. The range was selected based on the field range of MPMS: ${m}_{\min }^{{\rm{MPMS}}}=0.1\cdot \frac{{\alpha }_{{\rm{FM}}}}{{h}_{\max }^{{\rm{MPMS}}}}=\,8.09\cdot {10}^{-22}\,{\rm{A}}{{\rm{m}}}^{2}$ and ${m}_{\max }^{{\rm{MPMS}}}=10\cdot \frac{{\alpha }_{{\rm{FM}}}}{{h}_{\min }^{{\rm{MPMS}}}}=\,8.09\cdot {10}^{-17}\,{\rm{A}}{{\rm{m}}}^{2}$. The reconstruction follows formulation (22), defining the reconstruction matrix ${M}^{{rec}}\in {{\mathbb{R}}}^{N\times M}$ filled with corresponding static normalized magnetization curves and moment values: ${\left[{M}^{{rec}}\right]}_{i,j}={{\mathcal{L}}}\left({m}_{j}^{{{\rm{MPMS}}}}{\mu }_{0}{h}_{i}^{{\rm{ms}}}/{k}_{B}T\right)$. Measurement vector ${\vec{b}}^{{\rm{meas}}}\in {{\mathbb{R}}}^{N}$ was filled with processed measurements ${b}_{i}^{{meas}}={m}_{{\rm{ms}}}\left({h}_{i}^{{\rm{ms}}}\right)$, as described above. The resulting distribution is obtained by taking the absolute value of the solution $\vec{x}$ : ${\rho }_{m}\left({m}_{i}^{{{\rm{MPMS}}}}\right)={\left[\vec{x}\right]}_{i}$. The regularization parameter ${\lambda }^{{\rm{MPMS}}}$ was selected so to achieve the same Full-Width Half Maximum in terms of magnetic moment distribution as for FMMD reconstruction to achieve comparability between both methods.

### Relaxation time distribution reconstruction from ACS

Distribution of cut-off frequencies ${\rho }_{\omega }\left({\omega }_{i}^{{{\rm{ACS}}}}\right)$ was reconstructed from ACS measurements in the range ${\omega }_{{\rm{i}}}^{{{\rm{ACS}}}}\in \left[{\omega }_{\min }^{{{\rm{ACS}}}},{\omega }_{\max }^{{{\rm{ACS}}}}\right]$ with M=100 logarithmic steps. The range was selected based on the frequency range available for this specific ACS: ${\omega }_{\min }^{{{\rm{ACS}}}}=0.1\cdot 10\,{\rm{Hz}}\cdot 2\pi =1\,{\rm{Hz}}\cdot 2\pi$ and ${\omega }_{\max }^{{{\rm{ACS}}}}=10\cdot {10}^{4}\,{\rm{Hz}}\cdot 2\pi ={10}^{5}\,{\rm{Hz}}\cdot 2\pi$. The following susceptometry model, which includes two cut-off frequencies, was used for fitting the ACS results,

23 $$H(j\omega )=\frac{{a}_{{\rm{N}}}}{1+{\omega }^{2}{\tau }_{{\rm{N}}}^{2}}+j\omega \frac{{a}_{{\rm{N}}}{\tau }_{{\rm{N}}}}{1+{\omega }^{2}{\tau }_{{\rm{N}}}^{2}}+\mathop{\sum }\limits_{i=0}^{N}\left[\frac{{\left({a}_{{\rm{B}}}\right)}_{i}}{1+{\omega }^{2}{\left({\tau }_{{\rm{B}}}^{2}\right)}_{i}}+j\omega \frac{{\left({a}_{{\rm{B}}}\right)}_{i}{\left({\tau }_{{\rm{B}}}\right)}_{i}}{1+{\omega }^{2}{\left({\tau }_{{\rm{B}}}^{2}\right)}_{i}}\right]$$

This model includes one relaxation time ${\tau }_{{\rm{N}}}$ lying outside of the ACS working range, nominally called Néel relaxation, and a set of relaxation times ${\left({\tau }_{{\rm{B}}}\right)}_{i}$ expected within the working range are called Brownian relaxation times. Scaling factors ${a}_{{\rm{N}}}$ and ${\left({a}_{{\rm{B}}}\right)}_{i}$ constitute a vector of unknowns: $\vec{x}=({a}_{{\rm{N}}},{({a}_{{\rm{B}}})}_{0},{({a}_{{\rm{B}}})}_{1},\ldots )\in {{\mathbb{R}}}^{N+1}$. The reconstruction follows formulation (22), defining the reconstruction matrix ${M}^{{\rm{rec}}}\in {{\mathbb{C}}}^{\left(N+1\right)\times M}$:

24 $${\left[{M}^{{rec}}\right]}_{i,1}=	\frac{1}{1-{\rm{i}}2\pi {f}_{{\rm{i}}}^{{{\rm{ACS}}}}{\tau }_{{\rm{N}}}}\,\\ {{\left[{M}^{{rec}}\right]}_{i,j}|}_{j\ne 1}=	\frac{1}{1-{\rm{i}}\frac{2\pi {f}_{{\rm{j}}}^{{{\rm{ACS}}}}}{{\omega }_{{\rm{i}}}^{{{\rm{ACS}}}}}}$$

Measurement vector ${\vec{b}}^{{\rm{meas}}}\in {{\mathbb{C}}}^{N+1}$ was filled with processed measurements ${b}_{i}^{{meas}}=\chi ({f}_{{\rm{j}}}^{{{\rm{ACS}}}})$. The resulting distribution is obtained by taking the absolute value of the solution $\vec{x}$: ${\rho }_{\omega }\left({\omega }_{i}^{{{\rm{ACS}}}}\right)={\left[\vec{x}\right]}_{i},\,i=1\ldots N$. The regularization parameter was selected using the L-curve, optimized in the range $\lambda \in \left[{10}^{-3},10\right].$

### Measurement of dynamic magnetic properties

AC susceptometry responses of all MNP stock sample types plus “Co-Fe” samples with varying media viscosity were measured inductively using a Physical Property Measurement System (Quantum Design), ranging from 10 Hz to 10 kHz in logarithmic steps with 5 points per decade (51 points total). The amplitude of the excitation field was 1 mT. The measurement result was averaged over five measurements. Each susceptibility spectrum was measured at zero DC field and at a bias field of 30 mT, imitating the end point of an FMMD measurement. For the three selected samples, AC susceptometry was measured at 10 points across the same frequency range, with bias fields logarithmically distributed from 0.5 mT to 30 mT in 5 steps.

### Evaluation of asymptotic correspondence between ACS and FMMD

Because dynamic measurement is unavoidable to increase magnetic moment resolution, dynamic effects should be considered. Asymptotically, FMMD measurement is expected to converge to an ac-susceptometry with an offset field as ${f}_{{{\rm{L}}}}$ tends to zero. Thus, a comparison should be conducted between these methods using a physical model in which the dissipation factor is varied in a controlled manner. A model with variable viscosity serves this purpose. As we assume that amplitude effects are dominated by the LF EF component and rate effects by its HF counterpart, the corresponding frequency points of ac-susceptometry are correlated with the IMTs of the FMMD response. The phase and amplitude changes in both methods are compared. A viscosity experiment should reveal how the dissipation factor influences the nonlinear response of FMMD without varying the interaction between MNPs. A coarse response shape comparison is done using characteristic points, such as the field where IMT peaks. A sample for this experiment should be sensitive to viscosity changes, showing changes in the FMMD signal, and at the same time should have a Brownian relaxation time ${\tau }_{B}$ in the working range of the Physical Property Measurement System (PPMS) system, so changes in it can be precisely identified at both LF and HF frequencies. Sensitivity to the changes in viscosity is characterized by ${\alpha }_{{\rm{R}}}$ parameter that is defined as the ratio of the mean change between the LF sample points for the liquid sample and the sample fixed in gypsum, normalized by the maximum signal amplitude of the liquid sample,

25 $${\alpha }_{{\rm{R}}}\left({f}_{{\rm{L}}},{f}_{{\rm{H}}}\right)=\frac{1}{M}\mathop{\sum }\limits_{i=1}^{M}\frac{|{A}_{1,2}^{{\rm{liq}}}\left({h}_{i}^{{{\rm{L}}}}\right)-{A}_{1,2}^{{\rm{gyp}}}\left({h}_{i}^{{{\rm{L}}}}\right)|}{|{A}_{1,2}^{{\rm{liq}}}\left({h}_{i}^{{{\rm{L}}}}\right)|}$$

In case ${\alpha }_{{\rm{R}}}=0$, the particle has a pure Brownian relaxation, and when ${\alpha }_{{\rm{R}}}=1$, is a pure Néelian particle. Note that ${\alpha }_{{\rm{R}}}$ implicitly depends on the field frequencies applied to the particles. The viscosity model was verified in two ways: by linear regression of the estimated relaxation time on viscosity. In the first case, the media viscosity is estimated using an empirical formula, and the viscosity power is compared to 1. In the second case, the proportionality between relaxation time and resulting viscosity was enforced, and the original syrup viscosity was estimated. Finally, the hydrodynamic diameter is estimated from viscosity changes, assuming it remains invariant throughout the measurements. For most extreme viscosity cases, AC susceptibility is measured with varying offset field and compared to the FMMD response in the complex plane.

The asymptotic correspondence is investigated by ac-susceptometry measurements with an additional dc-offset component ${h}_{{\rm{DC}}}\in \left\{0.5,\,1.39,3.87,\,10.8,\,30.0\right\}\,\,{\rm{mT}}$. In each case, the average Brownian relaxation time is estimated using model (23) from a varying part ${\left({\tau }_{{\rm{B}}}\right)}_{i}$. Field dependence of relaxation time ${\tau }_{{{\rm{B}}}}\left({h}_{{\rm{DC}}}\right)$ as a function of offset component ${h}_{{\rm{DC}}}$ is fitted by a curve using the least-squares algorithm, obtaining a pair of parameters $\left({\tau }_{0},\xi \right)$^35^,

26 $${\tau }_{{{\rm{B}}}}\left({h}_{{\rm{DC}}}\right)={\tau }_{0}\xi {h}_{{\rm{DC}}}{{{\mathcal{L}}}}^{{\prime} }\left(\xi {h}_{{\rm{DC}}}\right)/{{\mathcal{L}}}\left(\xi {h}_{{\rm{DC}}}\right)$$

### Measurement of the static magnetization curve

A superconducting magnetometry Magnetic Property Measurement System (Quantum Design, San Diego, USA) was used to measure the static magnetization curve ${m}_{{\rm{ms}}}\left({h}_{i}^{{\rm{ms}}}\right)$ of all types of MNP in liquid colloidal form. The magnetic field points ${h}_{i}^{{\rm{ms}}}$ were varied in geometric progression ${h}_{\pm i}^{{\rm{ms}}}=\pm {h}_{\min }^{{\rm{ms}}}{\left({h}_{\max }^{{\rm{ms}}}/{h}_{\min }^{{\rm{ms}}}\right)}^{\frac{i-1}{N-1}}$, where ${h}_{\min }^{{\rm{ms}}}=0.1\,{\rm{mT}}{\mu }_{0}^{-1}$ and ${h}_{\max }^{{\rm{ms}}}=1\,{\rm{T}}{\mu }_{0}^{-1}$ in the following sequence: first, (a) even-indexed points ${h}_{\pm 2i}^{{\rm{ms}}}$ were measured in descending order, then (b) odd-indexed points ${h}_{\pm \left(2i+1\right)}^{{\rm{ms}}}$ were measured in ascending order. This scheme provides better coverage of the field than the symmetric scheme and allows for drift evaluation. The rate of field change was limited to $|\dot{h}|\le 10\,{\rm{mT}}{{\rm{s}}}^{-1}{\mu }_{0}^{-1}$. The measurements were performed in the Reciprocating Sample Option mode, in which the sample was moved through the input gradiometer at 4 Hz and 4 cm amplitude, making 12 consecutive passes. To improve accuracy, each point was measured three times. The resulting magnetic moment value is obtained in MPMS under the assumption of negligible sample dimensions by fitting a point-spread function to the registered voltage profile.

The data were processed as follows: (a) the curve ${m}_{{\rm{ms}}}\left({h}_{i}^{{\rm{ms}}}\right)$ was centered with respect to zero, then (b) the negative part of the curve was mirrored into the positive half-plane, exploiting the odd symmetry of the curve. Finally, (c) the measurement points ${m}_{{\rm{ms}}}\left({h}_{i}^{{\rm{ms}}}\right)$ corresponding to 10% near maximum ${h}_{i}^{{\rm{ms}}}\ge 0.9\,{\rm{T}}{\mu }_{0}^{-1}$ were used to estimate the parameters of the asymptote of the Langevin function ${m}^{{\rm{ms}}}={m}_{{\rm{sat}}}\cdot \left(1-{h}_{{\rm{chr}}}/h\right)+{\chi }^{\infty }\cdot h$, where ${m}_{{\rm{sat}}}$ is the saturated magnetic moment of the sample, ${h}_{{\rm{chr}}}$ is the characteristic field and ${\chi }^{\infty }$ is the susceptibility of the linear background. The resulting asymptote $f\left(h\right)={\chi }^{\infty }\cdot h$ was subtracted from the magnetization data for the removal of the linear background.

### Calibration of the magnetic moment reconstruction

Calibration coefficients, interconnecting intensity-weighted magnetic moment distributions of FMMD ${\rho }_{{\rm{m}}}^{{\rm{FMMD}}}\left(x\right)$ and of MPMS ${\rho }_{{\rm{m}}}^{{\rm{MPMS}}}\left(x\right)$ with saturated magnetic moment, were obtained using two types of MNPs, specifically “Syn-D 50 nm” and “Syn-D 70 nm”. These samples were selected due to their narrow magnetic moment distributions, compliance with a Wiener model, and full coverage of their distribution by the FMMD range $\left[{m}_{\min }^{{\rm{FMMD}}},{m}_{\max }^{{\rm{FMMD}}}\right]$. Volumes (~3 µL each) were estimated optically for both samples, “Syn-D 50 nm” and “Syn-D 70 nm” prepared for MPMS measurement, and the total magnetic moment ${m}_{{\rm{sat}}}$ was obtained from a static magnetic curve measurement.

27 $${m}_{{\rm{sat}}}={c}_{{\rm{m}}}^{{\rm{MPMS}}}\int\limits^{{m}_{\max }^{{\rm{FMMD}}}}_{{m}_{\min }^{{\rm{FMMD}}}}\frac{{\rho }_{{\rm{m}}}^{{\rm{MPMS}}}\left(x\right)}{x}{dx}\,\gtrsim \,{c}_{{\rm{m}}}^{{\rm{FMMD}}}\int\limits^{{m}_{\max }^{{\rm{FMMD}}}}_{{m}_{\min }^{{\rm{FMMD}}}}\frac{{\rho }_{{\rm{m}}}^{{\rm{FMMD}}}\left(x\right)}{x{\max }_{{h}_{{\rm{L}}}}\left|{\hat{A}}_{1,2}\left({h}_{{\rm{L}}},x\right)\right|}{dx}$$

Calibration coefficients ${c}_{{\rm{m}}}^{{\rm{FMMD}}}$ and ${c}_{{\rm{m}}}^{{\rm{MPMS}}}$ were obtained first using “Syn-D 50 nm” and evaluated afterwards using “Syn-D 70 nm” by comparing the ${m}_{{\rm{sat}}}$ obtained from reconstruction with the one obtained from the MPMS measurement. First, both sample types were sealed in glass capillaries and then measured using MPMS, from which their ${m}_{{\rm{sat}}}$ were identified. Following the reconstruction procedure of MPMS, the distribution ${\rho }_{m}^{{\rm{MPMS}}}\left(x\right)$ with calibration coefficient ${c}_{{\rm{m}}}^{{\rm{MPMS}}}$ was obtained. Subsequently, a capillary with “Syn-D 50 nm” was measured using FMMD; the ${\rho }_{m}^{{\rm{FMMD}}}\left(x\right)$ distribution was reconstructed, integrated and ${c}_{{\rm{m}}}^{{\rm{FMMD}}}$ was extrapolated using (27), accounting for the sample volume. Then the same type, “Syn-D 50 nm”, was measured in the FMMD test tube in a volume of 50 µL, and the coefficient ${c}_{{\rm{m}}}^{{\rm{FMMD}}}$ was obtained again. Finally, calibration is evaluated by repeating the procedure for “Syn-D 70 nm”.

### Distribution comparison

The reconstructed magnetic moment distributions ${\rho }_{{\rm{m}}}^{{\rm{FMMD}}}\left({x|}\lambda \right)$ from FMMD and static magnetization curve measurements ${\rho }_{{\rm{m}}}^{{\rm{MPMS}}}\left({x|}\lambda \right)$ were compared per-quantile and depicted with quantile-quantile (QQ) plots. The relative error that covers 95% of all points was used as a figure of merit for evaluating the similarity between distributions. Linear regression was used to assess QQ-plots and numerically characterize the difference between the distributions.

### Evaluation of the system’s resolution for mixed particle systems

The magnetic moment resolution in ${\rho }_{{\rm{m}}}^{{\rm{FMMD}}}\left(x\right)$ was experimentally estimated using the samples “Syn-D 50 nm,” “Syn-D 70 nm,” and their mixtures. The resolution was evaluated using intensity-weighted distributions ${\rho }_{{\rm{m}}}^{{\rm{FMMD}}}\left({x|}\lambda \right)$ obtained for various regularization parameters λ. At first, the series were measured, and the ratio that gave approximately equal contributions to the FMMD signal was chosen. Then the peak magnetic moments were identified for both samples, ${m}_{0}^{{\rm{SynD}}50}$ and ${m}_{0}^{{\rm{SynD}}70}$. The resolution ${R}_{{\rm{FMMD}}}\left(\lambda \right)$ was calculated as the ratio for various λ for the sample that gave approximately equal contributions of both peaks,

28 $${R}_{{\rm{FMMD}}}\left(\lambda \right)=\frac{{\rho }_{m}^{{\rm{FMMD}}}\left({m}_{0}^{{\rm{SynD}}70}|\lambda \right)+{\rho }_{m}^{{\rm{FMMD}}}\left({m}_{0}^{{\rm{SynD}}50}|\lambda \right)}{2{\rho }_{m}^{{\rm{FMMD}}}\left(\sqrt{{m}_{0}^{{\rm{SynD}}50}{m}_{0}^{{\rm{SynD}}70}}|\lambda \right)}$$

The reconstruction was performed at different values of the regularization parameter λ. The mixture was considered unresolvable if ${R}_{{\rm{FMMD}}}\left(\lambda \right)\le 1$.

## Results

### Measurement system evaluation

Our measurement system (described in “Setup of intermodulation spectral term measurement”) demonstrated maximum achievable ${h}_{{\rm{L}}}$ amplitude equal to $26.1\pm 0.1\,{\rm{mT}}{\mu }_{0}^{-1}{\rm{@}}10\,{\rm{Hz}}$, verified by measurement using a Hall probe (APS11760, Allegro Microsystems). The amplitude for the ${h}_{{\rm{H}}}$ field was $1.6\pm 0.1\,{\rm{mT}}{\mu }_{0}^{-1}{\rm{@}}40.5\,{\rm{kHz}}$, measured using a 5 mm wire loop. Assuming a uniform magnetization, a cylindrical sample with 5 mm diameter and 5 mm height with magnetization of $1\,{\rm{A}}{{\rm{m}}}^{-1}$ (total magnetic moment $9.81\cdot {10}^{-8}\,{\rm{A}}{{\rm{m}}}^{2}$) creates a numerically estimated flux of $2.03\cdot {10}^{-9}\,{\rm{Wb}}$ in the detection coil (DTC). Estimated coupling at $40.5\,{\rm{kHz}}$ equals $1.89\cdot {10}^{-4}{\rm{A}}{{\rm{m}}}^{2}/{{\rm{V}}}_{{\rm{p}}}$ or $2.68\cdot {10}^{-4}{\rm{A}}{{\rm{m}}}^{2}/{{\rm{V}}}_{{\rm{RMS}}}$ in Root-Mean-Square (RMS) terms.

With the help of Spectral Analyzer (SA) HP35670A, the system’s noise measured at the end of Low-Noise Amplifier (LNA) and recalculated to its input equals $27\,{\rm{n}}{{{\rm{V}}}}_{{\rm{RMS}}}/\sqrt{{\rm{Hz}}}$ or $7.37\cdot {10}^{-14}{{\rm{A}}}{{{\rm{m}}}}^{2}/\sqrt{{\rm{Hz}}}$ (recalculated to the sample volume). The SA’s effective noise bandwidth of 51 kHz and LNA gain of 72 were considered in calculations. The LNA’s noise floor remained unchanged when the measurement head was replaced with a 50-ohm shunt. SA’s self-noise floor measured with a 50-ohm shunt was estimated to $0.526\,{\rm{n}}{{{\rm{V}}}}_{{\rm{RMS}}}/\sqrt{{\rm{Hz}}}$. The ratio between SA measurements and the values obtained after demodulation with the integrated device is $2.56\cdot {10}^{-2}\,{{{\rm{V}}}}_{{\rm{FMMD}}}/\left({{{\rm{V}}}}_{{\rm{RMS}}}/\sqrt{{\rm{Hz}}}\right)$. ${{{\rm{V}}}}_{{\rm{FMMD}}}$ is an RMS value, too, and the subscript “FMMD” emphasizes the source. The predicted noise floor for the FPGA setup based on the measurement with SA is $6.912\cdot {10}^{-10}\,{{{\rm{V}}}}_{{\rm{FMMD}}}$. The noise floor of the integrated device obtained from the background measurements equal $2.89\cdot {10}^{-9}\,{{{\rm{V}}}}_{{\rm{FMMD}}}$ or $0.112\,{{\rm{\mu }}}{{{\rm{V}}}}_{{\rm{RMS}}}/\sqrt{{\rm{Hz}}}$ if recalculated at the LNA’s input or $3.02\cdot {10}^{-11}\,{{\rm{A}}}{{{\rm{m}}}}^{2}/\sqrt{{\rm{Hz}}}$ at the sample’s volume.

To increase the dynamic range of the measurement, the active balancing (described in “Active cancellation of interference”) of the system was completed in five iterations, reducing the fundamental harmonic by 79 times down to $91.3\,\mu {{{\rm{V}}}}_{{\rm{RMS}}}/\sqrt{{\rm{Hz}}}$ ($17.6\cdot {10}^{-11}\,{{\rm{A}}}{{{\rm{m}}}}^{2}/\sqrt{{\rm{Hz}}}$). The levels of background intermodulation harmonics were $3.87\,{{\rm{\mu }}}{{{\rm{V}}}}_{{\rm{RMS}}}/\sqrt{{\rm{Hz}}}$ ($0.74\cdot {10}^{-11}{{\rm{A}}}{{{\rm{m}}}}^{2}/\sqrt{{\rm{Hz}}}$) for the second-order harmonic $\left|{A}_{1,1}\right|$ and $1.89\,{{\rm{\mu }}}{{{\rm{V}}}}_{{\rm{RMS}}}/\sqrt{{\rm{Hz}}}$ ($0.364\cdot {10}^{-11}{{\rm{A}}}{{{\rm{m}}}}^{2}/\sqrt{{\rm{Hz}}}$) for the third-order harmonic $\left|{A}_{1,2}\right|$. Evaluation of power-supply coupling by replacing the detection coil with a 50-ohm shunt reveals that the background fundamental harmonic reduces ten times in amplitude.

**Fig. 5:**
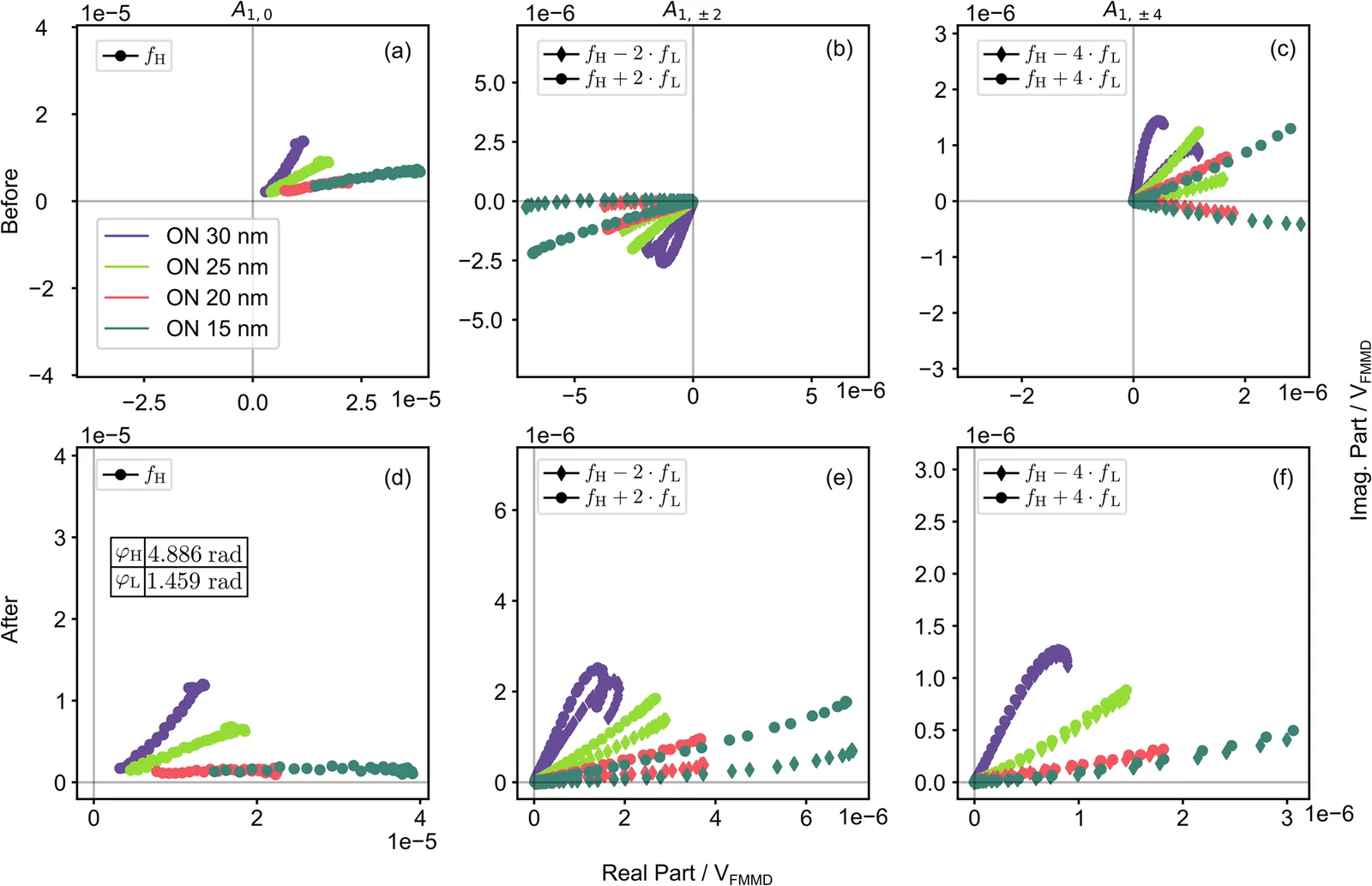
Determination of the phases of the EFs. **a**–**c** Ocean Nanotech samples at IMTs ${A}_{{\mathrm{1,0}}}$, ${A}_{1,\pm 2},{A}_{1,\pm 4}$ before consideration of the EF phases. **d**–**f** After correction of EF phases. Table (**d**) contains identified phase values.

A pair of EF phases $\left({\varphi }_{{\rm{H}}},{\varphi }_{{\rm{L}}}\right)$ required for estimation of physically accurate phase response and further comparison with AC-susceptometry was obtained by modified FMMD measurements (cf. “Obtaining physically accurate phase”) of the four largest MNP samples of Ocean Nanotech “ON (15-30) nm”. Figure 5 shows the raw measurement (top row) and the result after consideration of the obtained phase pair (bottom row).

The phase difference between the corresponding points of all four measurements is minimized. A constant discrepancy between the values at ${A}_{1,\pm 2}$ IMT, 0.160±0.018 rad $({{\rm{N}}}=77;\pm 3{{\rm{\sigma }}})$ is observed, which can be attributed to background asymmetry between ${A}_{1,-2}$, and ${A}_{1,2}$ caused by self-nonlinearities of the device (cf. “Determining the systematic error of FMMD measurement”) For ${A}_{1,\pm 4}$ harmonics the difference is $0.028\pm 0.024\,{{\rm{rad}}}\,({{\rm{N}}}=47;\pm 3{{\rm{\sigma }}})$.

### MNP samples evaluation

**Fig. 6:**
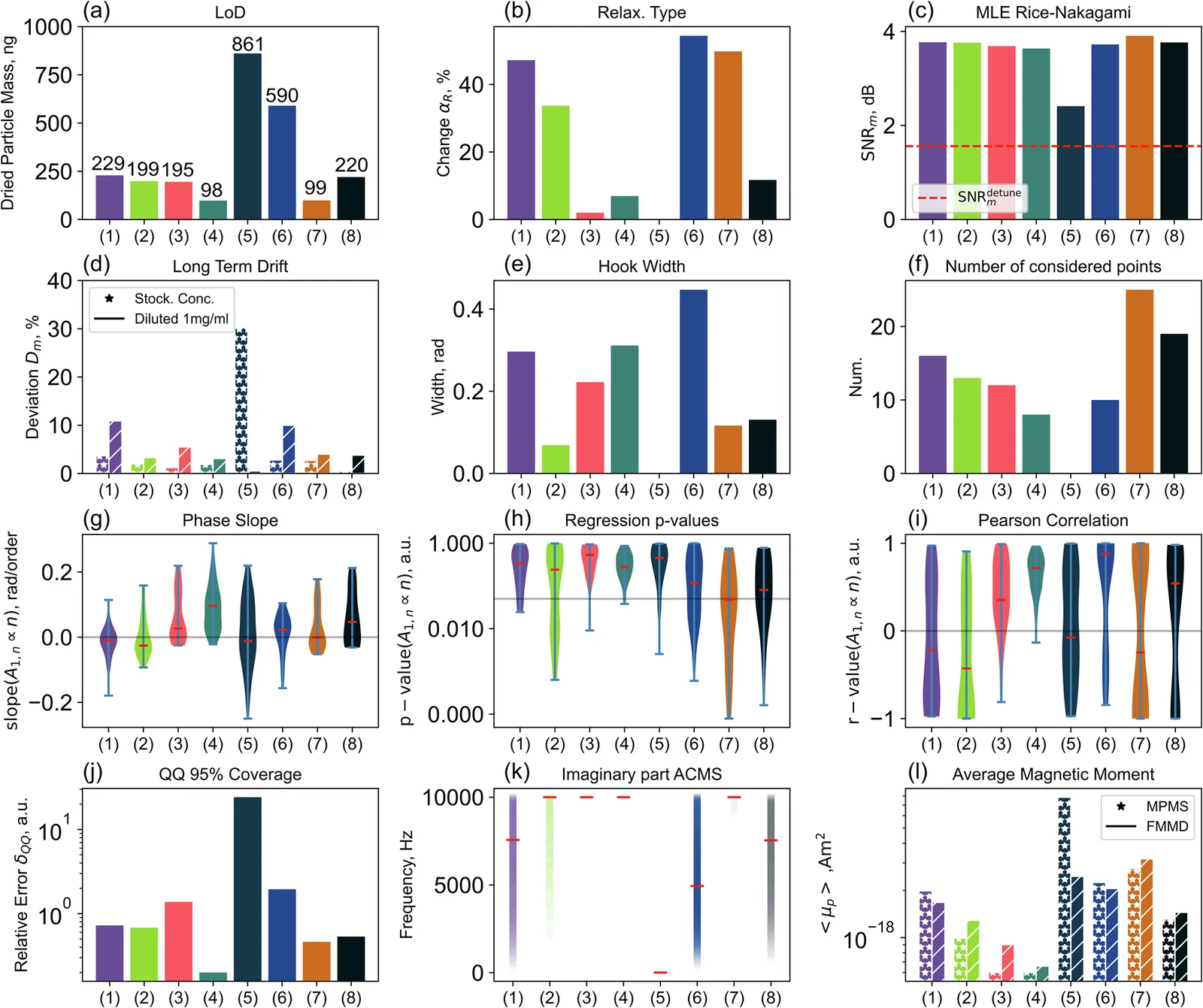
Measurement parameters of samples: (1) “ON 30 nm”, (2) “ON 25 nm”, (3) “ON 20 nm”, (4) “ON 15 nm”, (5) “ON 10 nm”, (6) “Co-Fe”, (7) “Syn-D 50 nm”, and (8) “Syn-D 70 nm”. **a** Limit of detection. **b** Relaxation type ${\alpha }_{{\rm{R}}}$. **c** Short-term SNR level determined using Rice-Nakagami distribution Eq. (16). **d** Long-Term Drift evaluated using Eq. (20). **e** The difference between the minimal and maximal argument of the ${A}_{1,2}$ response on the complex plane. **f** Number of points of LF-scan that have 20 dB SNR considered for reliable evaluation of linear phase dependency. **g** Distribution of slope of the phase vs. order of the harmonic obtained as a result of regression. **h** Distributions of *p*-values obtained as a result of regression. Gray line shows 5% confidence level. **i** Distribution of Pearson’s correlation coefficients obtained for LF-scans. **j** Evaluation of magnetic-moment distribution comparison. Denotes the relative error range that covers 95% of all distribution bins. **k** Imaginary part of ac-susceptometry normalized by the full amplitude of the response. Red stroke denotes the maximum of the imaginary part. **l** Average magnetic moments calculated in the FMMD-accessible range from the Magnetic Property Measurement System (MPMS) and FMMD techniques.

All MNP samples (cf. “Samples of magnetic nanoparticles”) were measured at logarithmically decreasing concentrations ranging from 1 mg/ml to 50 ng/ml with a dilution factor of 7.25 to identify the Limit of Detection (LoD). Dysprosium Oxide (III) was measured as a negative control sample. The concentration intercept point of crossing the noise floor and corresponding MNP mass were determined for all samples (Fig. 6a LoD). Samples “ON 15 nm” and “SynD-70” showed the highest detectability with 98 ng and 99 ng of dried particle mass of the detectable limit in 100 µL volume, respectively.

Signal quality and stability parameters were determined for all MNP types and a linear reference, Dysprosium Oxide (III). Short-term drift (${{\rm{SNR}}}_{m}$) is obtained using Eq. (16) for the measurement ${A}_{1,2}$ and is provided in the Fig. 6c for samples at stock concentrations. For comparison, the ${{\rm{SNR}}}_{m}$ value of a linear sample (Dysprosium Oxide) is ${{\rm{SNR}}}_{m}^{{{\rm{Dy}}}_{2}{{\rm{O}}}_{3}}=1.543\,{\rm{dB}}$, and the value for the detuned measurement at ${A}_{1,1.5}$: ${{\rm{SNR}}}_{m}^{{\rm{detune}}}=1.560\,{\rm{dB}}$. Long-term deviation (see Eq. (20)) on the level of a single measurement, ${D}_{m}$ was obtained for both stock concentrations and diluted samples (1 mg/mL) shown in Fig. 6d. For a linear sample, the long-term drift coefficient is ${D}_{m}^{{{\rm{Dy}}}_{2}{{\rm{O}}}_{3}}=49 \%$, due to signal amplitude comparable with noise floor level.

A regression was conducted to assess linearity between IMT’s phase ${\rm{arg}}({A}_{\cdot ,n})$ and its order n for all MNP samples at stock concentrations. The number of points considered for regression is given in Fig. 6f. For “ON 10 nm” linear regression could not be performed due to the low amplitude of the higher harmonics. Phase slope, regression *p*-value, and Pearson’s correlation are given in Fig. 6g, h, i, respectively.

It was observed that the linear dependence of the phases is stronger at the onset and end of the hook response. Sample “ON 25 nm” demonstrated the highest linearity metric Fig. 6i, defined in the “Evaluation of linearity of higher order terms” section, and almost no change in phase in LF-scan (${\rm{arg}}({A}_{\cdot ,n})\approx {\rm{const}}$) compared to other samples Fig. 6e.

### Varying viscosity and interrelation between FMMD and AC susceptometry

**Fig. 7:**
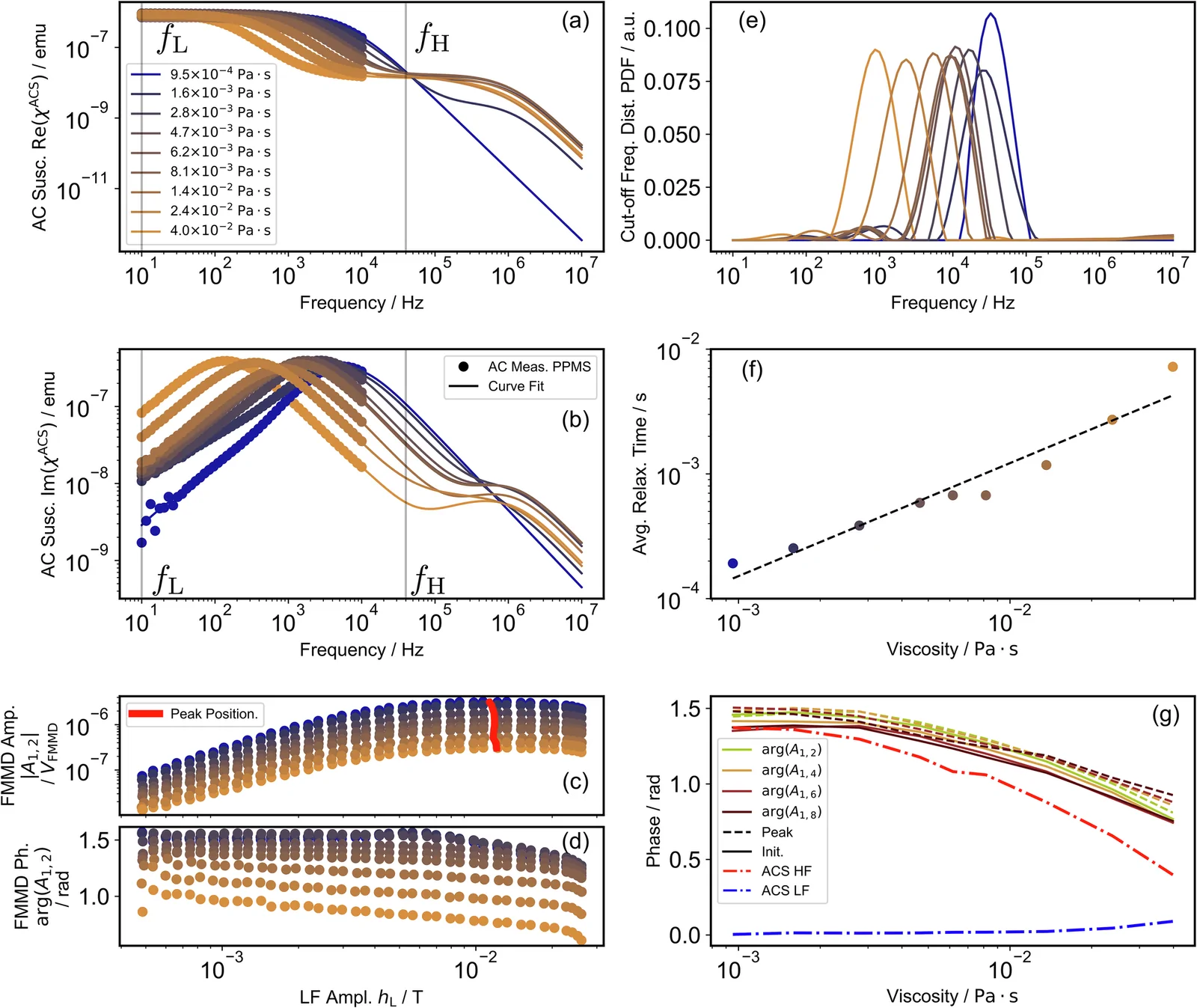
Relationship between AC susceptometry (ACS) and FMMD response for “Co-Fe” MNPs with dominating Brownian relaxation under gradual immobilization from increasing viscosities. Viscosity values are given in legend (**a**). Figures (**a**) and (**b**) show the real and imaginary components of AC susceptometry, respectively. Dots denote measured data, and the solid line denotes the model fit of the superposition of linear models with different relaxation times. Normalized distribution of the relaxation times is presented in (**e**), from which the average relaxation time for each viscosity case was obtained and plotted versus actual viscosity in (**f**). Plots (**c**) and (**d**) show the amplitude and phase of the FMMD signal, respectively, at the IMT ${A}_{{\mathrm{1,2}}}$. A thick red line shows the position of the maximum amplitude point (turnaround point of the hook) of the IMT ${A}_{{\mathrm{1,2}}}$. **g** Shows phases of FMMD IMTs and LF and HF phases derived from ACS. Solid lines denote asymptotic phases of the FMMD responses near small LF amplitudes. Dashed lines denote the phase of the turnaround point of the hook corresponding to the thick red line in (**c**). Phases for various IMTs are depicted simultaneously: (green) ${A}_{{\mathrm{1,2}}}$, (orange) ${A}_{{\mathrm{1,4}}},$ (light brown) ${A}_{{\mathrm{1,6}}}$, (dark brown) ${A}_{{\mathrm{1,8}}}$. Dash-dot lines denote ACS phases at HF (red) and LF (blue). The respective HF and LF frequencies are shown on ACS plots (**a**) and (**b**) with gray vertical lines. Due to the measurement device, the Physical Property Measurement System (PPMS), having a frequency range of 10 kHz, the HF phase was extrapolated from the fitted linear model.

By evaluating the responses of all MNP types in liquid and gypsum-fixed form, the relaxation degree parameter ${\alpha }_{{\rm{R}}}$ (Eq. (25)) was identified in Fig. 6b. The sample “Co-Fe” turned out to be the most sensitive to the changes in the Brownian relaxation time upon immobilization, with 54.4% variation. The least sensitive sample was “ON 20 nm”. All MNP sample types were measured with ACS, and the identified peak position (red line) in the imaginary part, shown with a gradient normalized to the amplitude of the ACS response, is provided in Fig. 6k. The sample “Co-Fe” showed the lowest Brownian relaxation time with a peak in the imaginary part at $4.9\,{\rm{kHz}}$. Figure 7 shows ACS results contrasted to FMMD ${h}_{L}$-scan measurements for the “Co-Fe” MNP system under gradual immobilization with increasing viscosities induced by increasing concentrations of glucose syrup in the carrier medium. Subfigures (a, b) depict the real and imaginary parts of the sample’s susceptometry ${\chi }^{{\rm{ACS}}}={\chi }^{{\prime} }+i\chi^{\prime \prime}$, and subfigures (c, d) depict FMMD’s amplitude and phase at ${A}_{1,2}$.

Despite increasing viscosities from water (${\eta }_{{\rm{wtr}}}=9.532\cdot {10}^{-4}\,{\rm{Pa}} \cdot {\rm{s}}$) to (${\eta }_{{\rm{syr}}}=39.8\cdot {10}^{-3}\,{\rm{Pa}} \cdot {\rm{s}}$; estimated for glucose syrup), the peak positions with respect to ${h}_{L}$-field amplitude remains almost unaltered in the range ${h}_{{\rm{peak}}}=11.7\pm 0.6\,{\rm{mT}}{\mu }_{0}^{-1}\,\left(\pm 3\sigma ,\,N=9\right)$, while the peak value dropped by approximately one order of magnitude from the pure water viscosity to the maximum syrup viscosity. The distribution of Brownian relaxation times ${\rho }_{\omega }\left({\omega }_{i}^{{{\rm{ACS}}}}\right)$ was obtained (cf. “Relaxation time distribution reconstruction from ACS”) in the form of normalized histograms given in Fig. 7e. Solid lines in Fig. 7a, b show the back-fitting of the model. From the inverse average of each histogram, the average Brownian relaxation time was calculated in dependence on the viscosity shown in Fig. 7f. Linear regression (dashed line in Fig. 7e) was conducted to verify the viscosity model.

The viscosity of the mixture ${\eta }_{{\rm{mix}}}\left(\alpha \right)$ was calculated as a geometric proportion between the viscosity of water and syrup ${\eta }_{{\rm{mix}}}\left(\alpha \right)={\eta }_{{\rm{syr}}}^{\alpha }{\eta }_{{\rm{wtr}}}^{1-\alpha }$ following the approach of Möddel^61^. The resulting relationship obtained using linear regression $\left\langle {\tau }_{B}\right\rangle \left({\eta }_{{\rm{mix}}}\right)={\eta }_{{\rm{mix}}}^{0.906}\cdot 79.4\cdot {10}^{-3}\,{\rm{s}}\left({\rm{p}}-{\rm{value}}1.8{\rm{e}}-5\right)$. Here $\left\langle {\tau }_{B}\right\rangle$ denotes the average value obtained using Eq. (24). For Brownian-relaxation-dominated particles, like “Co-Fe” here, direct proportionality between viscosity and (Brownian) relaxation time holds, $\tau \propto {\eta }_{{\rm{mix}}}$. Using the back-estimated viscosity of the syrup of ${\eta }_{{\rm{syr}}}^{{\rm{est}}}=28.0\cdot {10}^{-3}\,{\rm{Pa}}\cdot {\rm{s}}$, the resulting relationship between relaxation time and viscosity can thus be directly derived from Fig. 7g with $\left\langle {\tau }_{B}\right\rangle \left({\eta }_{{\rm{mix}}}\right)={\eta }_{{\rm{mix}}}\cdot 0.152\,{\rm{s}}$. The discrepancy between the viscosity estimates of syrup between the cases does not exceed 30%. The estimated hydrodynamic diameter equals ${d}_{{\rm{H}}}=\root 3 \of {2{\tau }_{B}{k}_{B}T/\pi {\eta }_{{\rm{mix}}}}\approx 73.5\,{\rm{nm}}$.

AC-susceptometry values at LF and HF (gray lines in Fig. 7a, b) were extracted, and their phases are provided as blue and red dash-dot lines in Fig. 7g together with phases of FMMD response for four IMTs. The solid line depicts the phase value at the peak value of the ${h}_{L}$-scan, and the dashed line corresponds to the asymptotic value of the phase for low-frequency amplitude values. The FMMD phases for IMTs, ${A}_{1,\cdot }$, change equally and simultaneously for all viscosity values in range $\left[0.742,\,1.505\right]\,{\rm{rad}}$, spanning a total of $0.763\,{\rm{rad}}$ with a standard deviation of $0.195\,{\rm{rad}}\,\left(3\sigma ,\,N=8\right)$ for the cases of largest deviations. In AC-susceptometry measurements, the low-frequency (LF) phase changes relatively modestly in the range of $85.3\cdot {10}^{-3}\,{\rm{rad}}$. In contrast, the high-frequency (HF) component exhibits significantly higher sensitivity, ranging from $0.294\,{\rm{rad}}$ to $1.495\,{\rm{rad}}$ corresponding to a total variation of $1.201\,{\rm{rad}}$.

**Fig. 8:**
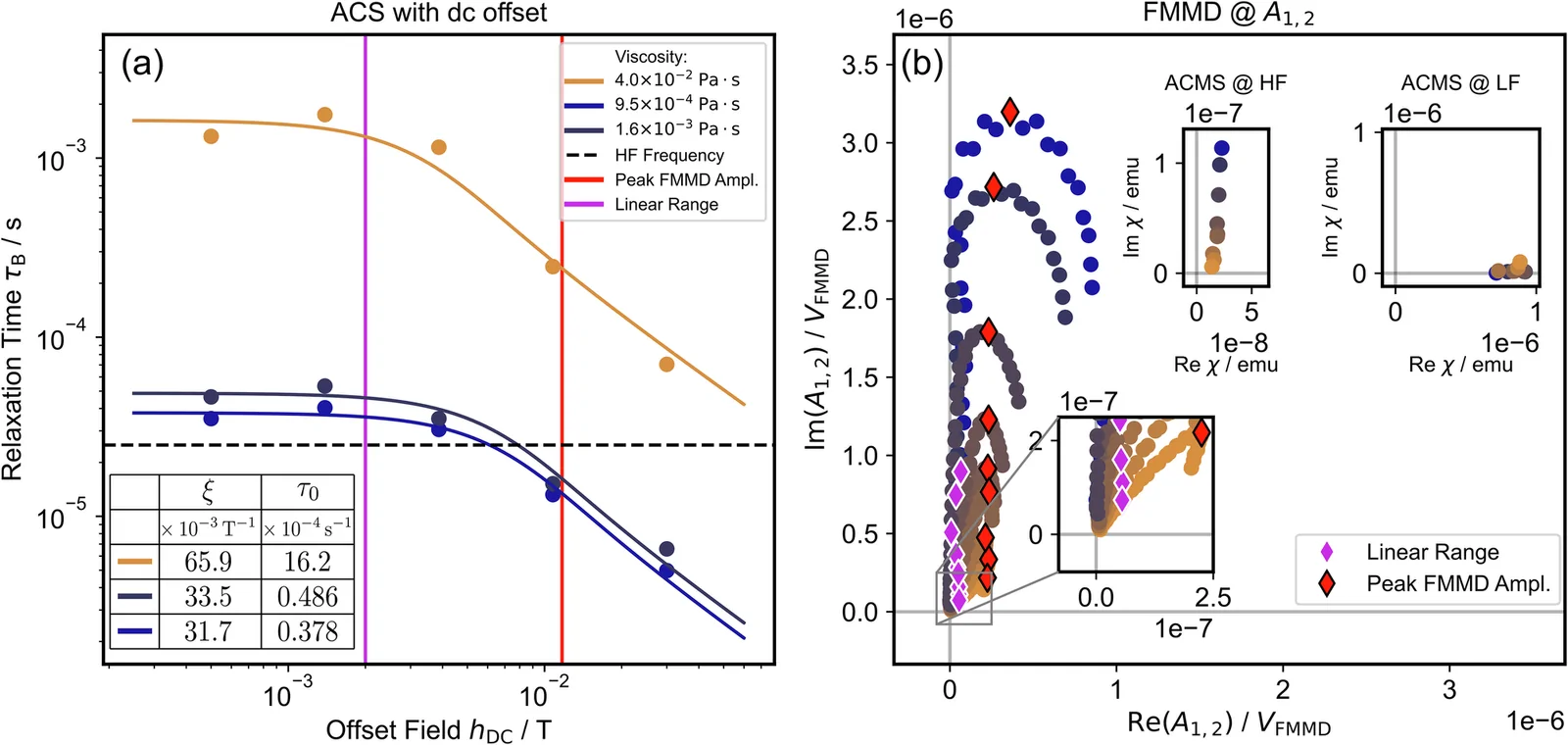
Comparing ACS and FMMD responses for sample “Co-Fe”: field dependency of Brownian relaxation time from ACS and formation of hook shape on FMMD response. **a** ACS-derived Brownian relaxation time as a function of offset field ${h}_{{\rm{DC}}}$ for three different viscosities between $(9.5\cdot {10}^{-4},\ldots ,4\cdot {10}^{-2})\,{\rm{Pa}}\cdot {\rm{s}}$. The red vertical line depicts the mean peak FMMD amplitude, $\left(11.7\pm 0.6\right)\,{\rm{mT}}/{\mu }_{0}$. The violet line marks the offset field below which the relaxation time remains approximately constant. **b** FMMD response shown on the complex plane for the same samples. Red diamonds mark the FMMD signal at peak amplitude, and violet diamonds mark the field to which the linear response regime holds up to which relaxation time remains constant. The two inserts (entitled “ACMS”) show the ACS response at HF (left) and LF (right), with the complex plane shown for comparison.

Figure 8 illustrates the relationship between frequency mixing magnetic detection (FMMD) with ${h}_{L}$-scan and AC susceptometry (ACS) with an offset component ${h}_{{\rm{DC}}}$ exemplified with the “Co-Fe” sample. The figure highlights the comparability in measuring the effects of amplitude-driven phase modulation and its spectral consequences on both dynamic magnetometry techniques. In Fig. 8a, the average Brownian relaxation times (obtained as described above) are plotted as a function of the offset field ${h}_{{\rm{DC}}}$ for three different viscosities. The solid lines represent fits to Eq. (26) with fitting parameters ξ and ${\tau }_{0}$ given in the Table inside Fig. 8a. A vertical violet line marks the region up to which the linear response regime is assumed to hold, and up to which the relaxation times remain constant. Beyond that limit, the field dependence of relaxation time must be considered (as discussed in the “Introduction”, Eq. (15)). A vertical red line corresponds to the field amplitude at which the FMMD response peaks are obtained from Fig. 7c.

In Fig. 8b, the first IMT ${A}_{1,2}$ derived from FMMD measurements of “Co-Fe” at various viscosities are plotted in the complex plane. Two points of interest are marked: The red diamond denotes the peak amplitude of the FMMD response, corresponding to the red line in Fig. 8a. The violet diamond identifies the field range up to which the linear regime holds (<2 mT) beyond which relaxation time becomes field dependent. Within the linear regime, the FMMD response follows a straight trajectory consistent with the Wiener model behavior, as depicted in row 4 of Fig. 1 and in Fig. 2c.

However, beyond the violet diamond, the increasing field amplitude begins to modulate the relaxation time, resulting in the phase shift already predicted in Fig. 2c in our proposed model. This phase shift leads to a clockwise bend, or “hook,” in the complex trajectory of the FMMD signal response. The inserts in Fig. 8b further contextualize this behavior in the AC-susceptometry response at HF (left) and LF (right) with the following observations:

- The top-left inset shows the ACS response at high frequency (HF), which closely resembles the peak positions of the FMMD response (red diamonds).
- The top-right inset shows the ACS response at low frequency (LF), where the ACS response lies on the real axis and remains independent for various viscosities.
- The bottom-left inset provides a magnified view of the FMMD hook region, demonstrating the “hook” shape also for the smallest FMMD signals at the highest viscosities.

### Evaluation of magnetic moment resolution

**Fig. 9:**
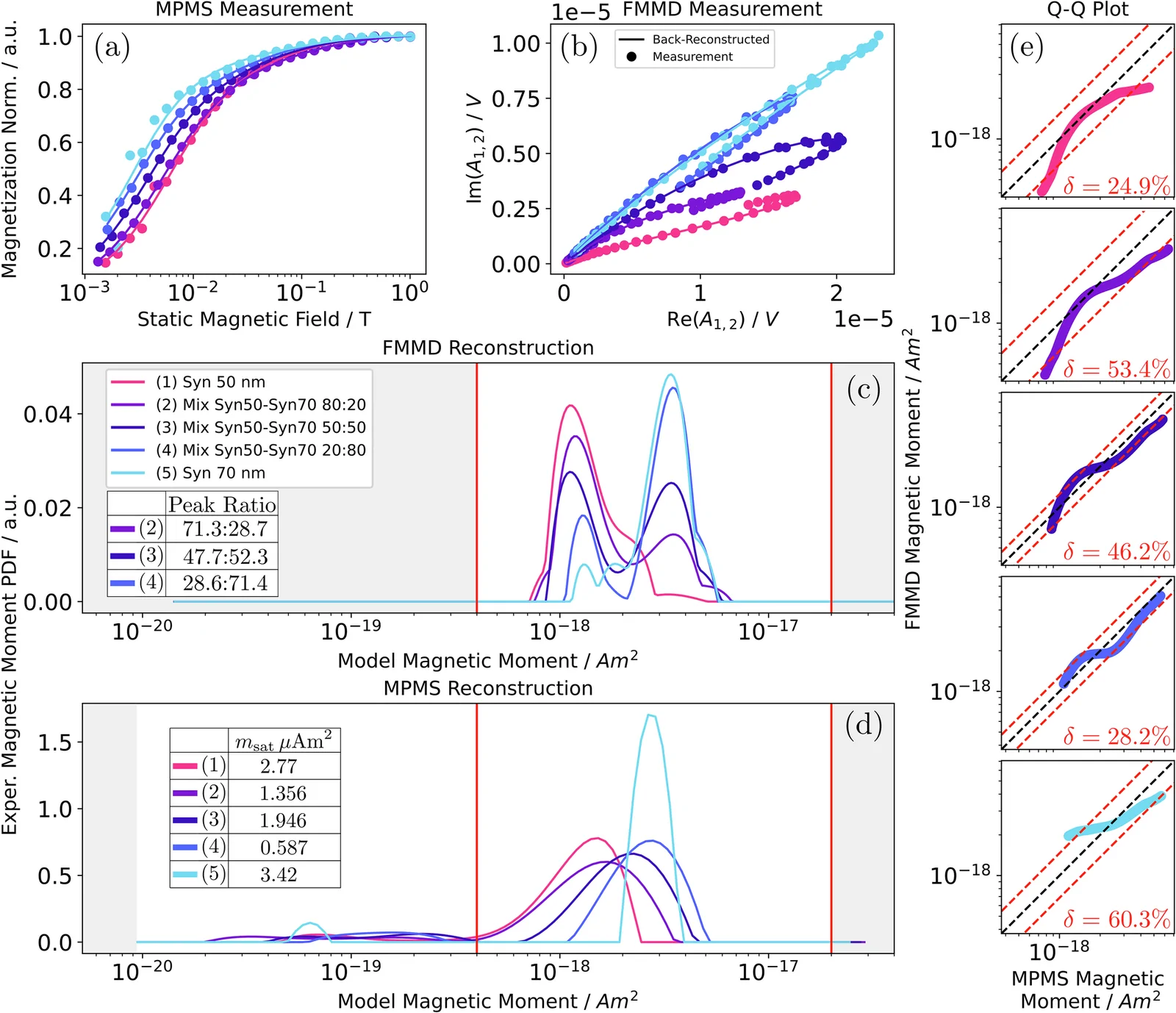
Evaluation of magnetic moment resolution of FMMD. **c** Distribution of magnetic moments obtained from samples of Synomag-D 50 nm (#1) and Synomag-D 70 nm (#5) and their mixtures (#2,3,4), reconstructed from FMMD response ${A}_{{\mathrm{1,2}}}$, presented in (**b**). Dots: raw measurement. Solid lines: back-reconstructed signal. **d** The distribution of magnetic moments is reconstructed from static magnetization curves for the same samples. **a** contains corresponding static magnetization curves. Table (**d**) shows the saturation magnetization moments of the samples. Red lines in (**c**, **d**) denote the trustworthy reconstruction region of FMMD. Column (**e**) shows a quantile-quantile comparison between distributions. Red dashed lines denote the relative error that includes 95% of all distribution points.

Figure 9 presents a comparative evaluation of the ability to reconstruct the magnetic moment distribution from dynamic dual-frequency excitation (via FMMD) vs. static magnetometry (via MPMS), as exemplified for samples with varying core diameters: “Syn-D 50 nm”, “Syn-D 70 nm”, and their mixtures. The numbers in their titles denote their hydrodynamic sizes provided by the manufacturer and do not reflect their magnetic core sizes, which determine their magnetic properties. The batches of these MNP samples were selected, as they demonstrated the narrowest non-overlapping magnetic moment distributions reconstructed from both FMMD and static magnetization (MPMS) measurements (see below) among the MNP types presented in Supplementary Fig. 1. Thus, these two types were suitable for experimental evaluation of resolution in magnetic moment domain. Their mixtures were prepared as described in the “Methods” section, and their magnetic moment distributions were reconstructed using MPMS and FMMD.

Figure 9a shows the static magnetization curves obtained for the samples via MPMS (cf. “Measurement of the static magnetization curve”), plotted as sample magnetization normalized to the maximum value versus the applied static DC magnetic field. The model back-reconstructed response (solid) overlaps with the experimental data (dots). The inset in Fig. 9d contains the estimated values of the saturated magnetic moment. The static curves of the original constituents, “Syn-D 70 nm” (#5) and “Syn-D 50 nm” (#1), appear distinct: the onset for “Syn-D 70 nm” (#5) takes place at smaller field values compared to “Syn-D 50 nm” (#1), due to a larger average magnetic moment. All their mixtures lie between the original curves, shifting proportionally to the volumetric ratio of the constituents.

Figure 9b demonstrates the corresponding FMMD measurements in the complex plane (see “Measurement of intermodulation terms”). The initial constituents (#1) and (#5) have narrow “hook” widths following the Wiener model, whereas their 50:50 mixture (#3) demonstrates increased width. Such behavior corresponds to the case discussed in Fig. 2c, where a mixture of two samples that follow a simpler Wiener model yields a more complex amplitude-driven phase modulation.

In Fig. 9c, the FMMD-based magnetic moment reconstructions are shown (cf. “Reconstruction of magnetic moment distribution from FMMD”). The red vertical lines denote the trustworthy resolution range of the FMMD method (cf. “Regularized reconstruction”) beyond which peaks cannot be reliably localized and must be considered as inaccessible background. The mixture samples (#2, #3, #4) are reconstructed as bimodally distributed without any prior knowledge of the original constituents, meaning that the FMMD measurements for #1 and #5 were not used in the reconstruction at all. The heights of the peaks in bimodal distributions (#2, #3, and #4) scale proportionally to the volume proportion of the corresponding constituent. The inset table in Fig. 9c shows the amplitude ratios of the reconstructed bimodal distributions, which can be directly compared with the volume ratios given in the legend. The position of the peaks of bimodally distributed samples (#2, #3, and #4) lies in the vicinity of the peaks of original constituents (#1 and #5). Constituent (#1) has a peak at $1.113\,{\rm{aA}}{{\rm{m}}}^{2}$, and the peaks of bimodal distributions lie in the range $1.187\pm 0.199\,{\rm{aA}}{{\rm{m}}}^{2}\,(\pm 3\sigma ,N=3)$, whereas the constituent (#5) has peak at $3.411\,{\rm{aA}}{{\rm{m}}}^{2}$ and the corresponding peaks of bimodal distributions lie in the range $3.455\pm 0.0903\,{\rm{aA}}{{\rm{m}}}^{2}\,(\pm 3\sigma ,N=3)$. Thus, relative error in peak determination equals 6.64% and 1.28% for constituents (#1) and (#5), respectively. The critical regularization parameter beyond which the mixture is considered as unresolvable was determined using the procedure “Evaluation of the system’s resolution for mixed particle systems.” and equals ${\lambda }_{{\rm{crit}}}=0.3$.

The corresponding magnetic moment distribution reconstructions for MPMS-based measurements are shown in Fig. 9d. The initial constituents (#1 and #5) are resolved as distinctive peaks, with their positions overlapping those of the corresponding peaks in the FMMD-reconstructed distributions (Fig. 9c). The comparison between two types of reconstruction is conducted using per-quantile comparison (Q-Q plots) shown in Fig. 9e, obtained as described in “Distribution comparison”. The figure sequence corresponds to the order of the samples in the legend. The relative error δ that accommodates 95% of all distribution bins serves as a cumulative figure of merit of a coincidence between distributions, and is depicted with red dashed lines. Note that the relative error is used because of the logarithmic scaling of the magnetic moment distribution. For initial constituents “Syn-D 50 nm” and “Syn-D 70 nm”, these errors equal 53.4% and 46.2%, respectively. Additional cross-sample comparisons throughout each reconstruction method is provided in the supplementary materials in Supplementary Fig. 6 and Supplementary Fig. 7. From these comparisons, one can see that two distributions can be considered relatively alike if the limit relative error does not exceed $\delta \le 100 \%$ and the curve crosses the diagonal line. Note that all Q-Q plots have a crossing with a diagonal, indicating a cogent degree of agreement in FMMD- and MPMS-derived magnetic moments. Furthermore, taking into account the comparison in Fig. 6l, we conclude that FMMD captures the correct order of average magnitude of magnetic moment evaluated in the FMMD-accessible region for all particle types used in this work except “ON 10 nm” which appears to have a small average magnetic moment lying outside of the FMMD-accessible range. The mixtures were reconstructed as unimodal distributions, unlike FMMD-based reconstruction, with the peak positioned between the initial peaks and shifting proportionally to the relative volumetric ratios of the constituents, consistent with the observation from the static magnetization curves.

These magnetic moment distributions should not be interpreted literally in all cases. From a statistical physics perspective, they represent the partition function $Z\left(H,\varphi \right)$, where each magnetic-moment bin ${\mu }_{i}$ contributes to the corresponding energy level ${\mu }_{0}{\mu }_{i}H$ with weight ${C}_{i}$,

29 $$Z\left(H,\varphi \right)=\mathop{\sum }\limits_{i}{C}_{i}{e}^{\frac{{\mu }_{i}{\mu }_{0}H}{{k}_{{\rm{B}}}T}\cos \varphi }$$

For a magnetic colloid composed of several non-interacting particle types, each individually obeying the Langevin magnetization model, the magnetic-moment distribution interpretation naturally arises from the corresponding partition-function formulation. When additional energy levels appear, i.e., as a result of particle interactions or internal magnetic anisotropy, the validity of this interpretation deteriorates.

The resolution analysis, conducted using the Backus-Gilbert method^62^ (see “Resolution analysis” in supplementary materials), for both FMMD and MPMS reconstructions with the used regularization parameters, yielded resolution factors of 2.68 (Supplementary Figs. 5) and 3.65 (Supplementary Fig. 4), respectively. These factors denote full-width-at-half-maximum (FWHM) in logarithmically scaled magnetic moment space: two monodisperse constituents differing in their magnetic moments by this factor will be resolved. Recall that the regularization parameter is fixed for both MPMS and FMMD reconstructions to λ=0.1, where it was chosen such that the factual ${{\rm{FWHM}}}_{{\rm{Syn}}70{\rm{nm}}}\approx 1.9$ of the narrowest constituent (“Syn-D 70 nm”, see Fig. 9c, d) is approximately the same. Simultaneously, this regularization level safely exceeds by an order of magnitude the noise-determined lower boundaries determined in Backus-Gilbert analysis (Supplementary Fig. 3c), which equal $\sim {10}^{-3}$ and $\sim {10}^{-4}$ for MPMS and FMMD, respectively, when 1% relative error noise level is targeted. Thus, the obtained results can be characterized as being rather overregularized than overfitted. The FMMD-based MMD reconstruction has a narrower trustworthy resolution range (see the resolution matrix in Supplementary Fig. 3a) but appears more stable to noise in the data than the MPMS-based one. The latter was obtained by analyzing the diagonality metric of the resolution matrix (Supplementary Fig. 3b) for both measurement methods under various additive noise conditions (Supplementary Fig. 3c). Stability is quantified as the lower bound on the regularization parameter, above which the resolution matrix’s diagonal elements smoothly depend on it.

For comparability with other works^17,18,63–66^, a non-negative least-squares-based linear decomposition of the mixture signals over the initial constituents was performed for both MPMS and FMMD measurements, with results summarized in Supp. Tab. 1 in supplementary materials. The relative deviation between the expected volumetric fraction and the obtained ratio did not exceed 10% for both methods and appeared approximately identical for both methods.

Finally, the samples (#1) and (#5) were used for calibration of coupling coefficients as described in “Calib. of MMD”. MPMS coupling coefficients obtained for (#1) and (#5) are equal to ${c}_{{\rm{m}}}^{{\rm{MPMS}}}=1.334\,{\rm{A}}{{\rm{m}}}^{2}$ and ${c}_{{\rm{m}}}^{{\rm{MPMS}}}=1.321\,{\rm{A}}{{\rm{m}}}^{2}$, respectively, demonstrating $\sim 1 \%$ difference. Estimates of lower boundaries of FMMD coupling coefficients showed a higher deviation of $\sim 29 \%$ with values ${c}_{{\rm{m}}}^{{\rm{FMMD}}}=1.269\,{\rm{A}}{{\rm{m}}}^{2}$ and ${c}_{{\rm{m}}}^{{\rm{FMMD}}}=1.647\,{\rm{A}}{{\rm{m}}}^{2}$ for (#1) and (#5), correspondingly.

## Discussion

In this study, we proposed a method for enhancing the resolution of MNP magnetic moment estimation by directly assessing higher-order derivatives of the magnetization curve. The technique relies on the synchronous measurement of intermodulation spectral terms (IMTs) that emerge in the magnetization response under dual-frequency excitation. Although the direct assessment of higher-order derivatives through synchronous IMT measurement is well known in other fields^67^, to the best of our knowledge, this is the first time it has been applied to the non-destructive reconstruction of the magnetic moment of MNPs with enhanced resolution.

Developed within a modulation theory framework using fundamental geometric assumptions, the polynomial equilibrium model introduced in this work provides an interpretational basis, allowing direct assessment of the magnetic moment of MNP from the raw FMMD signal. In this regard, our work extends the previous work of Ilg^68^ and Sun^69^, who also used polynomial models to explicitly interpret higher-order spectral terms. The former work remarks that the phase of MPS response is independent of excitation field amplitude, upon which our work adds interpretational range by addressing the spectral phase symmetries and their application to interpreting the FMMD signal. The latter work proposed linearly spaced amplitude scans for MPS to reconstruct the magnetic moment distribution, an approach we further developed here and provide supporting evidence that logarithmic field-amplitude sampling maps the FMMD raw signal to magnetic moment space, allowing its accurate reconstruction with increased resolution. Explicitly, we exploit the spectral symmetries for amplitude and phase modulation that arise from the interplay of finite magnetizability and relaxation processes and appear as corresponding components in the complex envelope of the measured FMMD signal. We exploit these symmetries experimentally and demonstrate that the shape of the magnetic response of MNP during FMMD, when plotted in the complex plane (coined “hook”-plot), contains directly interpretable input on whether the MNP sample is governed by an equilibrium behavior or else behaves like a more complex system governed by amplitude-driven phase modulations (cf. Figure 2c). In fact, the latter appears as curvature points in the complex plane (and simultaneously in the spectral domain), forming an asymmetry around the carrier frequency directly originating from the dual-frequency excitation field (as shown in Fig. 9b and Supplementary Fig. 1b).

To capture the aforementioned signals, we introduced an FPGA-based measurement setup that enables reliable synchronous amplitude and phase demodulation of dual-frequency-induced intermodulation terms (IMT). To allow IMT measurement in a narrow band around HF, an interference-compensation technique was proposed in “Active cancellation of interference,” achieving a 79× reduction in interference amplitude. The necessary amplitude requirement for synchronous measurements is exceedingly fulfilled by at least three orders of magnitude over the noise floor (${\rm{RMS value}}:\, \sim {10}^{-9}\,{{\rm{V}}}_{{\rm{FMMD}}}$) for all samples, except “ON 10 nm”. This sample’s deviation toward a more substantial influence due to noise is attributed to its experimentally measured small magnetic moment in Supplementary Fig. 1d. This translates directly to the sample having the smallest mean particle size, which is also confirmed by simulations to diminish the overall FMMD signal generation^20^, thereby increasing susceptibility to noise. The fulfillment of the sufficient phase noise requirement is reflected by the maximum-likelihood-estimated Rice-Nakagami average Signal-to-Noise Ratio (SNR) parameter of all samples fulfilling the necessary amplitude requirement (s. Figure 6c), yielding at least 2 dB gain compared to the noise floor (${{\rm{SNR}}}_{m}^{{\rm{detune}}} \sim 1.5\,{{\rm{dB}}}$). Furthermore, the relative deviation in signal stability during long-term measurements for each individual MNP sample, cf. Figure 6d, does not exceed 10% for stock or even 3% for the normalized concentration of each sample. These levels confirm an acceptable degree of long-term stability for the measurement technique and for each commercial sample used. The percentage of relative change also falls within the range of deviation between measurements on identical MNP via magnetic particle spectroscopy (MPS) and imaging (MPI), reported by Paysen et al.^70^ to be 5% or lower, suggesting that our proposed method is comparable to these established techniques.

Based on the phase symmetries of the polynomial equilibrium model described by Eq. (8), a method is proposed for identifying the physically relevant directions of real and imaginary axes on the complex plane of the FMMD response matching with ACS measurements, exemplified for sample “Co-Fe” (cf. Figure 7). The mutual correspondence between both techniques after phase correction is shown in Fig. 8b, where both the FMMD response and the ACS are probed at HF and LF, are plotted on the complex plane. Across samples with varying viscosity, the geometry of FMMD responses replicates the distribution of ACS responses at HF. The differences between the phases of higher-order IMTs in Fig. 7g appear insignificant, remaining generally congruent, consistent with the theoretical model Eq. (26). Reduction of Brownian relaxation time by more than one order of magnitude caused by viscosity alteration (cf. Figure 7f) is in accordance with theoretical and experimental predictions for single-frequency excitation^71^. Applied here to a dual-frequency excitation, the FMMD response scales and rotates without affecting the field dependence of the phase modulation, exactly as the latter part of Eq. (26) suggests. As a result, the field value corresponding to peak position (Fig. 7c) remains almost invariant with viscosity variations. Thus, the relative changes in FMMD-response’s phase can be attributed to the field dependence of relaxation time, while the average direction of the response on the complex plane corresponds to ACS HF susceptometry. The former is in accordance with single-frequency excitation, as shown by Yoshida and Enpuku^71^, while the latter allows for an estimation of the hydrodynamic size of the probed particles^72^. Indeed, by modeling the influence of varying viscosity, we have shown that the change in the relaxation behavior due to gradual immobilization of MNP shifts the peak position of the highest signal on the complex plane, but it does not change the nonlinear properties of the static magnetization curve. Thus, the EF amplitude corresponding to the peak remains unaffected, as shown by the red line in Fig. 7c. A similar physical model was used in the work of Dieckhoff^73^, who also analyzed AC susceptibilities under various Néel and Brownian relaxation times. These results coincide with those of Model^61^ in terms of signal loss and with those of Sun in terms of the Brownian relaxation time^16^. The latter work also shows that the reconstructed magnetic moment remains independent, whereas the reconstructed Brownian relaxation time varies. Finally, the work provided a comparison between the static magnetization curve obtained from MPMS and the dynamic magnetization behavior assessed via FMMD. Applying a distribution-weighted Langevin curve equilibrium model that combines static and dynamic magnetization curves enables comparison of the reconstruction performance of magnetic moment distributions from both measurements. Acknowledging the accessible range within which FMMD is able to trustworthily reconstruct the magnetic moment distribution, our results suggest that FMMD yields a magnetic moment distribution with improved resolution, as compared to MPMS (see Fig. 9c, d). This observation is further confirmed when comparing the magnetic moment reconstructions for samples of predetermined mixing ratios of known samples (Fig. 9). Here, FMMD demonstrates improved resolvability by allowing bimodal sample identification (which static MPMS fails to resolve). Furthermore, FMMD is able to quantify the mixing ratio: In fact, the 50:50 mixture was experimentally reconstructed as a mixture with ratio 52.3:47.7, the 80:20 mixture was reconstructed with 71.4:28.6 ratio and, finally, the 20:80 mixture was resolved with 28.7:71.3 ratio of peaks, yielding a maximum deviation of 8.7% from the expected mixing ratios.

Currently, as reported in the literature, mixture analysis is conducted by decomposing the measured response into a linear combination of the responses of the initial constituents, as in the works of Rauwerdink^17,18^, Achtsnicht^54^, Pourshahidi^74^, and Viereck^65^, which require a priori assumptions. The relative concentration estimation errors reported range from 2.3% by Tu et al.^18^ to 10% by Viereck et al.^65^. For compatibility, we conducted a similar straightforward linear decomposition for both MPMS- and FMMD-measured mixtures. The worst-case error does not exceed 11% for the FMMD case and 10% for the MPMS case. We consider a priori widely accepted MPMS measurements to be more reliable and attribute the observed comparable method-wise errors to the possibility that some particles interacted, leading to a deviation from a pure linear combination. In the supplementary materials, we provide additional mixing experiments in which we mixed Ocean Nanotech 30 nm and 20 nm samples. These samples appear stable when kept separate, but completely sediment in ~1 h after mixing. On the reconstructed distributions of these samples, one can clearly observe the appearance of magnetic signals that would stem from new fictional particles with smaller magnetic moments than those appearing in the individual, non-mixed MNP samples. This effect can be attributed to the particles’ dipole-dipole interactions in clusters that create additional energy barriers, requiring a higher field amplitude for remagnetization and hence appearing as smaller particles in our measurements. This experiment demonstrates that linear decomposition cannot identify such a change in the mixture.

In contrast to the conventional linear decomposition approach, our method can differentiate bimodal sample distributions without a priori assumptions or additional information about the constituent components of the sample mixture. In the FMMD-accessible range 0.311–8.09 aAm^2^, three-fold separated constituents potentially can accommodate three contrast lines. This approach can therefore facilitate the accurate characterization of heterogeneity in particle size, composition, and magnetic anisotropy, providing valuable information for synthesis and quality control, which is essential for applications in biosensing, magnetic hyperthermia, and magnetothermal stimulation. Furthermore, the method may allow for assessing well-characterized MNP systems for the onset of particle-particle interaction effects (as discussed and shown for the combination of 20 and 30 nm ON particles above) thus serving as a convenient means for rapid, non-destructive quality control and assurance of large(r) scale MNP production or individual characterization for research application as well as scale-up synthesis for commercialization processes.

Further work should include the interpretation of the multimodal peaks appearing in the magnetic moment distributions of physically monodisperse samples. We assume that these peaks can be attributed to anisotropy barriers in the particles, depending on material, size, and shape^50^, potentially allowing us to estimate the effective anisotropy from FMMD measurements as well. Samples that act as described by the Wiener model, i.e., without amplitude-driven phase modulation, enable multicontrast dynamic information extraction, such as simultaneous measurement of viscosity and temperature, as already demonstrated by Draack et al. individually for viscosity^75^ and temperature^76^ in MPS. Additionally, assuming spherical particles, the magnetic moment can be easily converted to a mean particle core size, allowing magneto-physical particle analysis.

In this way, the framework proposed in this work holds great promise for FMMD-based material characterization of MNP samples and meaningful advances for biomedical applications. We note, however, that exact requirements on particle systems are needed for such multi-dimensional analysis, whose concrete formulation remains to be carefully determined.

## Supplementary information

Transparent Peer Review file

Supplementary materials PDF

## Data Availability

The measurements of the magnetization curves, ac-susceptometry measurements, and dual-frequency-nonlinearity probing measurement data including the post-processing scripts that support the findings of this study are available in Jülich DATA repository with the identifier 10.26165/JUELICH-DATA/93REE6^77^.

## Code availability

The increase in resolution in magnetic moment space, central to this work, is demonstrated using a proposed algorithm for solving the inverse problem with zero-order regularization. The code implementing corresponding mathematical procedures described in the Methods sections “Regularized reconstruction”, “Reconstruction of magnetic moment distribution from FMMD”, and “Reconstruction of magnetic moment distribution from MPMS” is available in Jülich DATA repository with the identifier 10.26165/JUELICH-DATA/93REE6^77–82^.
