# Supplementary material for: Enhanced magnetic moment discrimination for multiplex nanoparticle quantification via dual-frequency nonlinearity probing
Source: Commun Eng. 2026 Jun 30;5:117. doi: 10.1038/s44172-026-00713-5 (PMC13319186; doi:10.1038/s44172-026-00713-5)
Supplement: Supplementary file 2 — Supplementary materials PDF [file 44172_2026_713_MOESM2_ESM.pdf]

communications engineering

Article

A Nature Portfolio journal

Article

https://doi.org/XX.XXXX/XXXXXX-XXX-XXXXX-X

Supplementary Materials: Enhanced magnetic moment discrimination for multiplex nanoparticle quantification via dual-frequency nonlinearity probing

Received: xxxx

Accepted: xxxx

Published online: xxxx

Check for updates

Timur I. Bikulov [1,2], Ulrich M. Engelmann[3], Andreas Offenhäusser [1,2] & Hans-Joachim Krause [1,3]

1 Institute of Biological Information Processing, Forschungszentrum Jülich, Jülich, Germany
2 Faculty of Mathematics, Computer Sci. and Natural Sci., RWTH Aachen University, Aachen, Germany
3 Department of Medical Engineering and Applied Mathematics, FH Aachen University of Applied Sciences, Jülich, Germany
Corresponding author: h.-j.krause@fz-juelich.de; timur.bikulov@rwth-aachen.de

Advanced applications of Magnetic nanoparticles (MNPs) in biomedicine based on multiplex MNP distinction require accurate, model-agnostic characterization of their magnetic moment distributions (MMDs). However, the resolving power of conventional MMD reconstruction from a static magnetization curve remains literarily underexplored. Moreover, due to particle-particle interactions, the response of the particle mixture might differ from the linear combination of the original constituents. We explore resolution enhancement in magnetic-moment space by directly probing higher-order magnetization derivatives, benefiting from their ever-increasing field-domain localization. Nonetheless, the direct derivative probing, as it is inevitably conducted dynamically, poses an interpretive problem for the origin of the nonlinearities. Spectral symmetries arising solely under dual-frequency excitation reflect the corresponding origins of amplitude- and rate-related nonlinearities. Using a dedicated experimental setup capable of synchronous demodulation of intermodulation terms, the method is tested on commercial MNP samples and benchmarked with conventional AC-susceptometry and static magnetization data. The binary mixture ratio was quantified with 8.9% deviation, without any prior information about the initial constituents differing by a factor of 3 in their average magnetic moments, potentially allowing the accommodation of three independent contrast channels for multiplex MNP applications as well as qualitatively probing magnetic interaction effects.

Supplementary Note 1 : Magnetic Moment Evaluation of Standard Particle Samples

A comparative analysis of magnetic moment reconstructions similar to that presented in Fig. 9 was conducted for samples with various core diameters: “ON 10-30 nm” and “Co-Fe”. The results are presented in Supp. Fig. 1. The normalized static magnetization curves (Supp. Fig. 1, a) were obtained using MPMS as described in ”Meas. of Stat. Mag. Curve”. The estimated saturation magnetic moments are given in the inset of Supp. Fig. 1 (d). The magnetization curves have distinctive shapes, among which the “ON 10 nm” shows its onset, defined as the point of maximum slope, at the highest fields, whereas the “Co-Fe” sample shows its onset at lower fields. The position of onset indicates that “ON 10 nm” and “Co-Fe” samples have the lowest and the highest magnetic moments among the samples examined, respectively. Other samples have their curves concentrated around the same point. Notably, the samples “ON 15 nm” and “ON 20 nm” overlap in their curves at low fields.

In Supp. Fig. 1 (d), the MPMS-based reconstructions are presented (cf. “Reconstruction of magnetic moment distribution from MPMS”). All samples except “ON 10nm” appear as bimodally-distributed with primary peaks in the FMMD-visible range (marked with red vertical lines) and secondary peaks situated in the range $10^{-20} \ldots 10^{-19}\ \mathrm{Am}^2$. In the FMMD-visible range, the peaks appear without bifurcation. The mutual position and order of the obtained reconstructed primary peaks conform with the shapes of the magnetization curves, confirming that the “Co-Fe” sample has the largest magnetic moment among all samples.

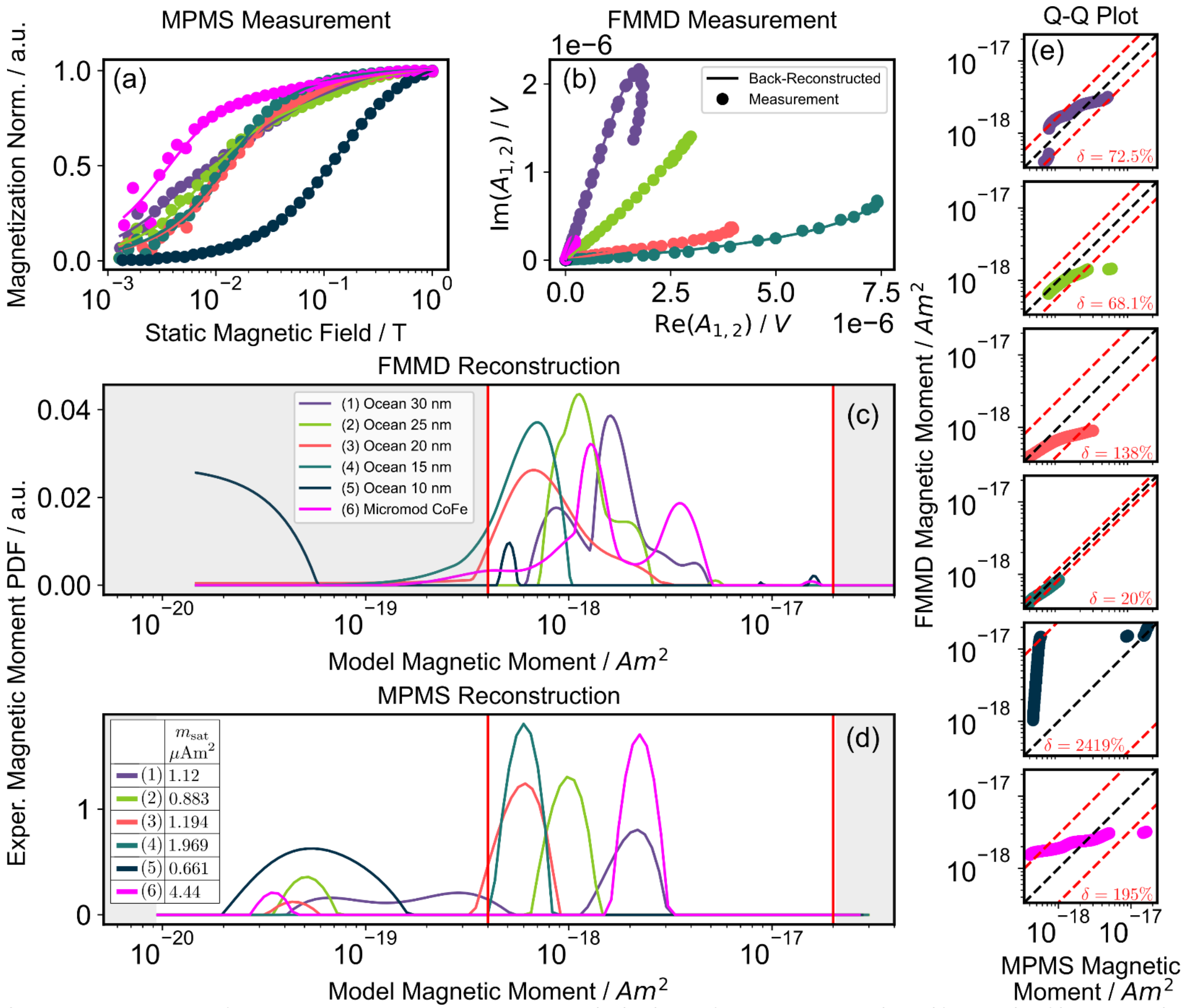

Supp. Fig. 1 | Reconstruction of MMD: FMMD versus static magnetization curve. (c) The distribution of magnetic moments is obtained from samples of Ocean Nanotech MNPs (#1-#5) and Synomag “Co-Fe” MNPs (#6), reconstructed from FMMD response $A_{1,2}$, and presented in (b). Dots: raw measurement. Solid lines: back-reconstructed signal. (d) The distribution of magnetic moments is reconstructed from static magnetization curves for the same samples. (a) contains corresponding static magnetization curves. Table (d) shows the saturation magnetization moments of the samples. Red lines in (c,d) denote the trustworthy reconstruction region of FMMD. Column (e) shows a quantile-quantile comparison between distributions. Red dashed lines denote the relative error that includes 95% of all distribution points.

Supp. Fig. 1 (b) demonstrates the corresponding FMMD measurements, where the dots denote the measured signal and the solid line is the back-reconstruction from the model fit. All samples except “ON 10 nm” produce strong nonlinear signals $+20..+40$ dB larger than the background signal. The samples demonstrate heterogeneous behaviour of the phase modulation character: Samples “ON 30 nm” and “Co-Fe” demonstrate a pronounced clockwise bending of the hook, whereas samples “ON 15 nm” and “ON 20 nm” demonstrate counterclockwise bending with a lower magnitude, and the “ON 25 nm” appears almost straight, following the Wiener model. In Supp. Fig. 1 (c), the FMMD-based MMD reconstructions are plotted. Similar to MPMS, the FMMD-based

results also show non-monodisperse distributions, but with bifurcated peaks (for samples “Co-Fe” and “ON 30 nm”) inside the FMMD-accessible range marked with red lines. Other peaks appear broader than in the MPMS-based reconstruction. The comparison between FMMD- and MPMS-based reconstructed distributions is conducted using Q-Q plots in Supp. Fig. 1 (e). Here, the relative errors, δ, vary significantly across samples, ranging from 20% (for “ON 15 nm” as the best match) to over 2400% (for “ON 10 nm”, which lies outside the FMMD-accessibility range). All Q-Q plots except for “ON 10 nm” cross the diagonal line, demonstrating that both MPMS and FMMD-reconstructed distributions have a crossover. As noted earlier in the context of Fig. 9, the interpretation of these distribution data may differ from the literal distribution of magnetic moments. The splitting of the peaks is likely caused by internal energy levels arising from the magnetic anisotropy of the core.

Supplementary Note 2 : Unstable colloid mixture

To illustrate the undesired effects of unstable colloids and particle interactions, the physical model uses two particle types from the verification measurements in Supp. Fig. 1: Ocean Nanotech “ON 20 nm” and “ON 30 nm”. These particle types are known for their monodisperse core-diameter distributions. However, their zeta potential reported in the literature shows up to be relatively low [1]: $|\zeta_p| < 2$ mV. Therefore, the colloids that appear stable if kept separate, might flocculate and sediment once mixed. The mixture measurements for the pair “ON 20 nm” and “ON 30 nm” are shown in Supp. Fig. 2. The subfigures (a) and (b) show the real and imaginary parts of IMT $A_{1,2}$ as a function of LF field amplitude h_L. The subfigure (e) shows the LF-scan on the complex plane. The reconstructed distribution is shown in subfigure (c). Original samples were measured at 50 µL each (violet and red), then mixed to a final volume of 100 µL. The concentration of “ON 20 nm” and “ON 30 nm” was adjusted to 0.623 mg/ml and 1 mg/ml to equalize their contribution to the nonlinear signal. The sample was measured immediately after mixing “Mixture 50:50 (2 min)” (orange) and the next day “Mixture 50:50 (1 day)” (pink). The black curve “Est. Lin. Comb.” is calculated as a superposition of original constituents that would have been obtained if particles did not interact. Typically, 1 hour after mixing, the colloid becomes fully transparent, and sediment is observed.

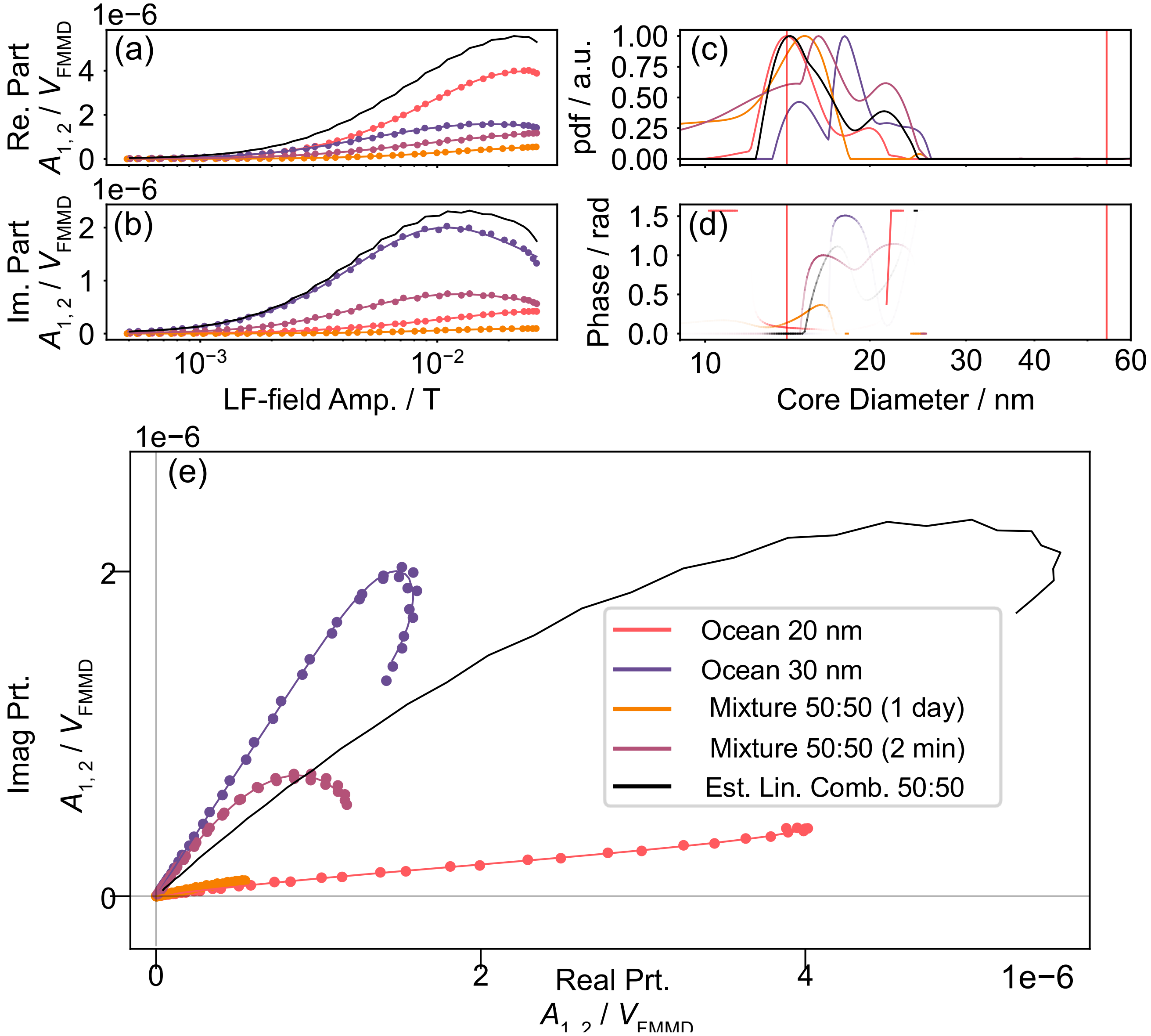

Supp. Fig. 2 | Unstable mixture and particle interactions. Dots: raw measurement. Solid line: back-reconstruction of the model. (a) Real part of IMT $A_{1,2}$ in LF-scan (b) Imaginary part of IMT $A_{1,2}$ in LF-scan (c) Normalized distribution of magnetic moments (d) Phase distribution of magnetic moments.

One can see that 2 minutes after mixing, the hook is still observed, whereas after one day, the response fully aligns with “ON 20 nm”. According to transmission electron microscopy images [1], these particles have a 1-2 nm thick shell. Using the

typical magnetite parameters, the “ON 20 nm” contains approximately $1.8 \cdot 10^{13}$ pcs. /ml and “ON 30 nm” has $0.87 \cdot 10^{13}$ pcs. /ml. Thus, the number of “ON 20 nm” particles is approximately two times larger, and after one day, 85% of these particles are in a bound state.

From the reconstructed distribution perspective, the actual distributions show the appearance of smaller magnetic moments, whereas the reconstruction from simulated superposition (black) does not extend to that range. This change can be attributed to particle-particle interactions upon particle clustering, from which the former can induce increased effective anisotropy[2] and the latter overall increases the anisotropy barrier $E = V_C \cdot K$, as the cluster of MNP increases the volume accordingly, overall alternating its signal in response to an AMF known for MFH and MPS measurements[3,4].

Supplementary Note 3: Resolution analysis

The resolution of the magnetic moment reconstruction can be achieved by estimating the information loss through successive solution of the forward and inverse problems in the Backus-Gilbert method[5]. Numerical Recipes [6] describes the implementation of this algorithm in detail. This method obtains for given linear model,

$$\mathbf{A}\vec{x} = \vec{\mathbf{b}} \tag{30}$$

a pseudo-inverse matrix $\mathbf{M} : \mathbf{E} \approx \mathbf{AM},$

$$\hat{\boldsymbol{x}} = \mathbf{M}\vec{\mathbf{b}} \tag{31}$$

optimized subject to the solution stability for given regularization parameter,

$$\min_{\mathbf{M}} \operatorname{std}([\hat{\mathbf{x}}]_i) = (\vec{\mathbf{q}}_i \cdot \mathbf{E} \cdot \vec{\mathbf{q}}_i)^{\mathbf{1/2}} \tag{32}$$

where $\vec{\mathbf{q}}_i = [\mathbf{M}]_i$ is a row of the pseudoinverse matrix.

The method’s output provides a resolution function $\delta(x, x')$, i.e., the function that shows cross-contribution of the original magnetic moment component x to the neighboring bin x'. Ideally, both components should coincide so $\delta(x, x')$= $\delta(x - x')$, but in reality, they smear, affecting the neighboring ones.

The first step is to obtain the spread matrix,

$$\mathbf{W}_{i,j,k} = \sum_l \left(\lg\left(\frac{x_i}{x_l}\right)\right)^2 \left(f(h_j, x_l) + n^{(1)}_{i,j,k}\right)\left(f(h_j, x_l) + n^{(1)}_{i,j,k}\right)\Delta_x \qquad \Delta_x = \lg\left(\frac{x_{i+1}}{x_i}\right) \tag{33}$$

The spread matrix $\mathbf{W}(x)$ characterizes the deviation from the delta-Dirac function for a given magnetic moment x. For each regularization parameter from the range and for each magnetic moment bin, the following least-squares problem is solved,

$$([\mathbf{W}]_i + \lambda\mathbf{E})\vec{\mathbf{y}}(x_i|\lambda) = \vec{\boldsymbol{R}}^{\mathrm{nrm}} \tag{34}$$

where $\vec{\boldsymbol{R}}^{\mathrm{nrm}}$ is a normalization vector obtained as follows,

$$[\vec{\boldsymbol{R}}^{\mathrm{nrm}}]_i = \sum_j f(h_i, x_j)\,\Delta_x \tag{35}$$

The row vectors of the pseudoinverse matrix can then be obtained,

$$\vec{\mathbf{q}}_i(\lambda) = \frac{\vec{\mathbf{y}}(x_i, \lambda)}{\vec{\boldsymbol{R}}^{\mathrm{nrm}} \cdot \vec{\mathbf{y}}(x_i, \lambda)} \tag{36}$$

The *resolution function* $\delta(x, x')$ is the scalar product of the forward and backward rows of the system matrices.

$$\delta(x_i, x_j) = \sum_k [\vec{\mathbf{q}}_i(\lambda)]_k f(h_k, x_j) \tag{37}$$

Supp. Fig. (a) shows the resolution matrices obtained for MPMS (left) and FMMD (right) at three noise levels and regularization parameters. The standard deviations for magnetic moment bins obtained using Fisher information are shown in Supp. Fig. (b). These show how the error in the measurement vector $\vec{\mathbf{b}}$ redistributes into the corresponding error in $\vec{\mathbf{x}}$. The effective width of the resolution matrix, as summarized in Supp. Fig. 4 and Supp. Fig. 5, is given by its diagonal elements.

The resolution matrix is characterized through the diagonality measure ρ_{diag} of the resolution matrix $\delta(x_i, x_j)$ defined as a weighted correlation factor between the row and column indexes of the matrix elements,

$$\begin{gathered} \mathrm{D}_{i,j} = \delta(x_i, x_j) \\ \rho_{\mathrm{diag}} = \frac{E[xy] - E[x]E[y]}{\sqrt{E[x^2] - (E[x])^2}\sqrt{E[y^2] - (E[y])^2}} \\ N = \sum_i \sum_j \mathrm{D}_{i,j} \\ E[x] = \sum_i \sum_j \frac{\mathrm{D}_{i,j}}{N} i \\ E[y] = \sum_i \sum_j \frac{\mathrm{D}_{i,j}}{N} j \\ E[x^2] = \sum_i \sum_j \frac{\mathrm{D}_{i,j}}{N} i^2 \\ E[y^2] = \sum_i \sum_j \frac{\mathrm{D}_{i,j}}{N} j^2 \end{gathered} \tag{38}$$

The diagonality measure ρ_{diag} for various regularization methods for both methods and noise levels are shown for both methods in Supp. Fig. (c).

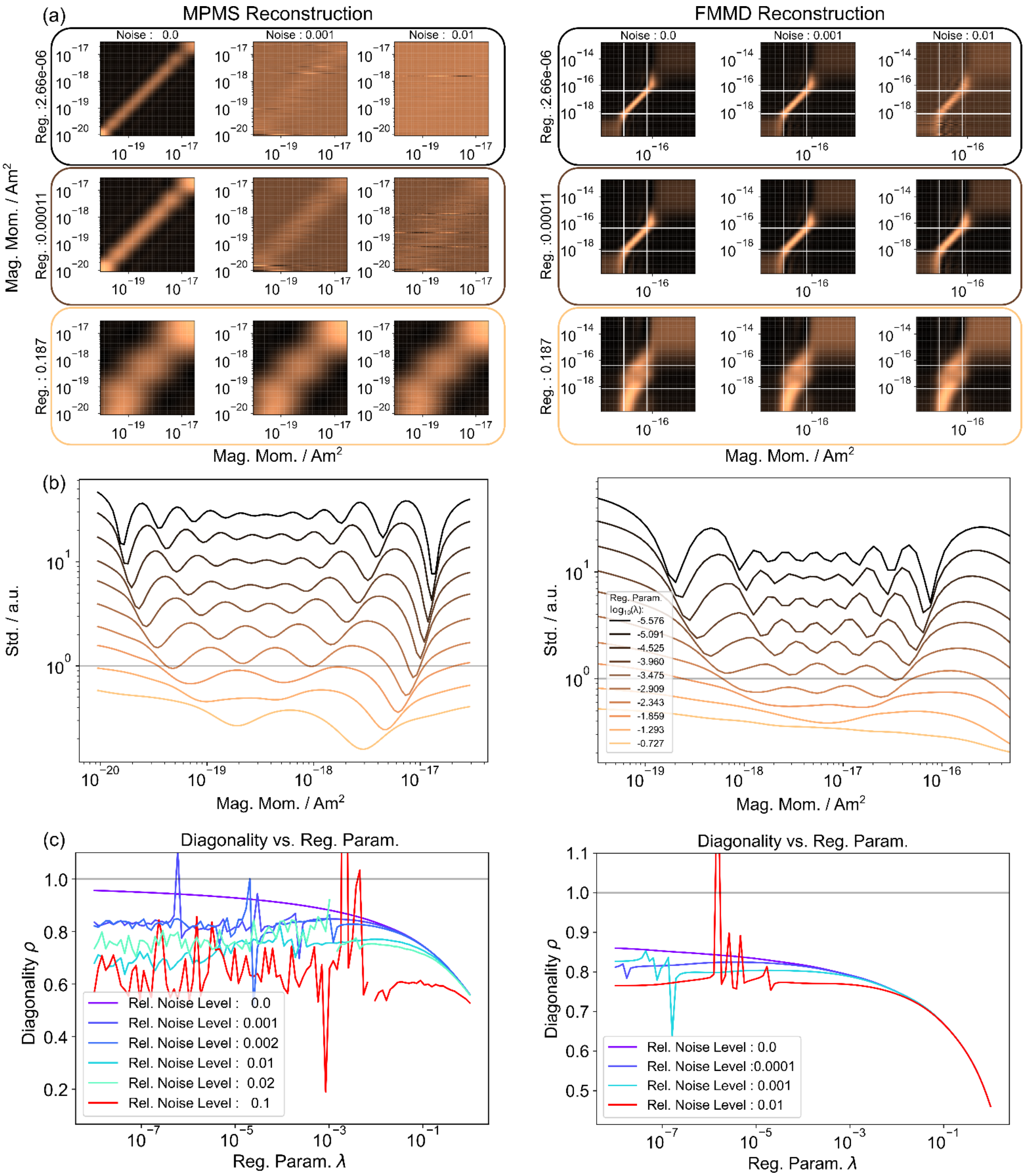

Supp. Fig. 3 | Resolution Analysis of the magnetic moment reconstruction. Left column: reconstruction form static magnetization. Right column: reconstruction from FMMD. (a) Resolution operator $\delta(x,x')$ calculated for static magnetization curve reconstruction for various noise levels and regularization parameters. (b) Standard deviations obtained by resolution analysis of the inverse operator for static magnetization measurement. Gray line: unity level of a normalized signal amplitude. (c) Diagonality obtained for the normally pre-disturbed measurement matrices imitating the noise in the data. The noise level shows the standard deviation of the disturbance σ_i^{mn}.

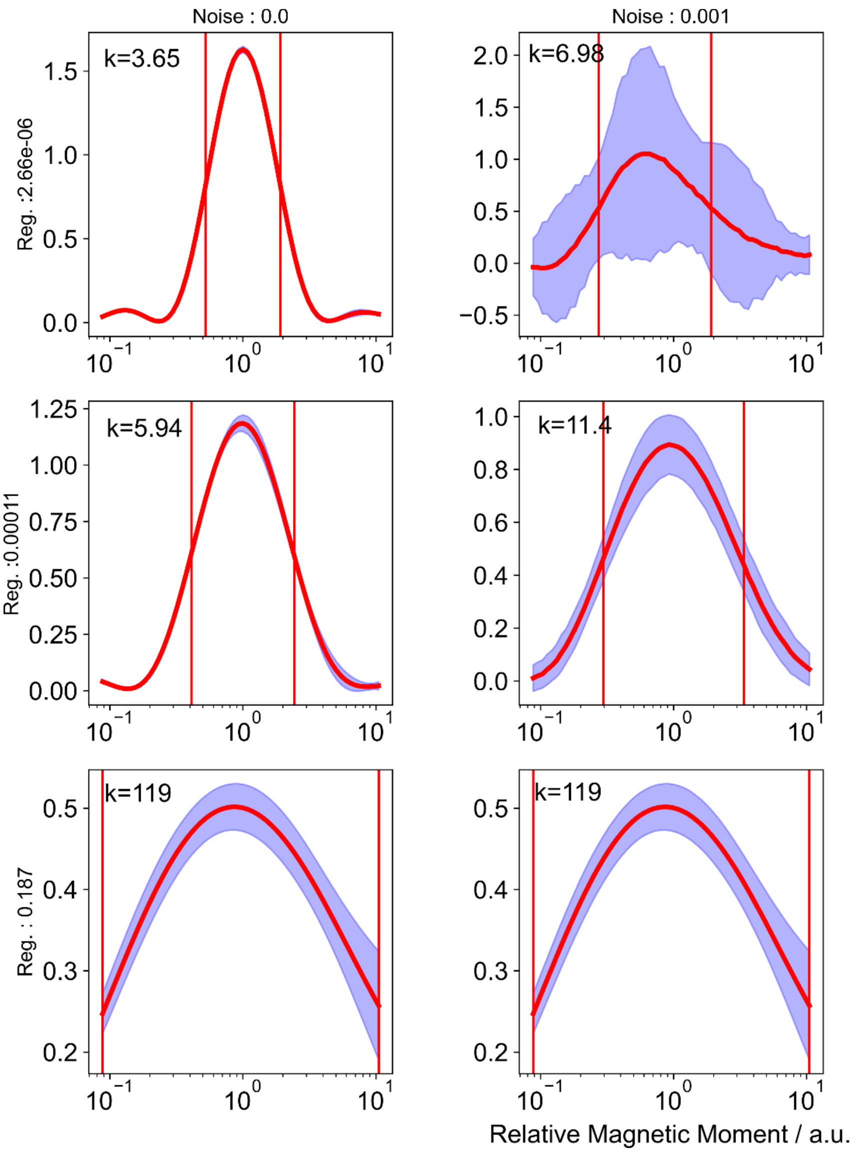

Supp. Fig. 4 | Resolution of MPMS reconstruction. Red line: average. Blue: standard deviation.

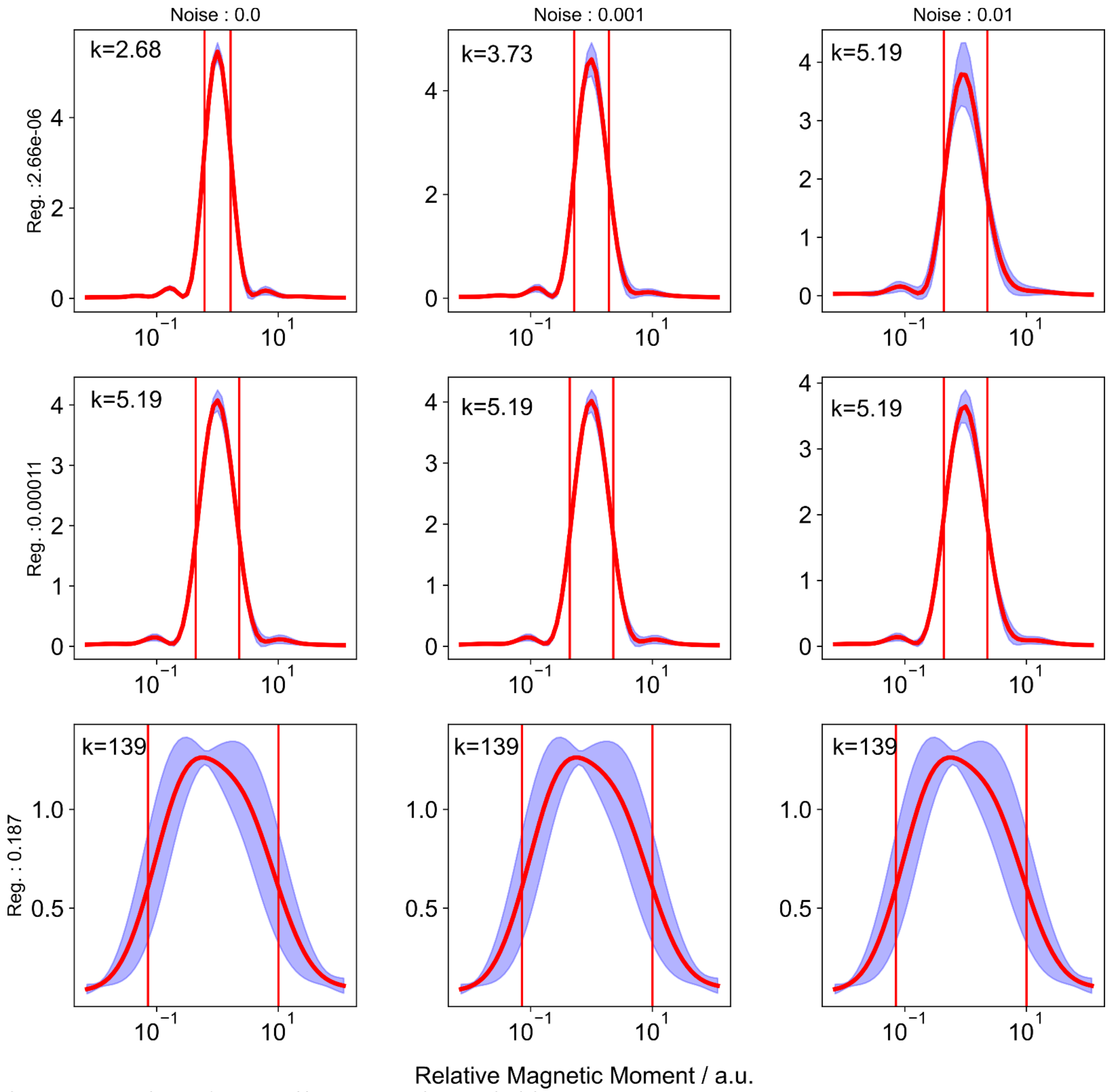

Supp. Fig. 5 | Resolution of FMMD. Red line: average. Blue: standard deviation.

Supp. Tab. 1 | Linear Decomposition of the signals

	**FMMD**		**MPMS**	
**Sample**	**Syn 50**	**Syn 70**	**Syn 50**	**Syn 70**
(1) Syn 50 nm	0.925	0.075	1.000	0.000
(2) Mix Syn50-Syn70 80:20	0.757	0.243	0.863	0.137
(3) Mix Syn50-Syn70 50:50	0.614	0.386	0.566	0.434
(4) Mix Syn50-Syn70 20:80	0.098	0.902	0.304	0.696
(5) Syn 70 nm	0.000	1.000	0.000	1.000

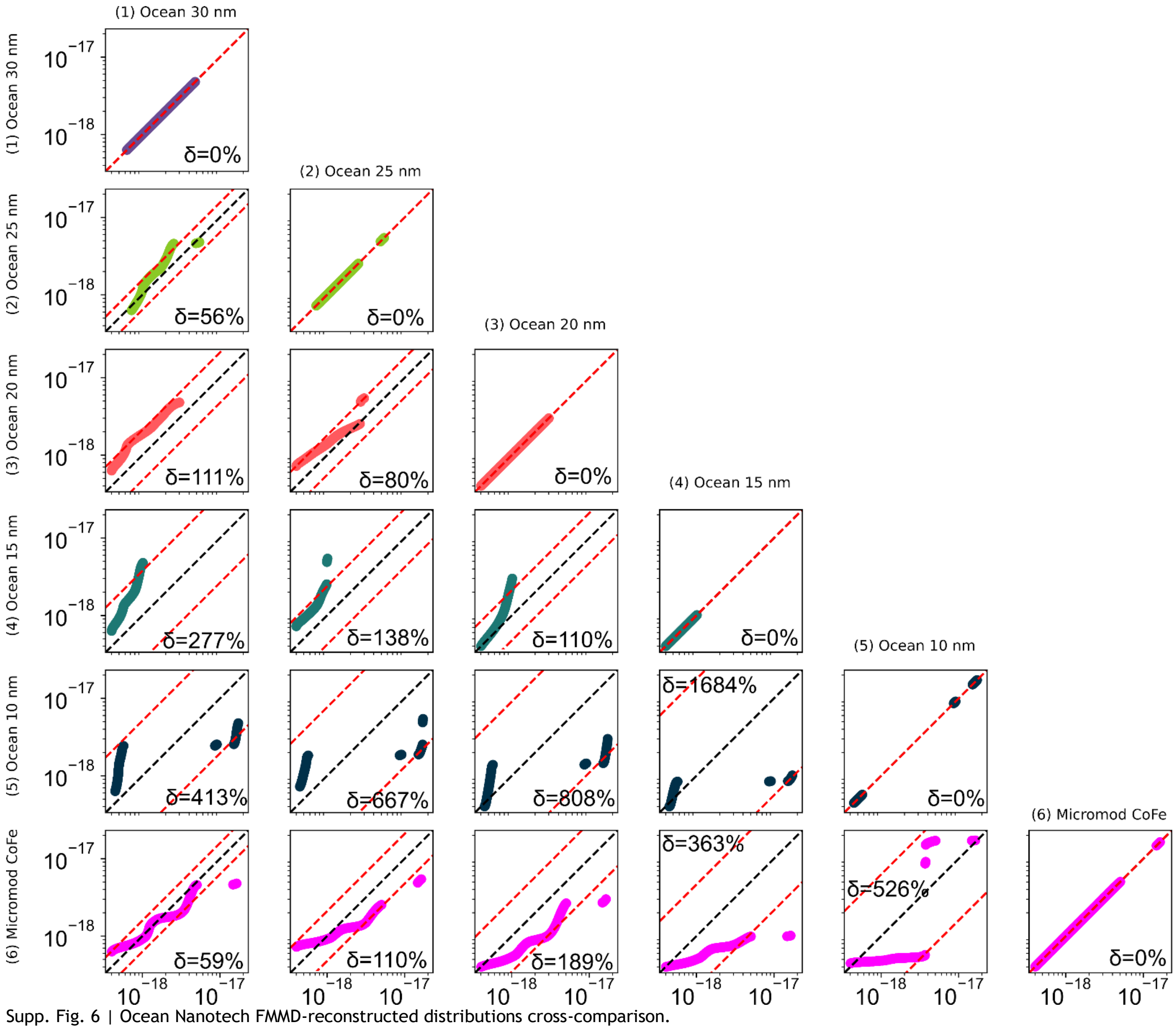

Supp. Fig. 6 | Ocean Nanotech FMMD-reconstructed distributions cross-comparison.

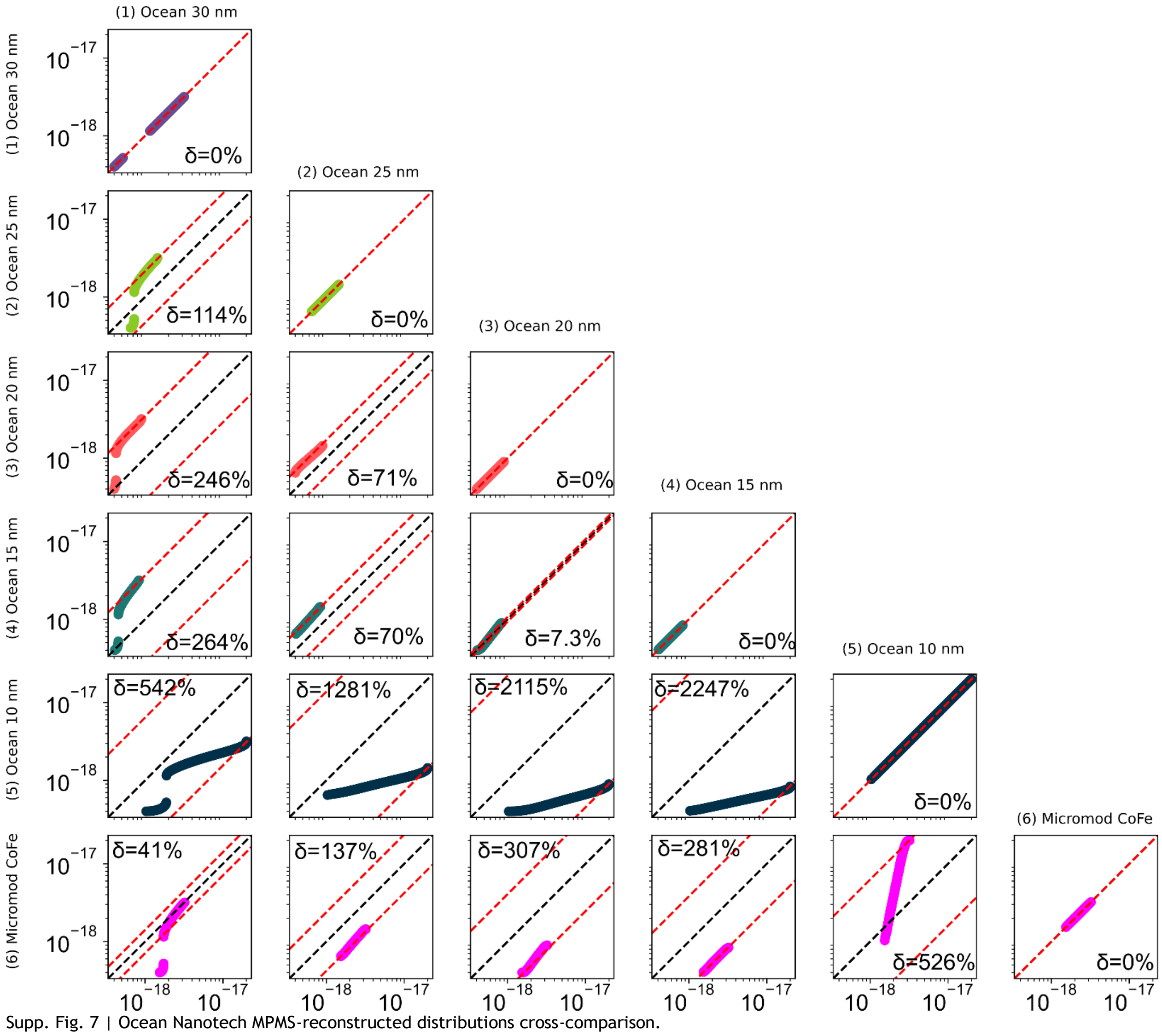

Supp. Fig. 7 | Ocean Nanotech MPMS-reconstructed distributions cross-comparison.

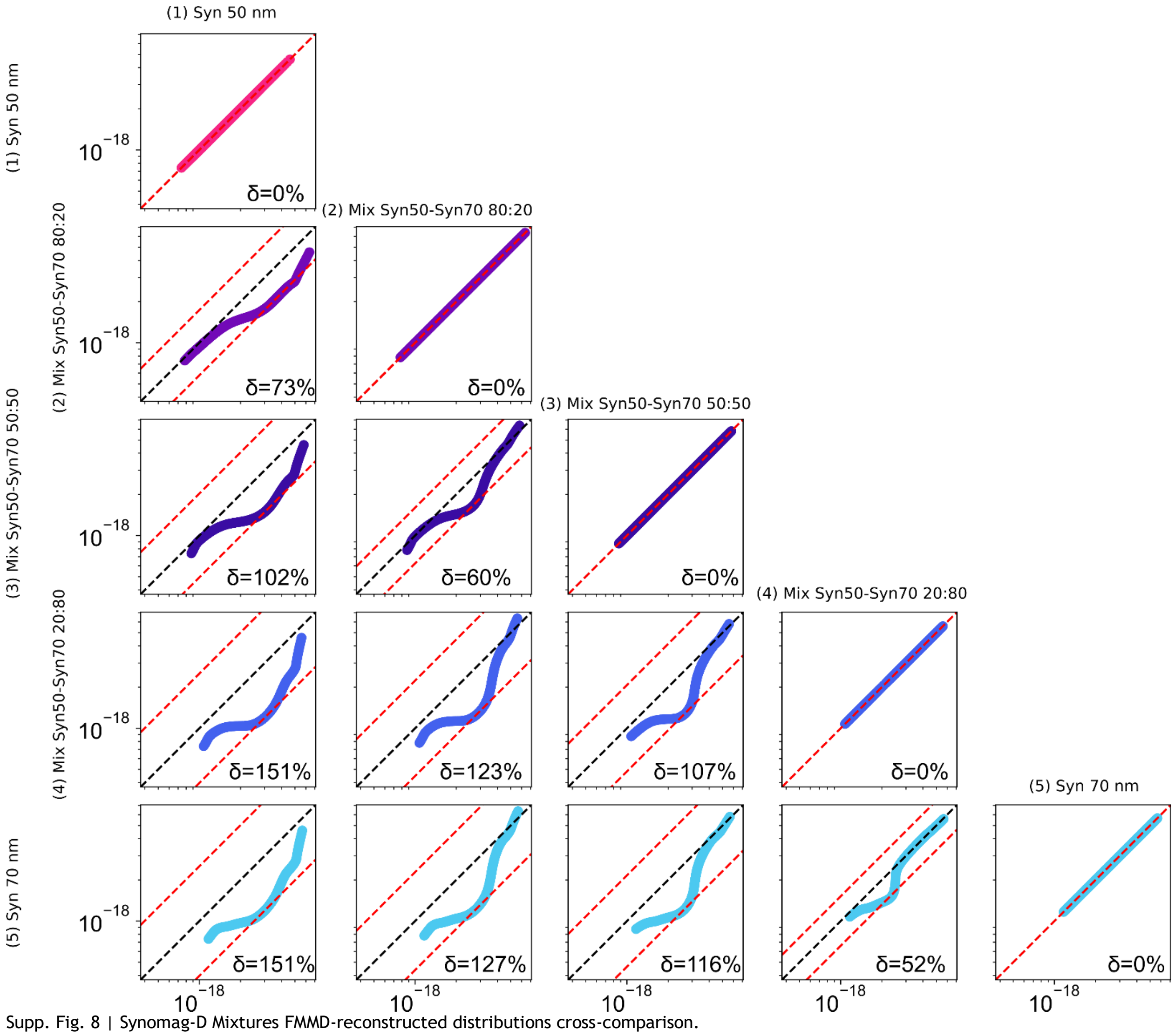

Supp. Fig. 8 | Synomag-D Mixtures FMMD-reconstructed distributions cross-comparison.

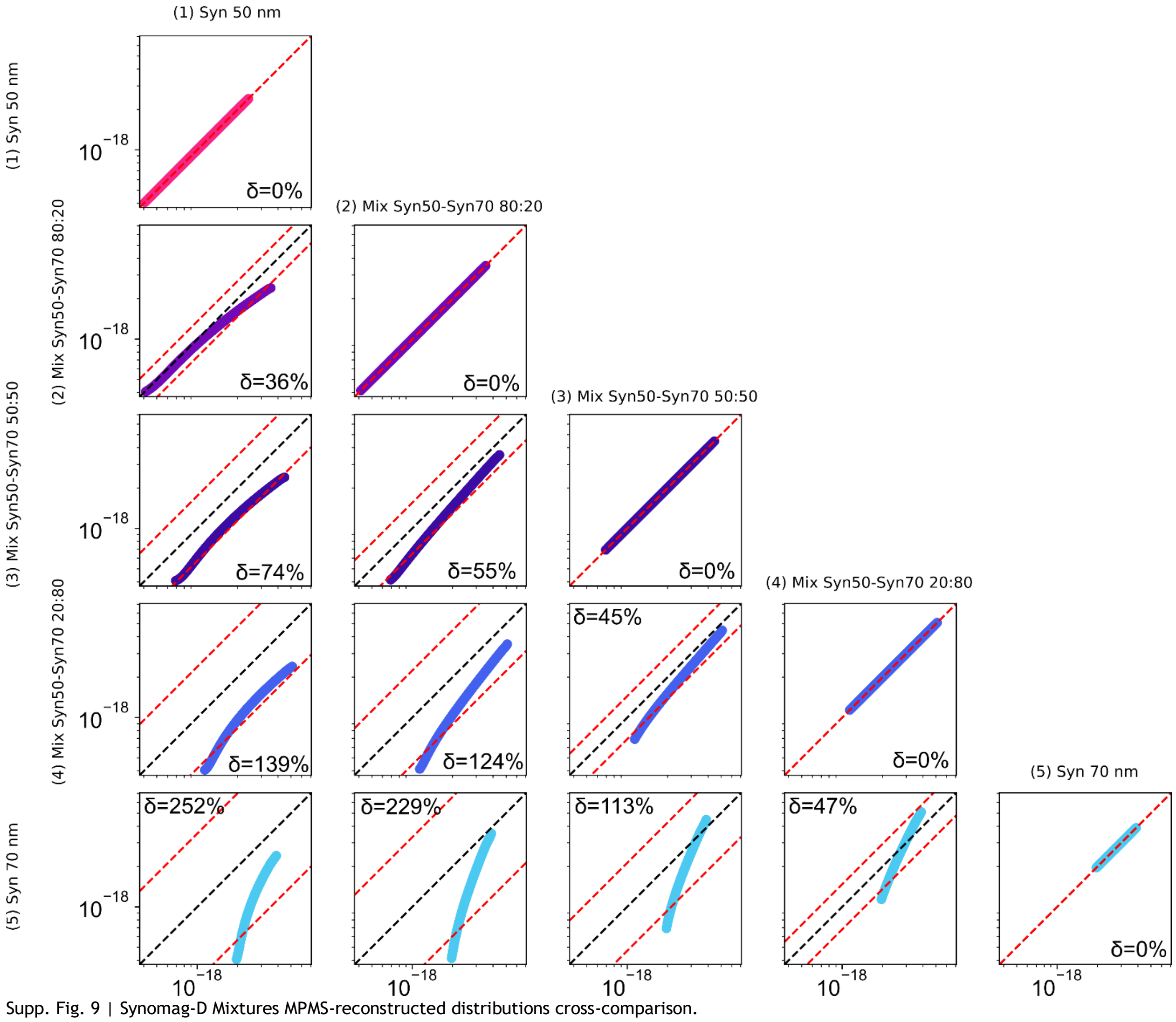

Supp. Fig. 9 | Synomag-D Mixtures MPMS-reconstructed distributions cross-comparison.

Supplementary References:

1. Wu, K. et al. Investigation of Commercial Iron Oxide Nanoparticles: Structural and Magnetic Property Characterization. ACS Omega 6, 6274–6283 (2021).
2. Krishnan, K. M. Biomedical Nanomagnetics: A Spin Through Possibilities in Imaging, Diagnostics, and Therapy. IEEE Trans. Magn. 46, 2523–2558 (2010).
3. Branquinho, L. C. et al. Effect of magnetic dipolar interactions on nanoparticle heating efficiency: Implications for cancer hyperthermia. Sci Rep 3, 2887 (2013).
4. Engelmann, U. M. et al. Magnetic Relaxation of Agglomerated and Immobilized Iron Oxide Nanoparticles for Hyperthermia and Imaging Applications. IEEE Magn. Lett. 9, 1–5 (2018).
5. Backus, G. & Gilbert, F. The Resolving Power of Gross Earth Data. Geophys J Int 16, 169–205 (1968).
6. Press, W. H. Numerical Recipes: The Art of Scientific Computing. (Cambridge University Press, Cambridge, UK, 2007).
