# Supplementary material for: Enhanced magnetic moment discrimination for multiplex nanoparticle quantification via dual-frequency nonlinearity probing
Source: Commun Eng. 2026 Jun 30;5:117. doi: 10.1038/s44172-026-00713-5 (PMC13319186; doi:10.1038/s44172-026-00713-5)
Supplement: Supplementary file 1 — Transparent Peer Review file [file 44172_2026_713_MOESM1_ESM.pdf]

nature portfolio

Peer Review File

Enhanced magnetic moment discrimination for multiplex nanoparticle quantification via dual-frequency nonlinearity probing

Corresponding Author: Professor Hans-Joachim Krause

This file contains all reviewer reports in order by version, followed by all author rebuttals in order by version.

Version 0:

Reviewer comments:

Reviewer #1

(Remarks to the Author)
This paper presents a method for separating magnetic signals from two-MNP mixtures, experimentally confirmed on a dual-frequency magnetic particle spectroscopy (MPS) platform. In the first part, the authors compare several existing models used to interpret higher harmonics of MNPs under driving magnetic field(s). They then integrate field modulation theory, magnetization model, and relaxation models to reconstruct the magnetic moment distribution of MNPs from harmonic signals. The ultimate goal is to enable multiplexed detection of different MNPs in mixtures for multiplexed assays in MPS or multi-tracer imaging in MPI.
Overall, the paper is well-organized, the theoretical framework is clearly explained, and the results are convincing. I recommend minor revision. My specific comments are as follows:

1. The motivation for separating the magnetic moments of different MNP types from a mixture with an ultimate goal of multiplxing is not sufficiently justified. Several studies have reported harmonic matrix methods for estimating the amounts of different MNPs in mixtures (e.g., DOIs: 10.1103/22cn-nw8t; 10.1088/0957-4484/21/45/455101; 10.1088/0022-3727/47/15/155001; 10.1118/1.3549762; 10.1016/j.jmmm.2018.11.021; 10.1088/1361-6463/acd0bd). The authors should clarify how their approach differs from and improves upon these existing methods.
2. Followup on comment #1, the authors should consider quantitatively comparing the multiplexing performance of their proposed method with those reported in the references above to highlight advantages or limitations.
3. Followup on comment #1, for the binary mixture multiplexing, an error of 8.9% appears relatively high. The claim that this method directly supports multiplexed assays or multi-tracer imaging applications is therefore not convincing. Instead, the authors may consider emphasizing broader implications — that this method for reconstructing the magnetic moment distribution of an MNP ensemble can enable accurate characterization of heterogeneity in particle size, composition, and magnetic anisotropy, informing synthesis and quality control. Such reconstructed distributions can also facilitate modeling of magnetization, relaxation, and heating behaviors, which are essential for applications in biosensing, magnetic hyperthermia, and magnetothermal stimulation. Furthermore, these insights can guide the design of optimized nanoparticles for imaging, diagnostics, and therapeutic systems requiring precise magnetic responses.
4. In Fig. 9(c), where reconstructed magnetic moment distributions are shown using the A1,2, it is unclear whether the size distribution of Syn50 and Syn70 MNPs was considered. Were these particles assumed to be monodisperse with nominal sizes of 50 and 70 nm, or was size variability incorporated into the analysis?

Reviewer #2

(Remarks to the Author)
This work focuses on addressing the long-standing challenge of limited resolution in reconstructing the magnetic moment distribution (MMD) of magnetic nanoparticles (MNPs) using conventional static magnetization curves. The authors propose a novel approach based on Frequency Mixing Magnetic Detection (FMMD) with dual-frequency excitation (10 Hz low frequency, 40.5 kHz high frequency), aiming to enhance magnetic moment discrimination for multiplex MNP applications. While the manuscript demonstrates rigorous theoretical analysis and extensive experimental work, it has notable shortcomings in practical application clarity, advantage differentiation, and innovation demonstration, which need to be addressed.

1. The manuscript mentions FMMD's potential for "multiplex MNP applications in biomedicine" but lacks concrete details to connect technical performance to real-world needs. The study focuses on MMD reconstruction and relaxation characterization of commercial MNPs under controlled laboratory conditions. However, it does not discuss how FMMD can be adapted to complex practical environments or address application-specific challenges.
2. The work does not fully articulate how FMMD outperforms well-established methods like Magnetic Particle Spectroscopy (MPS) or Dynamic Light Scattering (DLS), which may limit reader's understanding of its unique value. The authors mention that FMMD improves magnetic moment resolution, but they do not quantitatively compare key performance metrics with MPS. DLS is widely used to measure the hydrodynamic diameter of MNPs. However, the manuscript does not compare FMMD-derived particle diameters with DLS measurements, nor does it explain why FMMD is superior to DLS.
3. The core concept of dual-frequency excitation for MNP characterization has been explored in prior studies (e.g., dual-frequency MPS by Dr. Jian-ping Wang's group). The manuscript would benefit from more clearly distinguishing FMMD's innovations.

Some other minor issues:
1. "Finally, using AMF-driven magnetic particle spectroscopy (MPS)allows to localize an amount as small as ~100 pg of MNP with spatialresolution of 0.7 mm", MPS is generally considered to have no spatial resolution capability.
2. It is difficult to understand the trend of the lines and the meaning expressed in Fig. 3. Some grids need to be added or the number of curves in one figure should be reduced to make it clearer.
3. The experimental results in Fig. 8 are confusing. The results presented in c and d do not show a regular change in magnetic moment and particle size. Also, the results of FMMD and MPMS are different. In this case, the author's conclusion that the FMMD results are accurate drawn from the comparison of c and d is debatable. Moreover, the conclusion "we conclude that FMMD captures the correct order of average magnitude of magnetic moment evaluated in FMMD - accessible region for all particle types except 'ON 10 nm'" is not persuasive enough for the evaluation of a method. It is difficult to convince people that accurate results have been obtained. This experiment needs to be redesigned to obtain convincing conclusions .

Reviewer #3

(Remarks to the Author)
I co-reviewed this manuscript with one of the reviewers who provided the listed reports. This is part of the Communications Engineering initiative to facilitate training in peer review and to provide appropriate recognition for Early Career Researchers who co-review manuscripts.

Version 1:

Reviewer comments:

Reviewer #1

(Remarks to the Author)
The authors have resolved the issues. I suggest the acceptance of this paper.

Reviewer #2

(Remarks to the Author)
Fig. 8(c) and (d)：While Backus-Gilbert and drift analyses confirm theoretical resolution and signal stability, they remain indirect evidence and are insufficient to validate the reconstructed distributions in Fig. 8(c) and (d). I agree with the results of Fig.9 (c) and (d), because the distribution of peaks and the mixing ratios can roughly correspond to each other. However, Fig.8 lacks a ground truth comparison and cannot correspond to the provided particle sizes. There is no evidence that the multiple peaks in the FMMD results are more accurate rather than derived from overfitting. I suggest modifying or deleting this experiment.

Reviewer #3

(Remarks to the Author)
I co-reviewed this manuscript with one of the reviewers who provided the listed reports. This is part of the Communications Engineering initiative to facilitate training in peer review and to provide appropriate recognition for Early Career Researchers who co-review manuscripts.

Version 2:

Reviewer comments:

Reviewer #2

(Remarks to the Author)
All the suggested revisions have been incorporated, and the paper is now suitable for acceptance.

Reviewer #3

(Remarks to the Author)
I co-reviewed this manuscript with one of the reviewers who provided the listed reports. This is part of the Communications Engineering initiative to facilitate training in peer review and to provide appropriate recognition for Early Career Researchers who co-review manuscripts.

Open Access This Peer Review File is licensed under a Creative Commons Attribution 4.0 International License, which permits use, sharing, adaptation, distribution and reproduction in any medium or format, as long as you give appropriate credit to the original author(s) and the source, provide a link to the Creative Commons license, and indicate if changes were made.
In cases where reviewers are anonymous, credit should be given to 'Anonymous Referee' and the source.
The images or other third party material in this Peer Review File are included in the article's Creative Commons license, unless indicated otherwise in a credit line to the material. If material is not included in the article's Creative Commons license and your intended use is not permitted by statutory regulation or exceeds the permitted use, you will need to obtain permission directly from the copyright holder.
To view a copy of this license, visit https://creativecommons.org/licenses/by/4.0/

Reviewers' comments:

Reviewer #1 (Remarks to the Author):

This paper presents a method for separating magnetic signals from two-MNP mixtures, experimentally confirmed on a dual-frequency magnetic particle spectroscopy (MPS) platform.

In the first part, the authors compare several existing models used to interpret higher harmonics of MNPs under driving magnetic field(s).

They then integrate field modulation theory, magnetization model, and relaxation models to reconstruct the magnetic moment distribution of MNPs from harmonic signals.

The ultimate goal is to enable multiplexed detection of different MNPs in mixtures for multiplexed assays in MPS or multi-tracer imaging in MPI.

Overall, the paper is well-organized, the theoretical framework is clearly explained, and the results are convincing. I recommend minor revision. My specific comments are as follows:

1. The motivation for separating the magnetic moments of different MNP types from a mixture with an ultimate goal of multiplxing is not sufficiently justified. Several studies have reported harmonic matrix methods for estimating the amounts of different MNPs in mixtures (e.g., DOIs: 10.1103/22cn-nw8t; 10.1088/0957-4484/21/45/455101; 10.1088/0022-3727/47/15/155001; 10.1118/1.3549762; 10.1016/j.jmmm.2018.11.021; 10.1088/1361-6463/acd0bd). The authors should clarify how their approach differs from and improves upon these existing methods.

Thank you for your kind response and for the literature suggestions. We included several references mentioning their value as a practical conventional implementation of multicontrasting assays. In our article, we are trying to figure out the method to deal with difficulties that arise in the conventional multicontrasting methods, such as mentioned in these articles: *a-prior* measurement of initial mixture constituents is done and later used to linearly decompose the signal of a mixture. The problem arises when particles in the mixture interact with each other, and due to dipole-dipole interactions, their magnetic response deviates from just the (expected) linear combination of the signals. To illustrate this, we now also provide additional experimental data in the supplementary materials. In this additional experiment, we mixed two Ocean Nanotech particle samples that individually appear as stable colloids, but start to sediment due to interaction effects once they get mixed. The magnetic response of their mixture does not directly match the combination of their individual linear signals. Our method, as exemplified in this case, shows the appearance of particles with smaller magnetic moments in the distribution directly from measurement. In fact, these are presumably not new particles, but the contributions of clustered particles that create additional energy barriers that effectively reduce their magnetic moments in response to the dual-frequency excitation. Thus, our new method allows to identify the occurrence of particle interactions. From a practical perspective, one could use conventional methods to identify this particle interactions, too, for instance by observing the deviation of the response from the expected linear combination. However, our approach enables direct quantification of the interaction effect and distinction from other effects that affect the response amplitude, such as an amplitude reduction due to viscosity change. We assume that the viscosity alteration does not affect the nonlinear properties of the response, in particular, the peak position on the LF-scan.

Thanks to your comments, we addressed and emphasized these differences in the abstract " **The binary mixture ratio was quantified with 8.9% deviation, without any prior information about the initial constituents differing by a factor of 3 in their average magnetic moments, potentially allowing the accommodation of three independent contrast channels for multiplex MNP applications as well as qualitatively probing magnetic interaction effects**" and in the results section as "**The mixture samples (#2, #3, #4) are reconstructed as bimodally distributed without any prior knowledge of the original constituents, meaning that the FMMD measurements for #1 and #5 were not used in the reconstruction at all.** "

2. Followup on comment #1, the authors should consider quantitatively comparing the multiplexing performance of their proposed method with those reported in the references above to highlight advantages or limitations.

Other groups report relative errors varying from 2.3% [Tu et al., J Phys D (2014) doi: 10.1088/0022-3727/47/15/155001] up to 10% [Viereck et al., JMMM (2019) doi: 10.1016/j.jmmm.2018.11.021]. For comparability, we have applied the same linear decomposition methods to both FMMD and MPMS measurements. The estimated concentration estimation error does not exceed 11% for FMMD and 10% for MPMS mixtures. The results are now summarized and included in the paper's supplementary material to support clarity and transparency of our argumentation. We attribute these deviations to the possibility that some particles in the colloid may interact with one another, altering their magnetic response. As a rule of thumb, interactions may reduce the observable magnetic moment, as neighbouring particles generate magnetic fields larger than the external field. These fields, in turn, increase the energy barrier required for particle remagnetization. We have added a paragraph dedicated to this explanation in the Discussion section, starting with "**Currently, as reported in the literature, mixture analysis is conducted by decomposing the measured response into a linear combination** …"

3. Followup on comment #1, for the binary mixture multiplexing, an error of 8.9% appears relatively high. The claim that this method directly supports multiplexed assays or multi-tracer imaging applications is therefore not convincing. Instead, the authors may consider emphasizing broader implications — that this method for reconstructing the magnetic moment distribution of an MNP ensemble can enable accurate characterization of heterogeneity in particle size, composition, and magnetic anisotropy, informing synthesis and quality control. Such reconstructed distributions can also facilitate modeling of magnetization, relaxation, and heating behaviors, which are essential for applications in biosensing, magnetic hyperthermia, and magnetothermal stimulation. Furthermore, these insights can guide the design of optimized nanoparticles for imaging, diagnostics, and therapeutic systems requiring precise magnetic responses.

Thank you very much for pointing out the relative error of our reconstruction. It is indeed a large error for the final practical application. However, it falls within the range of typical errors reported in the literature, including the references you kindly provided (see answer to Q2). Fully accepting this error, though it is debatable, we attribute it to two potential sources stemming from the quality of the particles used. The first reason is purely mathematical. To achieve better results, the measurement vectors should ideally be orthogonal in a mathematical sense. However, as you can observe in the Fig. 8, these samples are far from being ideally orthogonal. The second reason is the stability of the particles. The linear decomposition implicitly assumes that mixing does not affect the magnetic properties of the whole sample, which is treated as a linear combination of the initial constituents.

Nevertheless, one cannot exclude the possibility that some part of the sample is unstable, as we have shown for a mixture of Ocean Nanotech particles, where strong sedimentation was observed,

presumably due to magnetic interactions. We attribute the 10% error present in both methods, FMMD and MPMS, to particle-particle interactions, especially since it is approximately identical for the two independent measurement techniques. Moreover, the evaluation of this deviation in real samples can be used to indirectly assess particle interactions. In addition, we revised our discussion of the method's relevance in line with your suggestions in the paragraph starting with "**In contrast to the conventional linear decomposition approach, our method can differentiate bimodal sample distributions without** …"

4. In Fig. 9(c), where reconstructed magnetic moment distributions are shown using the A1,2, it is unclear whether the size distribution of Syn50 and Syn70 MNPs was considered. Were these particles assumed to be monodisperse with nominal sizes of 50 and 70 nm, or was size variability incorporated into the analysis?

Thank you for pointing out this uncertainty in our storyline, which is very important for the whole work. Neither any particular model of magnetic moment (core size) distribution (i.e., log-normal) was considered, nor any assumption about monodispersity of Syn 50 nm and Syn 70 nm was made. The sizes mentioned in the names of these samples are their commercial names, corresponding to their hydrodynamic diameters: dextran shells of two distinct shell thicknesses with outer diameters of 50 nm and 70 nm, are used. The magnetic properties determined in our reconstruction depend on the core size, which lies between 18 nm and 24 nm for these particles. Regarding monodispersity of the samples, as you can observe in the Fig. 8, the samples often do not appear as monodisperse on the magnetic moment distribution. We have selected particular batches of Syn 50 nm and Syn 70 nm from several dozen other batches because their distributions appeared the narrowest in both FMMD and MPMS measurements. A narrow distribution does not, per se, guarantee monodispersity, but it does indicate good conformity with the selected magnetization model. We mention and now emphasise this in the sentence: "**The batches of these MNP samples ("Syn-D 50 nm" and "Syn-D 70 nm") were selected, as they demonstrated the narrowest and non-overlapping magnetic moment distribution reconstructed from FMMD measurement (Fig. 9, c) in contrast to the samples previously presented in Fig. 8 (c). The numbers in their titles denote their hydrodynamic sizes provided by the manufacturer and do not reflect their magnetic core sizes, which determine their magnetic properties.** ". In another position in the text, the formulation was adjusted to emphasise this reconstruction independence: "**The mixture samples (#2, #3, #4) are reconstructed as bimodally distributed without any prior knowledge of the original constituents, meaning that the FMMD measurements for #1 and #5 were not used in the reconstruction at all.**"

Reviewer #2 (Remarks to the Author):

This work focuses on addressing the long-standing challenge of limited resolution in reconstructing the magnetic moment distribution (MMD) of magnetic nanoparticles (MNPs) using conventional static magnetization curves. The authors propose a novel approach based on Frequency Mixing Magnetic Detection (FMMD) with dual-frequency excitation (10 Hz low frequency, 40.5 kHz high frequency), aiming to enhance magnetic moment discrimination for multiplex MNP applications. While the manuscript demonstrates rigorous theoretical analysis and extensive experimental work, it has notable shortcomings in practical application clarity, advantage differentiation, and innovation demonstration, which need to be addressed.

1. The manuscript mentions FMMD's potential for "multiplex MNP applications in biomedicine" but lacks concrete details to connect technical performance to real-world needs.

Thank you very much for identifying this critical gap in the storyline of our work. The importance and the place of multicontrast measurement in practice should be addressed. We now emphasize the connection between practical implication and the multicontrasting by adding the sentence "**In the future, such a multicontrasting capability would, for instance, enable simultaneous remote viscosity/temperature measurement, as it provides two or more fully separated telemetry channels and magnetic moment (i.e., MNP core size) analysis based on MNP signal-generating capabilities in different contexts**" into the introduction while trying to keep the introduction understandable for broader audiences.

As Reviewer #1 also mentioned, multiplex particle detection is a far-fetched goal, whereas our technique addresses the practical problems known to professionals involved in magnetic nanoparticle synthesis and characterization. The current problem in magnetic nanoparticle characterization is a needle-in-haystack problem: a minor contamination of the sample with particles of larger core size can cause a pronounced change in the nonlinear magnetization response. We emphasise these challenges in the introduction section by adding "**However, this problem requires a method that yields the so-called core size distribution, which translates in magnetic measurements into a magnetic moment distribution (MMD) for the entire sample particle population. Reconstruction of MMD can be challenging due to a minor contamination of the sample with particles of larger core sizes that can significantly alter the nonlinear response, which is dominated by large particles 20. At the same time, the methods of direct core size evaluation (such as TEM) do not allow nondestructive sampling of the whole population. In contrast, indirect methods (such as static magnetization measurement) lack sufficient resolution to precisely distinguish sizes, as explained below.".**

As we now (thanks to adding supplementing materials in answering question 1-3 from reviewer #1, see above) include more details on interaction-driven analysis capabilities of FMMD, we added an outlooking and concluding remark tot the end of the introduction: "**From this, we conclude that FMMD enables non-destructive, direct quantification of the magnetic moment distribution in unknown MNP samples without requiring prior assumptions on the sample properties. For a well-characterized MNP system, the framework could potentially provide a systematic approach to qualitatively detect the onset of interparticle magnetic interactions. Collectively, these capabilities would offer a foundation for enhancing quality control and assurance in MNP synthesis, characterization, and reproducibility assessment."** This paragraph directly links to newly added paragraph in the discussion, reading "**Furthermore, the method may allow for assessing well-characterized MNP systems for the onset of particle-particle interaction effects (as discussed and shown for the combination of 20 and 30 nm ON particles above) thus serving as a convenient means for rapid, non-destructive quality control and assurance of larg(e)r scale MNP production or individual characterization for research application as well as scale-up synthesis for commercialization processes.**"

The study focuses on MMD reconstruction and relaxation characterization of commercial MNPs under controlled laboratory conditions. However, it does not discuss how FMMD can be adapted to complex practical environments or address application-specific challenges.

Thank you very much for this point. We fully share the view that the characterization technique should be applicable to the practical environment, and we tried to provide as much information as possible without exceeding the limitations of this paper. For instance, we discuss how the ambient viscosity affects the signal in the results. We provide a procedure for estimating the hydrodynamic size from these measurements. Motivated by another remark of yours, we performed an additional experiment, demonstrating how particle-particle interactions appear in the reconstructed magnetic moment distributions, and added its value for quality control and assurance (see above). Moreover, we

dedicated a large part of the Materials and Methods to this issue, providing means to evaluate the stability and quality of synchronous measurements, explicitly showing whether there is drift in the measurements and how reliable the results are. In particular, we emphasise the use of the Rice-Nakagami distribution for this evaluation and the determination of a long-term drift coefficient. Such measures were not introduced in the context of MPS before. Finally, we provide a detailed description of the measurement setup, which is, in fact, an integrated, fully portable device. We tried to provide all the experimental details necessary for reproducing the method. Addressing application-specific challenges has always been our aim: together with the German Technical Relief Organization (THW), we developed a magnetic reader based on the FMMD principle to be used under extremely harsh field conditions, in particular for detecting Cholera to ensure drinking water safety during catastrophic flooding events [Achtsnicht et al., PLOS ONE 14, e0219356 (2019). doi: 10.1371/journal.pone.0219356]. Also, other groups, e.g., in Minneapolis (USA) offer similar technology, reporting handheld devices for MPS and FMMD measurements under flexible conditions [K. Wu, V. K. Chugh, A. Di Girolamo, J. Liu, R. Saha, D. Su, V. D. Krishna, A. Nair, W. Davies, Y. A. Wang, M. C.-J. Cheeran, J.-P. Wang, *ACS Appl. Mater. Interfaces* 2021, **13**, 7966], proving its potential applicability from benchtop in research labs to bedside in clinical settings.

2. The work does not fully articulate how FMMD outperforms well-established methods like Magnetic Particle Spectroscopy (MPS) or Dynamic Light Scattering (DLS), which may limit reader's understanding of its unique value.

Thank you very much for pointing out this uncertainty in our text. First, we would like to emphasise that FMMD is a subtype of MPS, just appended with one additional high-frequency component. Our technique does not compete with, and cannot replace Dynamic Light Scattering, as it measures magnetic properties of the nanoparticles and not their hydrodynamic diameter (like DLS). The latter can be estimated indirectly from FMMD data, but in this work, this estimation is done from AC susceptometry data for self-evaluation purpose. The $CoFe_2O_4$ particles are manufactured using the same technology as the Synomag-D 70 nm particles but with altered core material, and the proposed procedure of indirect estimation of its hydrodynamic diameter can be a useful tool to complement other characterization measurements.

The main difference between single-frequency MPS and dual-frequency MPS (FMMD) is that the latter allows separation of the amplitude and rate-related origins of nonlinearity. While we discuss the difference in the MPS/FMMD response in the theoretical part, we also suggest distinguishing the origins of the nonlinear signals, which can be caused by finite magnetizability and by a finite rate of magnetic response. Dual-frequency excitation creates an envelope in the frequency domain whose symmetries directly reveal the nonlinearity's origin (Fig. 1), whereas in single-frequency excitation, the signal's envelope self-interferes, and the nonlinearity's origin cannot be readily identified. This peculiarity is depicted separately in Fig. 3, which we modified according to your suggestion (see question 5 below). The dynamic rate-related nonlinearity's origin results in the redistribution of curvature peaks along the ascending and descending pathways, leading to the superimposition of both effects in MPS. Thus, the interpretation of the MPS response is obstructed, while it can be demodulated and distinctly interpreted as shown in this work for FMMD.

The authors mention that FMMD improves magnetic moment resolution, but they do not quantitatively compare key performance metrics with MPS.

Thank you very much for pointing out this deficiency in our work. First of all, to avoid any misunderstanding, we would like to emphasise that when we talk about magnetic moment resolution, we mean the resolution in the parameter space of the magnetization model. In other words, we mean

the resolution of the core-size reconstruction, not the experimentally accessible magnetic moment of the whole sample that is actually measured. The reconstructed magnetic moment distribution is a partition function that shows how the experimental magnetic moment is distributed among particles of different core diameters, or, in physical terms, among particles of different magnetic moments.

We guess that this misunderstanding takes place here as well, as the typical performance metrics of MPS include spectral morphological characteristics such as R3/R5 ratio, there is a magnetic moment resolution or magnetic moment sensitivity expressed in terms of $\mathrm{Am}^2/\sqrt{\mathrm{Hz}}$. We provide such a metric in the first paragraph of the results section, and this metric is directly comparable to that of MPS (giving $7.37 \cdot 10^{-14}\ \mathrm{Am}^2/\sqrt{\mathrm{Hz}}$ for our FMMD device). But this is not the improvement in magnetic moment resolution mentioned in the abstract.

To the best of our knowledge, the only works in which such magnetic moment distribution reconstructions are conducted are those of Berkov 2001, Bender et al. 2018, Sun et al. 2021 and Sun et al. 2024. In these works, however, no resolution analysis was conducted, and one can judge their resolution capabilities only indirectly. We would be grateful if you could provide an example of such a magnetic moment resolution metric for MPS, which we would be happy to use for future interpretation and benchmarking of our findings.

Nevertheless, to provide the clarification needed in response to your comment, we present a complete, mathematically justified resolution analysis in the supplementary materials using the Backus-Gilbert method. The idea behind this method is that the information loss is estimated by solving the forward and inverse problems iteratively. As a result, a resolution matrix can be obtained that directly shows the width of the peak in the magnetic moment distribution. The Full Width at Half Maximum (FWHM) resolution factor is k = 2.68 for FMMD and 3.65 for MPMS in the noiseless case. This factor means that we can resolve two constituents differing by a factor of k. With increasing noise, the difference in the systems' resolution factors becomes even more pronounced, indicating that FMMD is less susceptible to noise, although it has a smaller range of reconstructible magnetic moments.

DLS is widely used to measure the hydrodynamic diameter of MNPs. However, the manuscript does not compare FMMD-derived particle diameters with DLS measurements, nor does it explain why FMMD is superior to DLS.

We do not claim that FMMD is superior to DLS measurement. FMMD magnetically measures the core size, whereas DLS optically measures the hydrodynamic size. However, theoretically, FMMD allows the hydrodynamic size to be indirectly determined by evaluating the imaginary part of the response under varying viscosity conditions. In this work, an estimation of the hydrodynamic size is performed using AC susceptometry data for self-evaluation.

3. The core concept of dual-frequency excitation for MNP characterization has been explored in prior studies (e.g., dual-frequency MPS by Dr. Jian-ping Wang's group). The manuscript would benefit from more clearly distinguishing FMMD's innovations.

Yes, this is absolutely true. Actually, we already acknowledge the work of Dr. Jian-ping Wang, among others, in references 38 and 39. In fact, dual-frequency assessment of nonlinear systems has existed for more than a century. In the context of magnetic particle evaluation, the FMMD technique is an alias of the dual-frequency MPS technique. In our work, we emphasize the advantage of dual-frequency nonlinear probing with excitation amplitude variation, which is an innovation in itself, as we

are not aware that, e.g., the group of Dr. Jiang-Ping Wang is using this as of now. We aim to improve understanding of theoretical frameworks for signal reconstruction, i.e., their interpretation for sample characterization, in hopes of sparking further research from the MPS community in this field.

Some other minor issues:

4. "Finally, using AMF-driven magnetic particle spectroscopy (MPS)allows to localize an amount as small as ~100 pg of MNP with spatial resolution of 0.7 mm", MPS is generally considered to have no spatial resolution capability.

Thank you very much for pointing out this confusion. Indeed, MPS is a 0D technique, and one should say that Magnetic Particle Imaging achieves spatial resolution through the MPS readout principle. We have changed the sentence accordingly: "**Finally, using the AMF-driven magnetic particle spectroscopy (MPS) read-out principle, Magnetic Particle Imaging (MPI) allows localization of as little as ~100 pg of MNP with a spatial resolution of 0.7 "mm"**"

5. It is difficult to understand the trend of the lines and the meaning expressed in Fig. 3. Some grids need to be added or the number of curves in one figure should be reduced to make it clearer.

We would like to especially thank you for this recommendation, as we had two variants of this figure and did not know which would be more understandable. We completely modified the figure, removing the 3D appearance, to convey the main idea of extending the adiabatic model with a dynamic delay element. The paragraph describing the figure was entirely reworked: "**Fig. 3 assists in understanding these effects of amplitude-driven phase modulation on the spectral symmetries.**"

6. The experimental results in Fig. 8 are confusing. The results presented in c and d do not show a regular change in magnetic moment and particle size.

Thank you for paying attention to this critical comparison. Indeed, the FMMD-based reconstructions do not show regular changes in the magnetic moment distribution, whereas the MPMS do so. This comparison is provided here deliberately to highlight a key methodological issue: the static magnetization model of the Langevin curve should be used with care, as it does not account for anisotropy effects. The latter appear in the reconstructions as artefact peaks. Ocean Nanotech particles have been extensively investigated by TEM and are known for their monodisperse core sizes.

Nevertheless, in liquid colloids, they do not follow a pure Langevin curve. We emphasise this in the text below Fig. 2. We experimentally demonstrate the resolving capability of our reconstruction, shown in Fig. 9. Thus, the observed spurious peaks are not a resolution artifact of reconstruction, but an indication that the Langevin curve model does not consider anisotropy, as mentioned in the context of the reconstruction discussion. In contrast, MPMS-based reconstruction does not show these spurious peaks, as the deconvolution kernel, namely the Langevin curve, is smoother than the deconvolution kernel of FMMD reconstruction. The smoothening kernel merges the peaks together while keeping the first statistical moment of the distribution. To support our argument, we have conducted a resolution analysis of the reconstruction operators for MPMS and FMMD using the Backus-Gilbert method. This method allows the estimation of the width of the reconstructable peak for a given regularization parameter and measurement noise level. In the supplementary materials, the whole procedure and a visual depiction of the results are presented. The subsequent experiment with Syn50 nm and Syn 70 nm was presented to show that we achieve this resolution experimentally by mixing two samples that appear narrow in the magnetic moment distribution space.

Also, the results of FMMD and MPMS are different. In this case, the author's conclusion that the FMMD results are accurate drawn from the comparison of c and d is debatable. Moreover, the

conclusion “we conclude that FMMD captures the correct order of average magnitude of magnetic moment evaluated in FMMD - accessible region for all particle types except ‘ON 10 nm’” is not persuasive enough for the evaluation of a method. It is difficult to convince people that accurate results have been obtained. This experiment needs to be redesigned to obtain convincing conclusions .

Please note that the statement “**we conclude that FMMD captures the correct order of average magnitude of magnetic moment evaluated in FMMD**” is not based on shape-wise comparison made in quantile-quantile fashion, as shown in subfigures (e), but on the **average** magnetic moment calculated from the distributions, which is shape-independent. So, the claim is based on the comparison displayed in Fig. 5. (l). Comparison by the first statistical moment is done deliberately because such a comparison at the level of statistical moments allows us to avoid discussion of the exact shape or distribution model, as the distribution average should remain invariant within a given magnetization model. To support the claim that the obtained distributions are correct, we provide a resolution analysis in the supplementary materials using the Backus-Gilbert method. With this experimental redesign, we show that the resolution in magnetic moment space is indeed improved. We added a paragraph dedicated to this issue in the results section “**The resolution analysis, conducted using the Backus-Gilbert method [54] (see “Resolution analysis” in supplementary materials), for both FMMD and MPMS reconstructions yielded …”**

Reviewer #3 (Remarks to the Author):

I co-reviewed this manuscript with one of the reviewers who provided the listed reports. This is part of the Communications Engineering initiative to facilitate training in peer review and to provide appropriate recognition for Early Career Researchers who co-review manuscripts.

Thank you very much for putting in the extra work – we appreciate your co-operative support!

Reviewers' comments:

Reviewer #1 (Remarks to the Author):

The authors have resolved the issues. I suggest the acceptance of this paper.

Thank you very much for your time!

Reviewer #2 (Remarks to the Author):

Fig. 8(c) and (d)： While Backus-Gilbert and drift analyses confirm theoretical resolution and signal stability, they remain indirect evidence and are insufficient to validate the reconstructed distributions in Fig. 8(c) and (d). I agree with the results of Fig.9 (c) and (d), because the distribution of peaks and the mixing ratios can roughly correspond to each other. However, Fig.8 lacks a ground truth comparison and cannot correspond to the provided particle sizes. There is no evidence that the multiple peaks in the FMMD results are more accurate rather than derived from overfitting. I suggest modifying or deleting this experiment.

Thank you very much for your contribution and fruitful suggestions! We fully share your view that one should be very careful with the interpretation of any model-based curve-fitting results. Therefore, we fully followed your recommendation and removed Fig. 8 from the text. As we do not want to withhold our measurement data of widely used particles, and to avoid any perception of selective reporting, we present the figure in the supplementary materials. We added a clear note that the distribution should not be interpreted directly, but from a statistical physics perspective as a partition function (see supplementary materials “Magnetic Moment Evaluation of Standard Particle Samples”). The new peaks visible in that figure most likely originate from the fact that the selected magnetization model does not fully describe these features. We emphasised this in the main text by adding paragraph “These magnetic moment distributions should not be interpreted literally in all cases …” in “Evaluation of Magnetic Moment Resolution” section.

Reviewer #3 (Remarks to the Author):

I co-reviewed this manuscript with one of the reviewers who provided the listed reports. This is part of the Communications Engineering initiative to facilitate training in peer review and to provide appropriate recognition for Early Career Researchers who co-review manuscripts.

Thank you very much!
